# Computational chemoproteomics to understand the role of selected psychoactives in treating mental health indications

**DOI:** 10.1038/s41598-019-49515-0

**Published:** 2019-09-11

**Authors:** Jonathan Fine, Rachel Lackner, Ram Samudrala, Gaurav Chopra

**Affiliations:** 1 Department of Chemistry, Purdue University, West Lafayette, IN USA; 2 Department of Chemistry, University of Pennsylvania, Philadelphia, PA USA; 3 Purdue Institute for Drug Discovery, Purdue Institute for Integrative Neuroscience, Purdue Institute for Integrative Neuroscience, Purdue Institute for Immunology, Inflammation and Infectious Disease, Integrative Data Science Initiative, Purdue Center for Cancer Research, West Lafayette, IN USA; 4 Department of Biomedical Informatics, SUNY, Buffalo, NY USA

**Keywords:** Computational platforms and environments, Proteome informatics, Computer modelling, Neurological disorders, Cheminformatics

## Abstract

We have developed the Computational Analysis of Novel Drug Opportunities (CANDO) platform to infer homology of drug behaviour at a proteomic level by constructing and analysing structural compound-proteome interaction signatures of 3,733 compounds with 48,278 proteins in a shotgun manner. We applied the CANDO platform to predict putative therapeutic properties of 428 psychoactive compounds that belong to the phenylethylamine, tryptamine, and cannabinoid chemical classes for treating mental health indications. Our findings indicate that these 428 psychoactives are among the top-ranked predictions for a significant fraction of mental health indications, demonstrating a significant preference for treating such indications over non-mental health indications, relative to randomized controls. Also, we analysed the use of specific tryptamines for the treatment of sleeping disorders, bupropion for substance abuse disorders, and cannabinoids for epilepsy. Our innovative use of the CANDO platform may guide the identification and development of novel therapies for mental health indications and provide an understanding of their causal basis on a detailed mechanistic level. These predictions can be used to provide new leads for preclinical drug development for mental health and other neurological disorders.

## Introduction

Drug discovery traditionally revolves around single biological targets and focuses on a limited set of relationships between a protein target and small molecules of interest. The goal of this approach is to change the biological function of a protein responsible for pathogenesis and subsequently determine the toxicity and side effect profile of a compound to make it a suitable clinical candidate. The expected result of this approach is a compound that modulates the single protein that it targets. Although this traditional approach has been successfully applied to develop the majority of approved drugs, it has been questioned in recent years as the number of new approved drugs continues to decrease (currently down to 30 according to fda.gov). Additionally, many new drugs are analogues to already known drugs or reformulated to improve efficacy and filed as new patents. According to the Tufts Center for the study of Drug Development (csdd.tufts.edu), the average cost to bring a new drug to market can be as large as $2.6 billion. Therefore, there exists a shortage of novel drug development because the current approach is both time and cost prohibitive^1–4^.

One methodology to combat the rising cost and time commitment of novel drug development is to repurpose already approved drugs that are known to have few deleterious side effects^3,5–11^. Competiveness in the pharaceutical industry hinders the systematic exploration of potential repurposing opportunites, but computational approaches enable a workaround. Using computational multi-target docking with dynamics, we developed a drug repurposing approach for malaria^7^ and have since validated our models numerous times experimentally^3,8,9,12–17^. To expand the applicability of our work, we have developed a shotgun approach to evaluate all potential drug repurposing opportunities simultaneously by evaluating the relationships of compounds with entire proteomes (chemoproteome) in an indication-specific manner^9,15^. Here, we describe the application of our platform to identify possible therapeutic uses of phenethylamines, tryptamines, and cannabinoids in treating mental health indications.

### Leveraging computational chemoproteomics for drug discovery

Natural products have a profound impact on drug discovery. Many of these products come from plant sources^18–20^, where 60% of drugs approved by the FDA circa the 1990s came from these sources^21^. While this percentage has decreased to about 40% in recent years, it is clear that natural products have an important impact on drug discovery^22^. Since plants, animals, and other organisms have evolved together, we hypothesize that multiple modes of action are responsible for a small molecule to become a drug. We have thus developed a platform which relies on a “signature of interactions” (a row of binary or real numbers) to represent the interactions of compounds with a set of protein structures that are selected to represent the known structural universe. Our hypothosis requires that similar chemoproteome signatures indicate similar functional behvarious while non-similar signatures (or regioins thereof) indicate off and anti-target (side) effects as these signatures infer proteomic homology of compound or drug behavior. We can use these chemoproteomic signatures to rank how well a compound can be repurposed for given indication and provide a set of protein interactions responsible for this ranking to obtain an understanding of drug mechanisms at the level of atomic interactions.

### CANDO: A shotgun computational chemoproteomics platform for drug repurposing and discovery

Biologically active molecules, such as proteins and drugs, do not function in isolation. The absorption, dispersion, metabolism, and excretion (ADME) and effectiveness of a drug are dependent on the interactions of the drug with a system of proteins expressed at different sites in an organism. The Computational Analysis of Novel Drug Opportunities (CANDO) platform works at the proteomic level by leveraging the interaction signature of a compound to all proteins in a generic structural library. It compares the signatures of candidate compounds/drugs to those approved for particular indications to make drug repurposing predictions in a shotgun manner (here meaning an all vs. all compound-proteome signature comparison).

**Figure 1:**
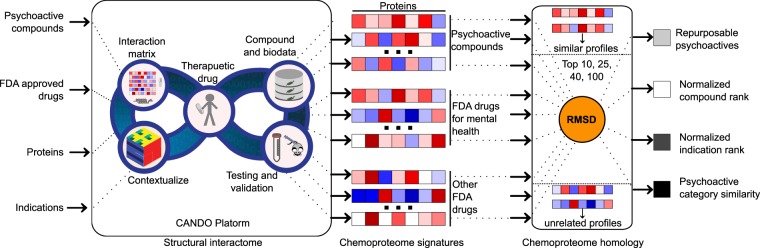
Schematic of computational chemoproteomics pipeline to identify psychoactives for mental-health indications using the CANDO platform. The version (v1) of the CANDO platform used in this study evaluated interactions between 3,733 human ingestible compounds (including the 428 psychoactives listed in Tables S1–S6) that are associated with 2030 indications (including 137 related to mental health and epilepsy disorders) and 48,278 protein structures (46,784 used to compute the structural interactome). The chemoproteomics interaction signatures are ranked according to the degree of interaction and similarity for all indications. The signature comparison (RMSD) and ranking approach (TopX predictions) yielded benchmarking accuracies of 12–25% for 1439 indications with at least two approved compounds. 58/163 (35%) top ranking predictions had comparable or better activity relative to existing drugs in twelve prospective *in vitro* studies across ten indications. We expect these findings to hold for evaluating the potential of psychoactives in treating mental health indications, which we then analysed holistically to determine global patterns and make predictions of putative drug leads for these indications.

The first version of the CANDO platform (CANDO v1) shown in Fig. 1 predicts interactions between 3,733 FDA approved drugs and a variety of other human ingestible compounds (including supplements and illegal substances) and 48,278 protein structures from multiple species (46,784 of which are used in this study and this protein list is provided in the GitHub data repository) either taken from the Protein Data Bank (PDB)^23^ or representing high confidence homology models^24^ constructed using protein structure prediction methods described previously^15,25^ Specifically, the proteins structures include solved and modeled proteins obtained from eukaryotic, prokaryotic, archaea and viral organismal proteomes, including 14,595 human proteins (8,841 of these are high-confidence models), a set of 24,958 nonredundant solved protein structures in the PDB, in addition to the remaining solved and modeled structures from *M*. *tuberculosis*, *P*. *aeruginosa*, and viral proteomes, etc. We consider different conformations of protein structures by separately including multiple domains (chains) and isoforms of proteins for calculating all compound proteins interactions. As an example, for the experimental structures considered for the human proteome, we use a mapping between PDB chains and UniProtKB/SwissProt codes^26^ in the human proteome. We also treat all such protein-compound interactions equally as proteins from different biological classes affect benchmarking accuracy results to predict putative repurposeable drugs for diseases^15^.

We employ our bioinformatic docking approach to construct a 3,733 × 46,784 compound-protein interaction matrix (see *Compound-Proteome Interaction Signature* section^15^) that is analysed to determine similarity in drug behavior^15,25^. No special methods were used for different protein classes (e.g., kinases and GPCRs) so that scores of two proteins from different classes could be compared directly. To generate a pose we used a hierarchical fragment-based docking with dynamics algorithm^27^ using knowledge-based potentials^28^ as done previously for the Ebola proteome^29^. We have previously shown that all-atom dynamics is necessary for accurate prediction of binding energies^30^ and demonstrated all-atom knowledge-based force fields are more accurate than physics-based approaches for both protein structure prediction and docking^16,17,31–34^. Furthermore, we have shown that multi-targeted docking with dynamics leads to improved hit rates for finding inhibitors of pathogens relative to conventional approaches^7,8^. It should be noted that the interaction score stored in this matrix does not represent whether a given target will be inhibited or activated, only that the compound and target interact. As a result, the CANDO platform can be used for both inhibitors and agonists with the caveat that the predicted effect of a compound may be unknown until verified experimentally. For example, CANDO could predict cocaine for the treatment of cocaine-related disorders. Therefore, special care needs to be used when examining these predictions since dose selection is not part of the current model.

Once the interaction matrix is constructed, our methods compare the all compound-proteome interaction signatures where the similarity of two signatures can be calculated using various metrics as simple as root mean squared deviations (RMSD) to sophisticated graph theory based comparisons that can take underlying protein-protein interactions (compiled from public sources^24,35–37^) into account. Similarities between (regions of) interaction signatures indicate a relationship in functional behaviour. However, the differences between two signatures are difficult to understand without further knowledge as it may indicate a more potent drug, a possible side effect, or no effect whatsoever. In addition to predicting a ranked list of putative drugs that are most likely to function similarly to other drugs approved for a particular indication, the signature comparison and ranking helps to analyse compound behaviour in biologically relevant pathways^35,36,38^. Our CANDO platform is successful for prospectively validating putative leads for several indications^15,25,29^.

### Mental health indications and interventions

A large number of diseases and disorders have mental health implications as catalogued by the American Psychiatric Association (APA)^39^. These indications affect people in all age groups, social classes, and races^40–44^. The treatments for these indications mostly consist of small molecule therapeutics, varying individually for specific diseases, disorders, or conditions. According to a report published by the World Health Organization in 2011^45^, the number of United States (US) citizens taking medication to treat mental health has increased to over two million US citizens since 2001. Anxiety disorders make up the largest category of mental illness in the US affecting a total of 42 million people. The second largest category is major depression disorder affecting 14.8 million US citizens on any given day. Approximately 2.4 million US citizens have schizophrenia where no effective treatment or cure is currently available as schizophrenia medication typically results in metabolic issues leading to weight gain and type 2 diabetes^46^. Collectively mental health indications/disorders cost the US economy $192.3 billion each year and result in high morbidity, with suicide being the tenth largest cause of death^47,48^. Unfortunately, adolescents are susceptible to depression and suicide, and the effectiveness of antidepressants for these individuals remains uncertain^49^.

### Human use of psychoactive substances

We define psychoactives as compounds that cross the blood-brain barrier, target proteins expressed in the brain as their primary modes of action, and thereby perturb human mental states. Although proteins expressed in the brain are paramount for the prediction of compounds as potential therapies for mental health disorders, synergistic effects may occur due to interactions in the periphery. For example, it has been shown that the gut microbiome plays an important role in the central nervous system^50^ and multiple links between the peripheral mechanisms and depression have been found previously^51,52^. We have also benchmarked CANDO to show that best drug repurposing accuracies are obtained when all protein structures are used for interaction signature comparisons to determine compound similarity, suggesting the role of multiple networks working together in biology to achieve a certain phenotype/function, instead of specific proteins as used traditionally for drug discovery^15,25^. This approach makes CANDO different than other methods that are focused towards single target inhibitor discovery vs drug discovery. Therefore, we believe that the study of proteome-wide interaction signature for repurposing psychoactive compounds is suitable in the context of mental health indications.

Since the time of the earliest records, humans have been ingesting psychoactive substances for religious and spiritual purposes (for example, dimethyltryptamine in Ayahuasca, mescaline in Peyote), for medicinal purposes (opium), and for recreation (caffeine, nicotine, alcohol)^53^. The vast majority of pyschoactives are considered taboo for a variety of reasons and, with few exceptions, are not investigated for potential medicinal properties. In this study, we focus on the phenylethylamine and tryptamine classes of psychoactives described by Alexander Shulgin^54,55^ as well as additional cannabinoids.

Due to recent changes in legislation, a few of these compounds are available as approved drugs in some jurisdictions (for example amphetamine for diet control and attention deficit hyperactive disorder, and tetrahydrocannabinol for anxiety). The action of these compounds is thought to affect human physiology by their structural similarity/mimicry to neurotransmitters (for example, psilocybin and lysergic acid diethylamide both mimic the compound serotonin). There is an increasing amount of evidence for cannabinoids having the ability to treat epilepsy and epilepsy-related indications^56,57^, but its legal status is still diffuse as cannabinoids remain classified as Schedule I by the United States Federal Government (a classification possessing no medicinal use). Similarly, psilocybin and ketamine have been shown to treat depression via a mechanism not targeted by current antidepressants^58,59^.

These examples are the tip of a proverbial iceberg, and recent reinvestigations into the clinical relevance of illicit psychoactive compounds suggest further investigation into the potential of these compounds in treating mental health indications^60^. This clear disconnect between current research and current legislation warrants a more comprehensive investigation for the use of these psychoactive compounds for medicinal purposes but *in vitro* and *in vivo* verification is currently difficult given their scheduling status. The CANDO shotgun drug discovery and repurposing platform is therefore uniquely suited to conduct such an investigation to make a case for experimental verification.

While other classifications of psychoactives could be utilized (for example, all compounds known to cross the blood-brain barrier), our goal in this study was to see if any of the selected psychoactive compounds, primarily known without any therapeutic utility, are predicted to treat mental health indications. Our work also demonstrates the more general utility of the CANDO platform in assessing the effect of drug classes on this specific class of indications.

### Analysing the role of psychoactives in mental health indications using CANDO

Most of our selected psychoactive compounds are illegal to synthesize and thus difficult to study *in vitro* (much less *in vivo*). Cannabinoids are in the process of being legalized for medicinal uses in some jurisdictions, and this serves as a justification for studying these drugs further. The cause of many mental health indications is not characterized by one protein, but by several proteins in several different categories^61–65^. Thus, the traditional high throughput screening methodology of testing one compound against one protein is not a suitable approach for mental health drug discovery. The CANDO platform allows evaluation of all selected psychoactives across a large library of protein structures, providing a logical and reasonable method to develop leads for medications that may be suitable for treating mental health indications. Our goal here is to study, analyse, and characterize these psychoactive compounds using the CANDO platform so that the potential medicinal properties of these compounds can be assessed and evaluated in further bench and clinical studies. The outcomes for this study are not necessarily to predict mental health therapies but rather to generate hypotheses if the predicted psychoactives serve as the most promising leads for different mental health indications based on similar chemoproteomics perspective.

## Results

We describe our results based on two approaches of examining the relationships between the selected psychoactives and mental health indications using the top-ranked predictions by the CANDO platform. An example of these predictions is given in Table 1, where we are careful to list potential issues with the predicted psychoactive. At the outset, we examined the distributions of percentages of psychoactive compounds (relative to total compounds) in the top-ranked predictions for mental health indications. Conversely, we can compare the distributions of percentages of mental health indications selected by the psychoactive compounds in the top-ranked predictions. We further analyse the latter distributions broken down by psychoactive classes and the distributions of mental health indications. We conclude with three case studies illustrating the application and utility of the CANDO platform in discovering psychoactive therapeutics to treat mental health indications. We again caution that applying these predictions for the development of new therapeutics must be done judiciously.

**Table 1:** Psychoactives and their corresponding mental health indications with the highest ranks from the Top10 predictions.

Psychoactive	Known effects and potential for abuse	Mental Health Indication
3,4-dimethylmethcathinone	Stimulant with a high potential for abuse.	Anxiety Disorders
3,4-dimethylmethcathinone	Stimulant with a high potential for abuse.	Depressive Disorder, Major
1-naphthyl(1-pentyl-1h-indol-3-yl)methanone	Serious source of addiction	Alzheimer Disease
dextromethorphan	Over the counter antitussive	Attention Deficit Disorder with Hyperactivity
dexfenfluramine	Weight loss drug pulled for causing cardiac issues	Autistic Disorder
3-fluoroamphetamine		Bipolar Disorder
2-fluoroamphetamine		Cataplexy
metamfepramone		Delirium
bupropion	Approved by the US FDA to treat depression	Depressive Disorder
metamfepramone		Tourette Syndrome
ergoline	Used to treat migraine headaches	Erectile Dysfunction
α-pyrrolidinopentiophenone	Stimulant	Learning Disorders
2-fluoroamphetamine		Narcolepsy
isopropylamphetamine		Obessive-compulsive disorder
3-fluoroamphetamine		Personality Disorders
pyrovalerone	US Schedule V drug used for treatment of chronic fatigue	Phobic Disorders
dextromethorphan	Over the counter antitussive	Psychotic Disorders
3,4-dimethylmethcathinone		Restless Legs Syndrome
pyrovalerone	US Schedule V drug used for treatment of chronic fatigue	Schizophrenia
pyrovalerone	US Schedule V drug used for treatment of chronic fatigue	Stress Disorders, Post-Traumatic
α-pyrrolidinopentiophenone	Stimulant	Substance Withdrawal Syndrome
pyrovalerone	US Schedule V drug used for treatment of chronic fatigue	Tobacco Use Disorder
isopropylamphetamine		Panic Disorder
3,4-dimethylmethcathinone	Stimulant with a high potential for abuse.	Cocaine-Related Disorder
3-fluoroamphetamine		Binge-Eating Disorder

This represents a small window into the types of predictions made using our drug discovery and repurposing platform. These predictions represent hypotheses of novel putative therapeutic leads for these indications to be further evaluated by preclinical and clinical experiments.

### Putative psychoactives for mental health indications

**Figure 2:**
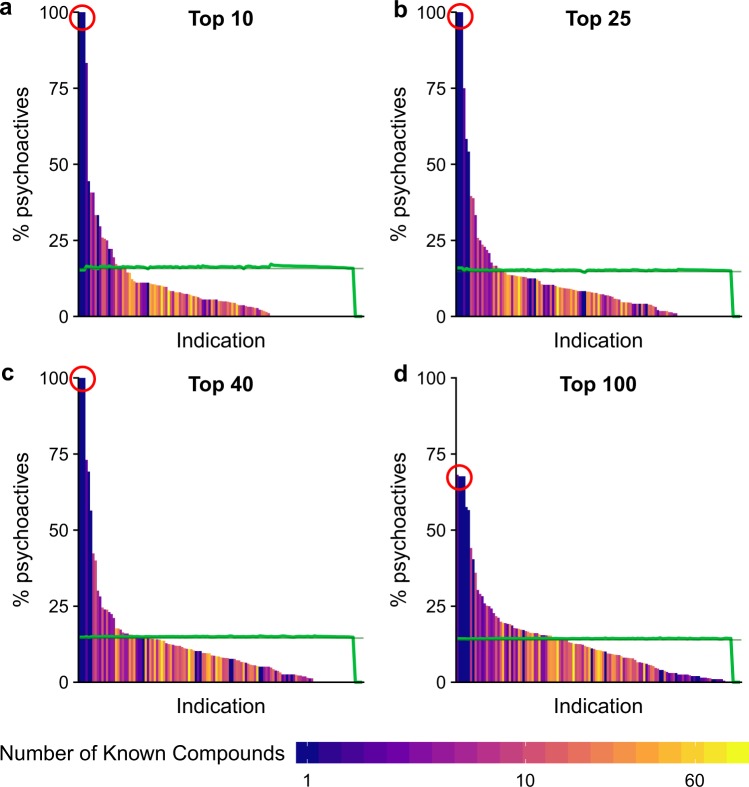
Normalized indication rank for all indications: Percentage of psychoactives in the Top10 (**a**), Top25 (**b**), Top40 (**c**), and Top100 (**d**) ranked predictions for mental health indications. The shading of the bars indicates the number of compounds known to treat an indication in the ranked lists. The green line shows the results of randomizing the predicted compounds and the straight-line segment indicates the mean of the randomized distribution. A one-tailed Kolmogorov-Smirnov test is performed to show that these distributions are statistically different than the randomized distributions (p < 0.001) Raw KS-p-values are presented in Table S10. The data indicate that the prediction of psychoactives by the CANDO platform for mental health indications is not due to chance and that a significant fraction of mental health indications is much more likely to be amenable to treatment by them or an analogously behaving compound (left-hand side of the graphs above the green control line). The circled indications are Seasonal Affective Disorder, Circadian Rhythm Sleep Disorders, and Jet Lag Syndrome.

The results showing the distributions of percentages of psychoactives for mental health indications are given in Fig. 2, and the values used to create the figure are provided in Table S7. This figure shows that the difference in the random and non-random distributions. Since these distributions are statistically different, we conclude the selected psychoactive compounds are better at treating mental health indications on average than non-psychoactive compounds selected at random. As the number of compounds considered increases, the normal and randomized distributions become more alike. This result is expected as there are a larger number of non-psychoactive compounds than the selected psychoactive ones and, therefore, the addition of a new compound is more likely to be non- psychoactive than psychoactive. Therefore, as the number of compounds in the result list increases the percentage of psychoactives predicted for any indication will decrease (Fig. 2a–d).

### Selection of mental health indications by selected psychoactives

**Figure 3:**
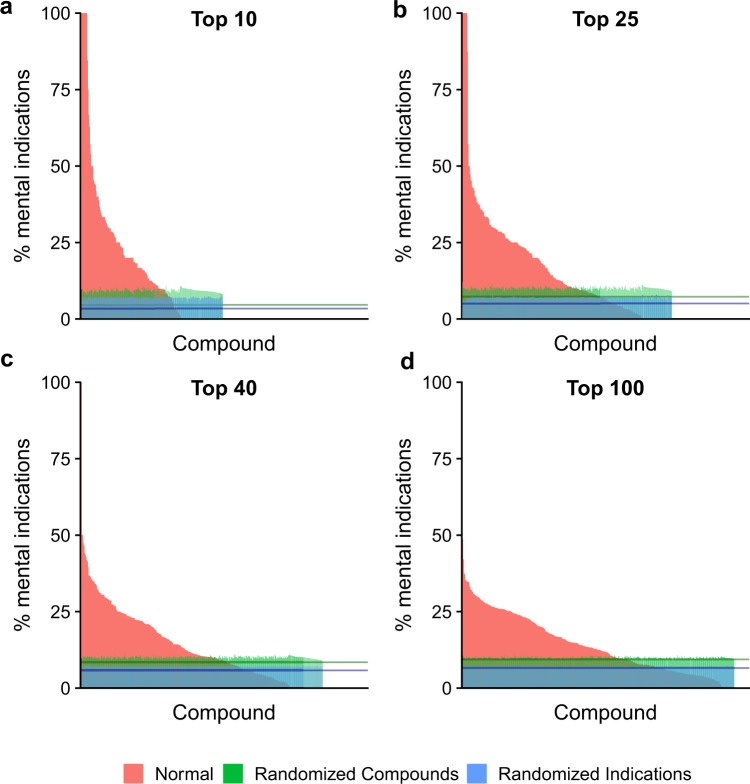
Normalized compound rank for all psychoactives: Percentages of mental health indications predicted to be treated by psychoactives for the Top10 (**a**), Top25 (**b**), Top40 (**c**), and Top100 (**d**) ranked predictions. Bars shown in red indicate non-randomized results, bars in green indicate the same results after the compounds in the ranked lists have been randomized, and bars in blue show the percentages after (only) the indications have been randomized in the ranked lists. The green line segment indicates the mean of the randomized psychoactive distribution and the blue line segment indicates the mean of the randomized indication distribution. A one-tailed Kolmogorov-Smirnov test for the normal (red) distribution against the randomized (green) one shows statistical significance (p < 0.000001) for each of the panels above. Raw KS p-values are presented in Table S10. In addition, raw T-test p-values are given in Table S11. The data indicate that the psychoactive compounds select mental health indications within their top rankings at a much higher likelihood than compounds selected at random.

The distributions for the selection of mental health indications by selected psychoactives relative to all indications are shown in Fig. 3 (raw values provided in Table S8). The greater the percentage of mental health indications, the more selective the psychoactive. Furthermore, the indications selected by psychoactives using the CANDO platform yield a high percentage of mental health indications relative to random controls, illustrating that these psychoactives are more likely than non-psychoactives to be effective at treating mental health indications.

### Comparison of randomized compound and indication distributions

The two randomized distributions in Fig. 3 (shown in green and blue) are distinct. The distribution representing randomized compounds is less uniform and has a larger average percentage (p-value less than 2 × 10^−16^ from a one-tailed student t-test for all four plots) than the randomized indication distribution. These data show that a single drug is more likely to treat multiple indications than a single indication is to be treated by multiple drugs. This has been shown previously by the ability to repurpose drugs^66–68^ and is an important feature of the CANDO platform. The ability to repurpose previously approved compounds is increasingly important^69^. This result highlights the utility of the CANDO platform for drug repurposing.

### Comparison of different psychoactive classes

**Figure 4:**
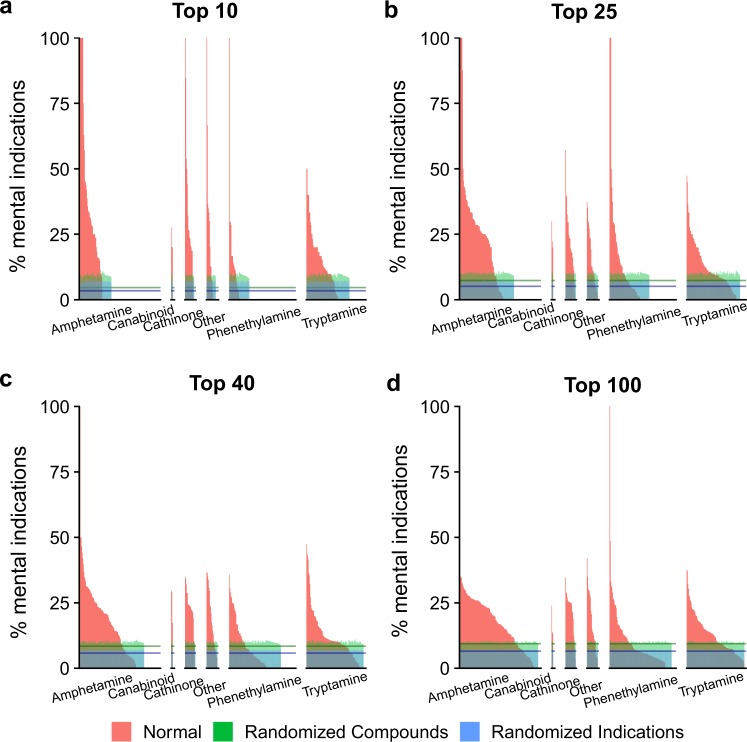
Percentage of mental health indications predicted to be treated by classes of psychoactive compounds according for the Top10 (**a**), Top25 (**b**), Top40 (**c**), and Top100 (**d**) ranked predictions. The above graph shows the percentage of indications selected by psychoactives (similar to the previous figure), except that the psychoactives have been grouped into six major classes: amphetamine, cannabinoids, cathinones, phenethylamines, tryptamines, and other (left to right in each panel). The data illustrate the selectivity of each of these three classes of compounds for mental health indications.

**Figure 5:**
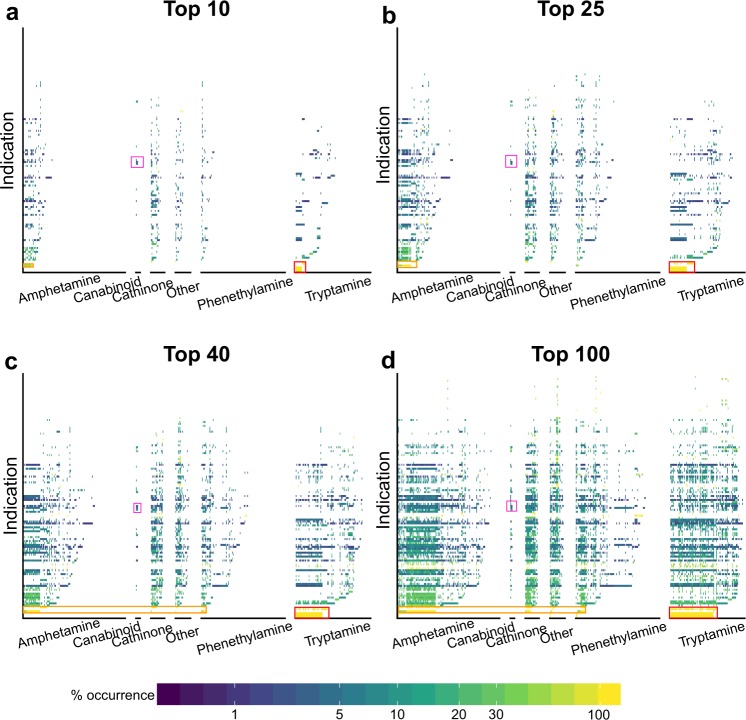
Distribution of mental health indications treated by different classes of psychoactives. As the number of predictions increases (from Top10 (**a**) to Top100 (**d**)), the distribution of indications per class becomes increasingly similar. Given the proteomic signature comparison approach used by CANDO to makes these predictions, this indicates that psychoactives from one category are predicted to bind to the same proteins as psychoactives from a different category, resulting in a constant percent occurrence for all compounds predicted to treat an indication. Thus, the Top10 rankings provide the most specificity for analysing the effect of a psychoactive class on selecting mental health indications. Indications of interest are shown with the following boxes: red for Seasonal Affective Disorder, Jet Lag Syndrome, sleep disorders and Broca Aphasia; orange is Binge-Eating Disorder, Narcolepsy, and Anorexia Nervosa; purple is Heroin Dependence, Substance-Related Disorders, and Epilepsy.

Figures 4 and 5, Tables S8 and S9 differentiate the psychoactives by compound class: amphetamine, cannabinoids, cathinones, phenethylamines, and tryptamines. These figures and tables illustrate that the classification of a compound has an impact on which indications it is predicted to treat. Therefore, we will continue the discussion based on psychoactive compound-classes. The details to classify psychoactive compounds used in this work is given in the Supporting Information section entitled: “Classification of psychoactive compounds via substructure searching”.

### Relationships between mental health indications

**Figure 6:**
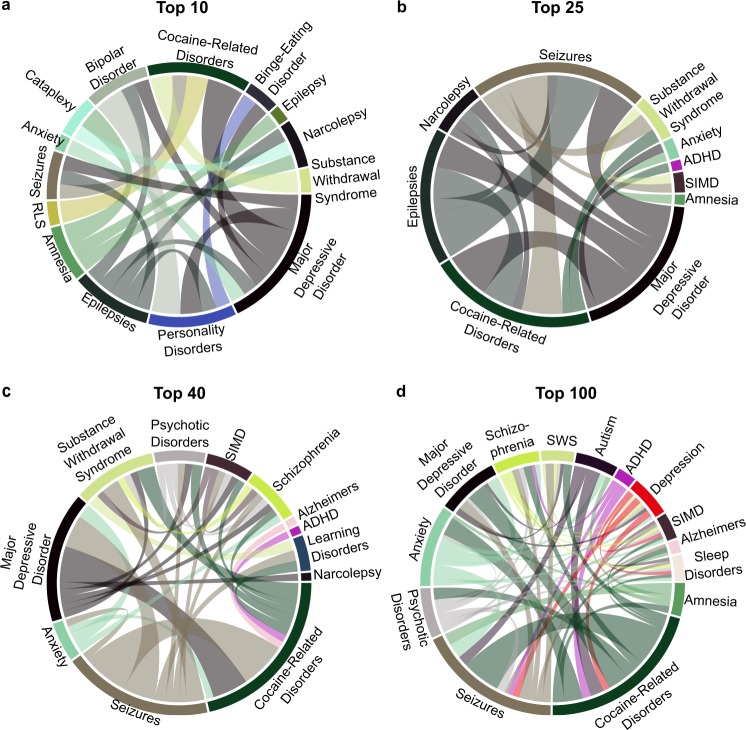
Indication-Indication association counts plotted as chord diagrams for all Top sets (**a**–**d)**. Raw association counts are given in Tables S12–S15. These diagrams show predicted relationships between the indications. The width of the chord is proportional to the number of predicted psychoactives relating two indications. Known relationships include Epilepsy with Seizure, Depression with Cocaine-related disorders, etc. Abbreviations used: RLS: Restless leg syndrome, SIMD: sleep initiation and maintenance disorder, ADHD: Attention deficit with hyperactivity disorder, SWS: substance withdrawal syndrome.

Our predictions for indication-indication associations are shown in Fig. 6. Interestingly, some indication relationships have been verified clinically. These include: Epilepsy with Seizure, Cocaine-related disorders with depression^70^, Seizures with Substance Withdrawal Syndrome^71^, Depression with Anxiety,^72^ and possibly relating binge-eating and personality disorder^73^. The ability of our repurposing platform to reproduce known indication relationships suggests that our chemoproteomic signatures can capture key biological interactions. In addition, the number of overlapping psychoactive compound predictions strongly relate multiple mental health indications (width of the chords in Fig. 6). These psychoactives interact with multiple proteins (similar chemo-proteome signatures) suggesting common biochemical pathways. We are confident that our method may be useful to discover new disease pathways relating these indications. Identifying and validating these new pathways are beyond the scope of this work.

## Discussion

The Top10, Top25, and Top40 predictions in Fig. 2 for three mental health indications, Seasonal Affective Disorder, Circadian Rhythm Sleep Disorders, and Jet Lag Syndrome, consist only of psychoactives belonging to the tryptamine class (indication rank of 100%). The only compound known to treat all these indications is melatonin (also a tryptamine)^74–76^, indicating that its proteomic interaction signature is most similar to the interaction signatures for these predicted psychoactives. This result demonstrates that the proteomic shotgun drug repurposing approach adopted by the CANDO platform makes sensible predictions of related compounds based on their similarly of interaction signature with all proteins, compared to traditional single target approaches. As a result, we present class specific breakdowns of Fig. 2 in the Supporting information (Fig. S1). Studies by an Israeli pharmaceutical company give experimental evidence demonstrating that some of these tryptamine psychoactives are indeed likely to treat the aforementioned three indications^77^. These studies provide corroborative evidence for the efficacy of the CANDO platform and highlight its potential of finding new drugs for treating any indication that has at least one approved drug.

The remainder of this discussion will be used to highlight case studies which are verified in the literature. For a complete list of psychoactive predictions, please see the tables in the supporting information.

The indication with the largest number of high ranking psychoactives in the top-ranked predictions is cocaine-related disorders belonging to the cathinone class of stimulants, a summary of which is given in Table 2. The similarity between the effects of cathinone and cocaine on behaviour has been previously established as part of a similar pathway^78^. We are aware that some of these predictions are unlikely to have any potential for the development of new therapeutics for cocaine-related disorders due to their associated toxicity^79,80^. A cathinone of interest is the anti-depressant bupropion, which is well known for promoting smoking cessation and has also been proposed for the treatment of methamphetamine and cocaine substance abuse disorders^81,82^. These findings and related uses further showcase the ability of CANDO platform to accurately associate compounds/drugs and indications. While this example is successful in showcasing CANDO’s ability to find the relationship between compounds and mental health disorders, one needs to be cautious as these predictions may mimic cocaine and lead to adverse reactions depending on the dose. For example, Bupropion is perceived as a stimulant to those with a history of cocaine use^83,84^. Further, the effects of dextromethorphan may be due to its stimulant properties^85^. However, in some cases we can obtain therapeutic benefit from potentially problematic compounds, e.g. methadone is an approved treatment for opioid abuse, but is known to have several opioid-related effects when given in high enough dosages^86^.

**Table 2:** Top psychoactives predicted to treat cocaine-related disorders by the CANDO platform.

Psychoactive	Known effects and legal status
flephedrone	Toxicity not well established
buphedrone	Illegal for human consumption
ethcathinone	Illegal due to similarities to mephedrone
mephedrone	High potential for abuse
methcathinone	Causes euphoria. Highly addictive
3,4-dimethylmethcathinone	Stimulant with a high potential for abuse
bupropion	Prescription anti-depressant.
dextromethorphan	Over the counter antitussive
alpha-pyrrolidinopropiophenone	Stimulant
1-naphthyl(1-pentyl-1h-indol-3-yl)methanone	Serious source of addiction
n,n-dibutyltryptamine	Hallucinogenic research chemical
isopropylamphetamine	Stimulant

The top predictions for these indications belong to the cathinone class. A few of these compounds have already been associated with cocaine-related disorders in the literature^78–82,87–90^, showcasing the accuracy of the platform in rediscovering known associations, and the remaining ones represent hypotheses of novel putative therapeutic leads to treat cocaine-related disorders to be evaluated by further preclinical and clinical experiments.

The highest-ranking phenethylamine predicted to treat cocaine-related disorders is the antitussive drug, dextromethorphan. This compound, generally available over the counter, is known for its hallucinogenic side effects at high doses, which is reflected both in the predictions by CANDO and is reinforced in the literature^87–91^. The use of the CANDO platform for making predictions to treat specific mental health indications is strengthened by the accurate identification of bupropion and dextromethorphan (both selected psychoactives) in treating cocaine-related disorders.

The two psychoactive cannabinoids, tetrahydrocannabinol and cannabinol are predicted to treat Epilepsy and Absence Epilepsy by the CANDO platform, and cannabinol is also predicted to treat Status Epilepticus. While the cannabinoids are not the highest ranked compounds relative to other psychoactives for these indications, our findings are validated by recently published studies for the use of cannabinoids to treat epilepsy-related indications^56,57^. The non-psychoactive cannabinoid (cannabidiol) is not predicted to treat any epilepsy-related indications, leading to an intriguing hypothesis concerning the likelihood of a cannabinoid treating epilepsy corresponding to its psychoactivity. However, given the limited data available, further study is warranted to verify this hypothesis. Our work illustrates the recovery of known corroborative associations between cannabinoids and epilepsy but also demonstrates how predictions made by the CANDO platform can be used to develop hypotheses on the biology of diseases for experimental investigation.

## Methods

An overview of the CANDO platform is described in Supporting Information. Here, we describe the approach used to analyse the data generated by this platform to characterize the role of the selected psychoactives in mental health indications.

### Selection of specific phenethylamines, tryptamines, and cannabinoids

We collected a total of 428 compounds (structures in Tables S1–S6) to be investigated using CANDO and categorized them into 291 phenethylamines and 109 tryptamines described by Alexander Shulgin^54,55^, and 6 cannabinoids (cannabinol, cannabidiol, and tetrahydrocannabinol) using a subgraph based search methodology based on the structure of the parent molecule (see Supporting Information for full description of this method). An additional 22 compounds are not strictly classified as phenethylamines but have structural similarity to the phenethylamine class are included as unclassified. We further subdivided the 291 phenethylamine compounds into 149 amphetamines and 20 cathinones, the remaining 122 phenethylamines are simply referred to as phenethylamines. The CANDO v1 compound library includes these 428 psychoactives and their proteomic interactions signatures to repurpose psychoactives for indications/diseases^9^. Most of these psychoactives are classified as Schedule I substances by the United States Drug Enforcement Agency, indicating they have no known medicinal use, no accepted standards for safety, or have a high potential for abuse. Thus, when such a substance is discussed, the potential pitfalls are presented along with that substance. We selected this set of compounds as almost all of them are known to affect mental physiology upon ingestion^54,55,92^. A notable exception in the compounds evaluated is cannabidiol which is not strictly psychoactive^92^ but is structurally similar to other cannabinoids and therefore warrants an investigation into its potential therapeutic value.

The CANDO v1 compound-proteome interaction signature (see Supporting Methods) includes all associations of treatment and side effects caused for each compound via the proteomic signature as this is composed of all target, anti-target, and off-targets proteins for each indication/disease. The compound proteomic interaction signature similarity yields therapeutic predictions by considering similarity to known drug signatures for each disease. It should be noted that this methodology can also match a psychoactive to a compound known to worsen a given indication in addition to predicting a compound known to ameliorate the same indication. Therefore, the set of compounds that were used as therapy for a given indication did not include any of the aforementioned psychoactive compounds given that the nature of these compounds as treatments is still controversial. As a result, the ability of the platform to predict a psychoactive from another psychoactive compound-proteome signature is not investigated in this work. Most importantly, all predictions are made based on similarity to an approved non-psychoactive drug for a mental health indication, without any knowledge of therapeutic target associations for making predictions for psychoactive compounds. Therefore, no association between an indication and a protein target is used to weight the similarity between two compounds. For example, the interaction score of a psychoactive and the dopamine receptor is not given a special weight for Schizophrenia.

### Selection of mental health indications

The Medical Subject Headings (MeSH) vocabulary is used to specify the diseases, disorders, and conditions that are classified as mental health indications. The U.S. National Laboratory of Medicine division of the National Institutes of Health (www.nlm.nih.gov) includes the latest version of the MeSH database. It should be noted that this database is compiled at the clinical level and does not consider the underlying biology leading to a specific indication. Therefore, some spurious and non-traditional indications may be included as mental health indications. Since a biological mechanism study is beyond the scope of this paper, we used all the indications suggested by MeSH.

The MeSH database is divided into tree structures with a specific tree (F03) denoted for Mental Disorders. The specific branches of the Mental Disorder Tree used in this study are Anxiety Disorders (F03.080), Dissociative Disorders (F03.300), Feeding and Eating Disorders (F03.400), Neurocognitive Disorders (F03.615), Somatoform Disorders (F03.875), Conduct Disorders (F03.250), Neurodevelopmental Disorders (F03.625), Mood Disorders (F03.600), Neurotic Disorders (F03.650), Personality Disorders (F03.675), Schizophrenia Spectrum Disorders (F03.700), Sleep-Wake Disorders (F03.870), and Substance-Related Disorders (F03.900). All the indications listed in these branches were used along with Dyspareunia, Erectile Dysfunction, Paraphilias, Fetishism, and Paedophilia from the Sexual Dysfunctions (F03.835) branch yielding a total of 108 mental health indications that are analyzed in this work. A separate MeSH identification paradigm was done for epilepsy-related indications as these indications are placed in a separate MeSH tree because they are neurological disorders, not psychiatric disorders. The MeSH tree evaluated for epilepsy is C10.228.140.490 which includes Drug-Resistant Epilepsy, Myoclonic Epilepsies, Partial Epilepsies, Benign Neonatal Epilepsy, Generalized Epilepsy, Post-Traumatic Epilepsy, Reflex Epilepsy, Landau-Kleffner Syndrome, Lennox-Gastaut Syndrome, Seizures, and Febrile Seizures. A total of 29 additional epilepsy-related indications are presented in this work.

## Calculation

### Ranking the importance of predicted psychoactives for mental health indications

We generated Top10, Top25, Top40, and Top100 ranked compound lists for all indications (mental health related and otherwise) using the CANDO v1 platform for each indication and counted the number of times a compound prediction is present in each of the ranked lists. A compound may be predicted to treat an indication several times if there are numerous known drugs for that indication.

For example, 65 known drugs are used clinically for schizophrenia and are included in the CANDO platform. Therefore, a set of 65 chemoproteomic signature similarities are used to predict an uncharacterized compound for schizophrenia. It is possible that the same uncharacterized compound may be predicted at most 65 times for schizophrenia. The number of times such a compound is predicted for a given indication is termed as the ‘consensus count,’ which is normalized as the percent occurrence. We compute percent occurrence as the ratio of consensus count to the maximum number of times a compound could be predicted for an indication using signature similarity (i.e. the number of known treatments for the indication).

We hypothesized that the higher the number of times a compound is predicted to treat a given indication, the greater the confidence in the prediction made because different drugs treat indications due to different biological pathways on the proteomic level. The combination of proteomic similarity implicitly includes a combination of all pathways to yield efficacy and is denoted by the frequency of compounds predicted for each indication. To investigate the general role of psychoactives for mental health, we took the frequency of psychoactive compounds predicted as putative drugs for each indication as a percentage of all compounds predicted for the given indication. The ratio of psychoactive to all compounds for a given indication is referred to as the normalized indication rank and quantifies the overall performance of psychoactive compounds versus non-psychoactive for a given indication.

A similar procedure is used to measure the propensity of a compound to be predicted for mental health indications and is used to ask the question: how many times an indication is (or percentage of mental health and non-mental health indications) listed either as a prediction for treatment by each psychoactive compound and is referred to as the normalized compound rank. This measure allows us to express the preference of a given compound towards mental health indications. To illustrate metrics of normalized compound rank, and normalized indication rank, we consider a simple example where a psychoactive compound ergoline and a non-psychoactive compound aspirin are only predicted for the mental health indication Pica (an eating disorder) and Stomach pain (non-mental health indication). Now consider ergoline that is predicted seven times for Pica, and three times for Stomach pain based on the similarity of chemo-proteome analysis while the non-psychoactive drug aspirin is predicted to treat Pica four times and Stomach pain six times. The normalized compound rank for this simple example will be 70% [100 * 7/(7 + 3)] for ergoline and 40% [100 * 4/(4 + 6)] for aspirin. Using the above example, we can also determine the normalized indication rank for Pica and Stomach pain. Since seven psychoactives and four non-psychoactive compounds were predicted for Pica, the normalized indication rank is 64% [100 * 7/(4 + 7)] for Pica and similar calculation yields an indication rank of 33% for Stomach pain. Together these metrics suggest the importance of psychoactive compounds and their preference for mental health compared to non-mental health indications.

### Computational randomized controls

To further ensure that our results were not arrived at by chance, the order of the predicted compounds is randomized, and the above procedures repeated. All compounds predicted to treat an indication are randomized regardless of whether the indication is categorized as a mental health indication. Thus, a compound not predicted initially to treat a mental health indication may, due to random chance, be predicted to treat a mental health indication in the randomized data set. If a random compound replaces a predicted compound multiple times for a single indication, the random compound replaces the original (non-random) compound for every prediction. This randomization process is repeated 1000 times, and the compound and indication ranks for all the randomized searches are averaged.

A second random control is performed in addition to the one described above where the indications (mental health or otherwise) are randomly rearranged. Thus, a non-mental health indication may be classified as a mental health indication by chance (and vice versa). This procedure provides a second control that allows us to assess whether selected psychoactives are more likely to be predicted for mental health indications than non-mental health indications.

### Determination of relationships between mental health indications

We relate two mental health indications when at least two different psychoactive compounds are predicted for both indications. The frequency of prediction for common psychoactive compound predictions is termed as ‘indication-indication association counts.’ Next, to strongly relate the two indications, we also calculated the ‘consensus count’ for all psychoactives predicted for each mental health indication. Note that a predicted psychoactive could have a different consensus count for each indication. For example, 1-naphthyl(1-pentyl-1H-indole-3-yl) methanone is predicted 10 times for seizures and 8 times for sleep initiation and maintenance disorders (SIMD). Therefore, the consensus count of 1-naphthyl(1-pentyl-1H-indole-3-yl) methanone for seizures is 10 and 8 for SIMD. Another compound, 2-(5-methoxy-2-methyl-1H-indole-3yl)-n,n-dimethyl ethanamine has consensus count of 3 for seizures and 2 for SIMD. Since two different compounds are common predictions for the two indications, the indication-indication association count for these two indications is 2. To strongly relate the indications in Top lists and limit a large number of associations, we selected indication pairs with predicted psychoactive compound consensus count as follows: >=2 for the Top10 set, >=3 for the Top25 set, >=4 for the Top40 set, and >=6 for the Top100 set.

### Tests for statistical significance

A one-tailed Kolmogorov-Smirnov test^93^ was used to compare the distributions of psychoactives in the randomized and non-random distributions as this statistical test is typically used to show two distributions are dissimilar. For all statistical tests performed in this work, we formulated the *null hypothesis* to be that the distribution of psychoactives predicted to treat mental health disorders can be obtained by chance. Our alternative hypothesis is that the true distribution of psychoactives is greater than the randomized control (hence a one-tailed test). We also performed a one-tailed paired T-test to ensure that the mean of the differences between the test distribution and the randomized distribution is greater than zero. The raw p-values obtained for all statistical tests are given in Tables S10–S11.

## Conclusions

Traditional drug discovery is limited by its narrow focus on one or a few targets. Drugs approved for one indication interact with multiple proteins and thereby work across multiple indications. The CANDO platform improves upon the traditional approach by examining all interactions between a compound and a universal proteome. This novel approach enables the study of drugs in a holistic chemoproteomic manner that is especially relevant for the development of compounds intended for treating mental health indications as these complex disorders are mediated by multiple proteins and pathways. In this study, we investigated the compounds previously described by Alexander Shulgin along with additional cannabinoids to identify potential therapies for mental health indications. The results of this study indicate the selected psychoactive compounds perform better than compounds selected at random for mental health indications.

Conversely, the percentage of mental health indications selected by psychoactives is better than randomly selected compounds. This shows that psychoactives may represent promising leads for the development of therapeutics for the treatment of mental health indications. Specifically, the set shows promising results for sleep-related disorders, binge eating disorders, seasonal affective disorder, and cocaine substance abuse disorder. In addition, the other non-psychoactive compounds predicted by the CANDO platform present in the top-ranked predictions may also represent putative repurposable therapies for mental health indications, which will be explored in future studies. In a broader context, our work illustrates the advantages of using a computational chemoproteomics approach for drug discovery and repurposing by providing mechanistic information on which proteins are involved in the mediation of the therapeutic effect.

## Supplementary information

Supporting Information

## Data Availability

The 3,733 × 46,784 compound-protein interaction matrix, canpredict executables to calculate similarity with additional dependency files, output from canpredict for top10, top25, top40, top100, files for analysis, SMILES for 3,733 compounds, entire 46,784 list of proteins, R workspace and analysis scripts used to generate all figures presented in this work are available at http://github.com/chopralab/candiy_fun.git.
