# Supplementary material for: Computational chemoproteomics to understand the role of selected psychoactives in treating mental health indications
Source: Sci Rep. 2019 Sep 11;9:13155. doi: 10.1038/s41598-019-49515-0 (PMC6739337; doi:10.1038/s41598-019-49515-0)
Supplement: Supplementary file 1 — Supporting Information [file 41598_2019_49515_MOESM1_ESM.docx]

Computational chemoproteomics to understand the role of selected psychoactives in treating mental health indications

# Jonathan Fine, Rachel Lackner, Ram Samudrala, Gaurav Chopra

# **Supporting Information**

# **Overview of drug discovery**

The traditional drug discovery pipeline is initiated via high-throughput assays of large compound libraries *in vitro* against protein targets implicated in a specific disease/indication ^1–5^. Subsets of compounds that are active against targeted proteins (referred to as hits) are further studied using cell and animal models of the indication (when available). The pharmacokinetic and pharmacodynamic properties of the hits are determined at this stage. The few compounds that are efficacious in treating the indications *in vivo* go through structural optimization, and detailed toxicity profiles are derived for the most promising leads. A single compound is then selected and evaluated through a series of clinical trials^6^, becoming ready for commercial production and distribution if the compound successfully passes them^7^. Computational methods, including docking protocols and methods to predict structure-activity relationships (SAR), have been integrated into the drug development process, leading to efficiencies in cost and labor and reduction of risk ^8^.

# **Overview of docking methods**

A variety of algorithms and software have been developed to virtually “dock” protein three-dimensional (3D) structures to small molecules^6,9–32^. Such methods output binding poses and binding energies and are evaluated for their accuracy relative to experimentally observed data^5,12,13,17,25,27,28^. Popular docking packages include Autodock Vina^18^, GOLD^19^ and Glide^21^, and many have been used to predict therapeutic hits in virtual high-throughput screening ^5,20,33–36^. While these methods have produced promising results, the use of computational docking methods for drug discovery is still in its early stages ^16,22^. The primary use of docking methods is to predict the three-dimensional pose and binding mode of a single ligand relative to a single protein. Although the understanding of receptor-ligand interactions is made stronger by the predicted structure, the predicted confirmation is less important than its use in predicting binding affinity and is even less important than the ability of the ligand to inhibit the function of the protein. Unfortunately, the factors that contribute to binding affinity and functional inhibition are not considered well by traditional methods^8^.

# **Supporting Methods**

**The CANDO shotgun drug discovery and repurposing platform**

The Computational Analysis for Novel Drug Opportunities (CANDO) platform (<http://protinfo.org/cando>) uses a combination of chem- and bio-informatic methods with hierarchical fragment-based docking with dynamics protocol to generate interaction scores between “all” (currently 3,733) human ingestible compounds (SMILES provided in the GitHub repository) and “all” (currently 48,278) protein structures representative of the protein universe, resulting in compound-proteome interaction signatures that are then compared and ranked by proteomic similarity^37,38^. We used a set of 46,784 experimentally determined and modelled proteins in this study (entire list is provided in the GitHub data repository - *cando_proteins.lst*) and used our bioinformatic docking method^38^ to compute a 3,733 x 46,784 compound-protein interaction matrix (also provided as part of GitHub repository - *all_new.protein_compound_interact_real.fpt*). We used the root mean square deviation (RMSD) metric to calculate the similarities between two proteomic interaction signatures for two compounds to obtain a scalar quantity representing the functional proteomic similarity and subsequently applied this value to repurpose existing drugs for new uses (*canpredict* executable provided on GitHub). The CANDO compound library consists of all human ingestible drugs known to treat an indication (“known drugs”) and all the compounds being investigated for potential medicinal properties, including the selected psychoactive compounds. An all against all similarity is employed to determine the closest signatures between all drugs approved for specific indications and all other compounds in our library, simultaneously predicting repurposable candidates from these drug sets for myriad of indications. We have previously benchmarked the CANDO version 1 (CANDO v1) platform to quantify the prediction/repurposing accuracy to retrieve all known drugs for all 1439 indications/diseases with at least two approved drugs^37,38^ and have applied our platform for the study of polypharmacology^39^. These results highlight the ability of our platform to reproduce known treatments for indications when multiple (two or more) treatments are available using the RMSD metric. We utilized the same RMSD metric to predict putative repurposeable drugs for indications with only one known drug. Since the compound-proteome signatures is only dependent on the similarity of two signatures, the benchmarking RMSD metric is also applicable for mental health indications with only a single known treatment to make predictions.

Here, we use our RMSD metric to determine which psychoactive compounds (structures in Tables S1-S6) have proteomic signatures that match FDA approved drugs used for treating mental health indications. This resulted in a ranked list of the psychoactives and all known drugs for each mental health indication (lowest to highest RMSDs between the compound-proteome interaction signature pairs corresponding to the known drug for the indication and all CANDO compounds). We filtered this list using four cutoffs, resulting in Top10, Top25, Top40, and Top100 predictions. Based on our benchmarking results, higher ranking corresponds to more accurate results.

**Classification of psychoactive compounds via substructure searching**

The classification of psychoactives was performed using the opensource tool, obgrep^40^. For each class, a SMILES string representing the compound class was used to search the compounds. These strings are used to construct a molecular subgraph that is screened for isomorphisms in the compound. If such a subgraph is found the compound is classified as one of the five classes in the order shown below. Once a compound is classified, it is removed from the list as to prevent a compound being reclassified again. A script is provided in the GitHub repository called “make_categories.sh” to perform the classifications mentioned in this work. The SMILES strings, in the order of classification, are given below:

1. 'c1ccc2c(c1)c(c[nH]2)CCN' (Tryptamines)
2. ‘O=C(c1ccccc1)C(N)C’ (Cathinones)
3. ‘c1ccccc1[CH2]C(N)[CH3]’ (Amphetamines)
4. ‘c1ccccc1CCN’ (for remaining phenethylamines)
5. ‘CCCCCc1cc(O)c(C)cc1’ (Cannabinols)

# **Summary**

- Traditional drug discovery is based on a single target (compound, protein, indication) approach. The cost, risk, and failure rate of traditional drug discovery approaches are high because drugs function by interacting with several proteins in a cooperative manner, leading to multiple effects and side effects, and these interactions are not accounted for in the discovery process.
- Development of therapeutics for mental health indications is especially limited by this approach since they are complex disorders caused by the dysfunction of multiple proteins (spanning several different classes and pathways).
- The CANDO platform addresses this issue by virtually screening all known human ingestible compounds with all proteins in a proteome. An all-vs-all compound-proteome signature comparison analysis is conducted, and the similarities between proteomic interaction signatures for compounds with unknown action and the compounds known to treat a given indication is obtained. This comparison results in indication-specific ranked lists of predictions that can be mined for novel drug opportunities. Since this approach is done computationally, it is also able to investigate illicit and otherwise difficult to study compounds.
- The medicinal potential of the compounds as described by Alexander Shulgin has not been previously explored due to illicit nature of many of these compounds. Current legislation allowing for the medicinal use of cannabinoids provides another opportunity for the investigation of such compounds for their medicinal properties and causal behavior. The CANDO platform indicates that the phenylethylamine, tryptamine, and cannabinoid psychoactives have the promise and the potential of providing useful leads for the treatment of a variety of mental health indications, relative to randomized controls.
- Some members of the tryptamine class of psychoactives indicate promise for the treatment of sleep-related disorders such as jet lag and other circadian rhythm disorders. This is a likely result of melatonin (a tryptamine) being the only compound currently used for the treatment of these indications present in CANDO. There is also evidence that these compounds may present useful leads for the treatment of Attention Deficit Disorder with Hyperactivity and Autism.
- The antidepressant bupropion (a cathinone) and the antitussive dextromethorphan (a compound fairly similar to phenethylamine) are predicted for the treatment of cocaine-related disorders. This mirrors the current understanding of these drugs already present in the literature. The ability of the CANDO platform to predict these drugs for the treatment of cocaine-related disorders is an example of the usefulness of this platform for the development of therapeutics for mental health indications.
- Cannabinoids were predicted to have the ability to treat epilepsy by the CANDO platform, among other indications, which has recently been validated by clinical studies.

# **Supporting Figure**


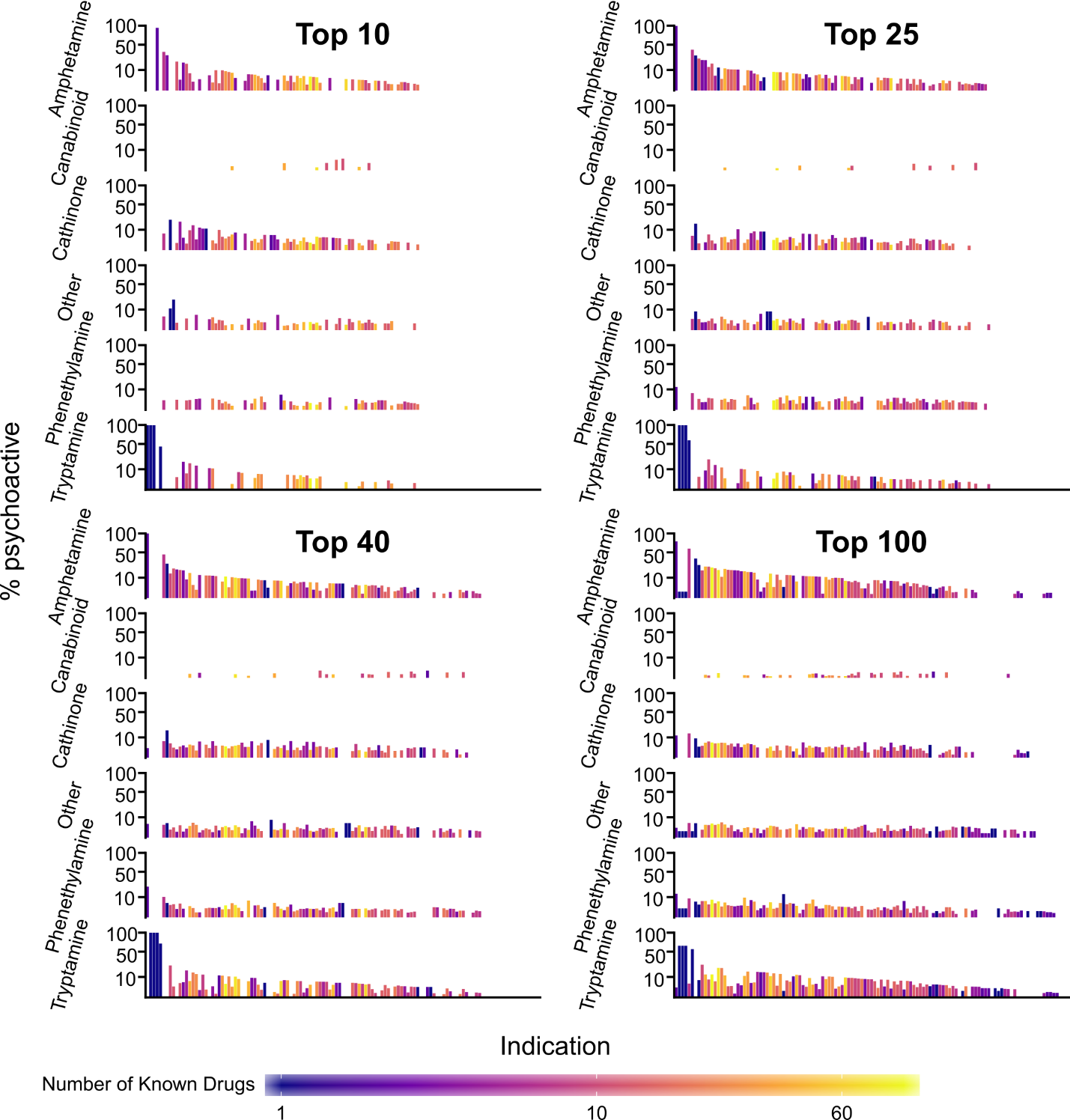


**Figure S1**: Effect of psychoactive class on the normalized indication rank. The color of each indication indicates the number of known drugs to treat the given indication (given as the X-axis). The Y-axis is the normalized indication rank for the indication on the X-axis. Numeric values are given in **Table S9.**

# **Supporting Tables**

Supporting tables showing structures of 428 psychoactives used in our study that are classified by category. The compounds numbering starts at from 1 for each category shown. ChemOffice by PerkinElmer Informatics was used to prepare the structures.

**Table S1:** Structures classified as amphetamine (149 structures).

**Table S2:** Structures classified as cannabinoid (6 structures).

**Table S3:** Structures classified as cathinone (20 structures).

**Table S4:** Structures classified as unclassified (22 structures).

**Table S5:** Structures classified as phenthylamine (122 structures).

**Table S6:** Structures classified as tryptamine (109 structures).

**Table S7**: Indication ranks for mental health indications calculated for all top selections (data used to plot Figure 2).

**Table S8**: Normalized compound rank for all psychoactives calculated for all top sets (data used to plot Figures 3 and 4).

**Table S9**: Relationships between psychoactive classes and specific indications as calculated by the percentage of predictions for a given indication which belong to the given class (data used to plot Figure S1).

**Table S10**: Raw KS p-values

**Table S11**: Raw paired T-test p-values

## **Table S1**: Structures classified as amphetamines (149 structures)

|    \| COc1ccc(CC(C)NCC(O)c2ccc(O)c(NC=O)c2)cc1 \| \| --- \| |    \| CNC(C)Cc1cc(OC)ccc1OC \| \| --- \| |    \| CNC(C)Cc1ccc(OC)cc1 \| \| --- \| |
| --- | --- | --- | --- | --- | --- |
| Formoterol | 2,5-dimethoxy-n-methylamphetamine | 4-methoxy-n-methylamphetamine |
|    \| COc1c2OCOc2cc(CC(C)N)c1OC \| \| --- \| |    \| CNC(C)Cc1cc(OC)c(Br)cc1OC \| \| --- \| |    \| COc1c2OCOc2ccc1CC(C)N \| \| --- \| |
| 2,3-dimethoxy-4,5-methylenedioxyamphetamine | 4-bromo-2,5-dimethoxy-n-methylamphetamine | 2-methoxy-3,4-methylenedioxyamphetamine |
|    \| CC(N)Cc1cccc2OCOc12 \| \| --- \| |    \| CCNC(C)Cc1ccc2OCOc2c1 \| \| --- \| |    \| COc1ccc(CC(C)N)c2OCOc12 \| \| --- \| |
| 2,3-methylenedioxyamphetamine | methylenedioxyethylamphetamine | 4-methoxy-2,3-methylenedioxyamphetamine |
|    \| CCCCc1cc(OC)c(CC(C)N)cc1OC \| \| --- \| |    \| CC(Cc1ccc2OCOc2c1)NO \| \| --- \| |    \| COc1ccc(CC(C)N)cc1 \| \| --- \| |
| 2,5-dimethoxy-4-butylamphetamine | methylenedioxyhydroxyamphetamine | 4-methoxyamphetamine |
|    \| COc1cc(CC(C)N)c(OC)cc1Cl \| \| --- \| |    \| CC(Cc1ccc2OCOc2c1)N(C)O \| \| --- \| |    \| COc1cc(OC)c(OC)cc1CC(C)N \| \| --- \| |
| 2,5-dimethoxy-4-chloroamphetamine | methylenedioxyhydroxymethamphetamine | 2,4,5-trimethoxyamphetamine |
|    \| CCOc1cc(OC)c(CC(C)N)cc1OC \| \| --- \| |    \| CNC(C)Cc1cc2OCOc2cc1OC \| \| --- \| |    \| COc1ccc(CC(C)N)c(OC)c1OC \| \| --- \| |
| 2,5-dimethoxy-4-ethoxyamphetamine | 2-methoxy-n-methyl-4,5-methylenedioxyamphetamine | 2,3,4-trimethoxyamphetamine |
|    \| CCc1cc(OC)c(CC(C)N)cc1OC \| \| --- \| |    \| COc1cc2OCOc2cc1CC(C)N \| \| --- \| |    \| COc1cc(CC(C)N)c(OC)c(OC)c1 \| \| --- \| |
| 2,5-dimethoxy-4-ethylamphetamine | 2-methoxy-4,5-methylenedioxyamphetamine | 2,3,5-trimethoxyamphetamine |
|    \| COc1cc(CC(C)N)c(OC)cc1F \| \| --- \| |    \| COc1cc(CC(C)N)c(SC)cc1OC \| \| --- \| |    \| COc1ccc(OC)c(OC)c1CC(C)N \| \| --- \| |
| 2,5-dimethoxy-4-fluoroamphetamine | 4,5-dimethoxy-2-methylthioamphetamine | 2,3,6-trimethoxyamphetamine |
|    \| CCCc1cc(OC)c(CC(C)N)cc1OC \| \| --- \| |    \| CC(N)Cc1ccc(Br)cc1 \| \| --- \| |    \| COc1cc(OC)c(CC(C)N)c(OC)c1 \| \| --- \| |
| 2,5-dimethoxy-4-propylamphetamine | para-bromoamphetamine | 2,4,6-trimethoxyamphetamine |
|    \| COc1cc(c(OC)cc1CC(C)N)C(F)(F)F \| \| --- \| |    \| CC(N)Cc1ccc(Cl)cc1 \| \| --- \| |    \| COc1cc(CC(C)N)cc(OC)c1OC \| \| --- \| |
| 2,5-dimethoxy-4-trifluoromethylamphetamine | para-chloroamphetamine | 3,4,5-trimethoxyamphetamine |
|    \| CC(N)Cc1cc2OCOc2cc1Br \| \| --- \| |    \| CCOc1ccc(CC(C)N)cc1 \| \| --- \| |    \| COc1cc(C)c(cc1CC(C)N)S(=O)C \| \| --- \| |
| 2-bromo-4,5-methylenedioxyamphetamine | para-ethoxyamphetamine | 2-methoxy-4-methyl-5-methylsulfinylamphetamine |
|    \| CC(N)Cc1ccccc1F \| \| --- \| |    \| CC(N)Cc1ccc(I)cc1 \| \| --- \| |    \| CNC(C)Cc1ccccc1 \| \| --- \| |
| 2-fluoroamphetamine | para-iodoamphetamine | levomethamphetamine |
|    \| CNC(C)Cc1ccccc1F \| \| --- \| |    \| COc1ccc(CC(C)N)cc1 \| \| --- \| |    \| CC(N)Cc1c(c(c(c(c1)OC)OC)OC)OC \| \| --- \| |
| 2-fluoromethamphetamine | para-methoxyamphetamine | 2,3,4,5-Tetramethoxyamphetamine |
|    \| CC(N)Cc1ccc2OCOc2c1C \| \| --- \| |    \| CCNC(C)Cc1ccc(OC)cc1 \| \| --- \| |    \| CC(N)Cc1ccccc1 \| \| --- \| |
| 2-methyl-3,4-methylenedioxyamphetamine | para-methoxyethylamphetamine | amphetamine |
|    \| CC(N)Cc1ccc(Cl)c(Cl)c1 \| \| --- \| |    \| CNC(C)Cc1ccc(OC)cc1 \| \| --- \| |    \| CC(N)Cc1ccccc1 \| \| --- \| |
| 3,4-dichloroamphetamine | para-methoxymethamphetamine | levoamphetamine |
|    \| CC(N)Cc1ccc2OCOc2c1 \| \| --- \| |    \| CNC(C)Cc1ccc(O)cc1 \| \| --- \| |    \| CC(Cc1ccccc1)NC(=O)C(N)CCCCN \| \| --- \| |
| 3,4-methylenedioxyamphetamine | pholedrine | lisdexamfetamine |
|    \| COc1cc(CC(C)N)cc(OC)c1OCc1ccccc1 \| \| --- \| |    \| CCCNC(C)Cc1ccccc1 \| \| --- \| |    \| CC(C)(N)Cc1ccccc1 \| \| --- \| |
| 4-benzyloxy-3,5-dimethoxyamphetamine | propylamphetamine | ortetamine |
|    \| CCOc1c(OC)cc(CC(C)N)cc1OC \| \| --- \| |    \| CC(N)Cc1ccc2CCCCc2c1 \| \| --- \| |    \| CNC(C)Cc1ccccc1OC \| \| --- \| |
| 3,5-dimethoxy-4-ethoxyamphetamine | tetralinylaminopropane | methoxyphenamine |
|    \| CC(N)Cc1cccc(F)c1 \| \| --- \| |    \| CCNC(C)Cc1cccc(SC(F)(F)F)c1 \| \| --- \| |    \| CCCOc1c(OC)cc(CC(C)N)cc1OC \| \| --- \| |
| 3-fluoroamphetamine | tiflorex | 1-(3,5-dimethoxy-4-propoxyphenyl)-2-propanamine |
|    \| COc1cc(CC(C)N)ccc1C \| \| --- \| |    \| COc1ccc(CC(C)N)c(OC)c1 \| \| --- \| |    \| CC(N)Cc1ccc2occc2c1 \| \| --- \| |
| 3-methoxy-4-methylamphetamine | 2,4-dimethoxyamphetamine | 5-(2-aminopropyl)benzofuran |
|    \| COc1cccc(CC(C)N)c1 \| \| --- \| |    \| CSc1c2OCOc2ccc1CC(C)N \| \| --- \| |    \| CC(N)Cc1ccc2OCCc2c1 \| \| --- \| |
| 3-methoxyamphetamine | 3,4-methylenedioxy-2-methylthioamphetamine | 1-(2,3-dihydro-1-benzofuran-5-yl)propan-2-amine |
|    \| CNC(C)Cc1cccc(OC)c1 \| \| --- \| |    \| CCc1cc(SC)c(CC(C)N)cc1OC \| \| --- \| |    \| CC(N)Cc1cc2OCOc2c(C)c1 \| \| --- \| |
| 3-methoxymethamphetamine | 4-ethyl-5-methoxy-2-methylthioamphetamine | 1-(4-methyl-1,3-benzodioxol-6-yl)-2-aminopropane |
|    \| CC(N)Cc1cccc(C)c1 \| \| --- \| |    \| COc1cc(CC(C)N)c(SC)cc1C \| \| --- \| |    \| CC(N)Cc1ccc2ccoc2c1 \| \| --- \| |
| 3-methylamphetamine | 5-methoxy-4-methyl-2-methylthioamphetamine | 1-(1-benzofuran-6-yl)-2-propanamine |
|    \| COc1cc(CC(C)N)cc(OC)c1Br \| \| --- \| |    \| CCc1cc(OC)c(CC(C)N)cc1SC \| \| --- \| |    \| CC(N)Cc1ccc2CCOc2c1 \| \| --- \| |
| 4-bromo-3,5-dimethoxyamphetamine | 4-ethyl-2-methoxy-5-methylthioamphetamine | 1-(2,3-dihydro-1-benzofuran-6-yl)propan-2-amine |
|    \| CCc1ccc(CC(C)N)cc1 \| \| --- \| |    \| COc1cc(C)c(SC)cc1CC(C)N \| \| --- \| |    \| CC(N)Cc1cc2OCOc2cc1C \| \| --- \| |
| 4-ethylamphetamine | 2-methoxy-4-methyl-5-methylthioamphetamine | 1-(6-methyl-1,3-benzodioxol-5-yl)-2-propanamine |
|    \| CC(N)Cc1ccc(F)cc1 \| \| --- \| |    \| CCSc1cc(OC)c(CC(C)N)cc1OC \| \| --- \| |    \| CC(N)Cc1ccc(O)c(O)c1 \| \| --- \| |
| 4-fluoroamphetamine | 2,5-dimethoxy-4-ethylthioamphetamine | alpha-methyldopamine |
|    \| CNC(C)Cc1ccc(F)cc1 \| \| --- \| |    \| COc1cc(SC(C)C)c(OC)cc1CC(C)N \| \| --- \| |    \| CNC(C)Cc1cc(OC)c(C)cc1OC \| \| --- \| |
| 4-fluoromethamphetamine | 2,5-dimethoxy-4-(i)-propylthioamphetamine | beatrice_(psychedelic) |
|    \| CC(N)Cc1ccc(C)cc1 \| \| --- \| |    \| COc1cc(Sc2ccccc2)c(OC)cc1CC(C)N \| \| --- \| |    \| CCOC(=O)NC(C)(C)Cc1ccc(Cl)cc1 \| \| --- \| |
| 4-methylamphetamine | 2,5-dimethoxy-4-phenylthioamphetamine | cloforex |
|    \| CNC(C)Cc1ccc(C)cc1 \| \| --- \| |    \| CCCSc1cc(OC)c(CC(C)N)cc1OC \| \| --- \| |    \| CC(C)(N)Cc1ccccc1Cl \| \| --- \| |
| 4-methylmethamphetamine | 2,5-dimethoxy-4-(n)-propylthioamphetamine | clortermine |
|    \| CSc1ccc(CC(C)N)cc1 \| \| --- \| |    \| CCCCCc1cc(OC)c(CC(C)N)cc1OC \| \| --- \| |    \| COc1cc(CC(C)N)c(OC)c2OCOc12 \| \| --- \| |
| 4-methylthioamphetamine | 2,5-dimethoxy-4-(n)-amylamphetamine | 1-(4,7-dimethoxy-1,3-benzodioxol-5-yl)propan-2-amine |
|    \| COc1cc(SC)c(OC)cc1CC(C)N \| \| --- \| |    \| COc1cc(CC(C)N)c(OC)cc1Br \| \| --- \| |    \| CC(C)(Cc1ccc(Cl)cc1)NCCO \| \| --- \| |
| 4-methylthio-2-5-dimethoxyamphetamine | 2,5-dimethoxy-4-bromoamphetamine | etolorex |
|    \| CC(Cc1ccccc1)/N=C/C(Cl)(Cl)Cl \| \| --- \| |    \| COc1cc(CC(C)N)c(OC)cc1CCF \| \| --- \| |    \| COc1cc2CC(C)Oc2cc1CC(C)N \| \| --- \| |
| amfecloral | 2,5-dimethoxy-4-(2-fluoroethyl)-amphetamine | 6-benzofuranethanamine,_2,3-dihydro-5-methoxy-alpha,2-dimethyl- |
|    \| CCCCCc1ccc(CC(C)NC)cc1 \| \| --- \| |    \| COc1cc(CC(C)N)c(OC)cc1I \| \| --- \| |    \| CC(Cc1ccccc1)N(C)CCNc1nc2n(C)c(=O)n(C)c(=O)c2n1C \| \| --- \| |
| amfepentorex | 2,5-dimethoxy-4-iodoamphetamine | fencamine |
|    \| CC(Cc1ccccc1)NC(C#N)c1ccccc1 \| \| --- \| | \| COc1cc(CC(C)N)c(OC)cc1C \| \| --- \| | \| CNC(C)Cc1ccc2OCOc2c1 \| \| --- \| |
| amphetaminil | 2,5-dimethoxy-4-methylamphetamine | ecstasy |
| \| CC(Cc1cccc(c1)C(F)(F)F)NCCOC(=O)c1ccccc1 \| \| --- \| | \| COc1cc(c(OC)cc1CC(C)N)N(=O)=O \| \| --- \| | \| COc1cc(C)cc(OC)c1CC(C)N \| \| --- \| |
| benfluorex | 2,5-dimethoxy-4-nitroamphetamine | 1-(2,6-dimethoxy-4-methylphenyl)propan-2-amine |
| \| CSc1cc(CC(C)N)c(SC)cc1C \| \| --- \| | \| CC(Cc1ccc2OCOc2c1)N(C)O \| \| --- \| | \| CC(N)Cc1ccc(C)c(C)c1 \| \| --- \| |
| 4-methyl-2,5-bis-(methylthio)amphetamine | n-hydroxy-n-methyl-3,4-methylenedioxyamphetamine | xylopropamine |
| \| CC(N)Cc1cccc(C)c1C \| \| --- \| | \| COc1cc(CC(C)N)c(OC)c2CCCc12 \| \| --- \| | \| COc1ccc(OC)c(CC(C)N)c1 \| \| --- \| |
| dimethylamphetamine | 2,5-dimethoxy-3,4-(trimethylene)amphetamine | (2s)-1-(2,5-dimethoxyphenyl)-2-propanamine |
| \| CNC(C)Cc1ccc2OCCOc2c1 \| \| --- \| | \| COc1cc(CC(C)N)c(OC)c2CCCCc12 \| \| --- \| | \| COc1ccc(CC(C)N)cc1OC \| \| --- \| |
| 3,4-ethylenedioxy-n-methylamphetamine | 2,5-dimethoxy-3,4-(tetramethylene)amphetamine | (2s)-1-(3,4-dimethoxyphenyl)-2-propanamine |
| \| CCNC(C)Cc1ccccc1 \| \| --- \| | \| CNC(C)Cc1cc2OCOc2cc1C \| \| --- \| | \| COc1cc2SCOc2cc1CC(C)N \| \| --- \| |
| ethylamphetamine | 2,n-dimethyl-4,5-methylenedioxyamphetamine | 6-(2-aminopropyl)-5-methoxy-1,3-benzoxathiol |
| \| CC(N)Cc1ccc2OC(C)Oc2c1 \| \| --- \| | \| CC(Cc1ccc2OCOc2c1)NCC=C \| \| --- \| | \| COc1cc2CC(C)Oc2cc1CC(C)N \| \| --- \| |
| ethylidenedioxyamphetamine | 3,4-methylenedioxy-n-_allylamphetamine | 6-(2-aminopropyl)-5-methoxy-2-methyl-2,3-dihydrobenzofuran |
| \| CC(Cc1ccccc1)NCCn1cnc2n(C)c(=O)n(C)c(=O)c12 \| \| --- \| | \| CCCCNC(C)Cc1ccc2OCOc2c1 \| \| --- \| | \| COc1cc(CC(C)N)c(OC)c2C3CCC(C3)c12 \| \| --- \| |
| fenethylline | 3,4-methylenedioxy-n-butylamphetamine | 3,6-dimethoxy-4-(2-aminopropyl)benzonorbornane |
| \| CC(Cc1ccccc1)N(C)Cc1ccco1 \| \| --- \| | \| CC(Cc1ccc2OCOc2c1)NCc1ccccc1 \| \| --- \| | \| COc1cc(CC(C)N)c(OC)c2ccccc12 \| \| --- \| |
| furfenorex | 3,4-methylenedioxy-n-benzylamphetamine | 1,4-dimethoxynaphthyl-2-isopropylamine |
| \| COc1cc(CC(C)N)c(OC)c(C)c1C \| \| --- \| | \| CC(Cc1ccc2OCOc2c1)NCC1CC1 \| \| --- \| | \| CNC(C)(C)Cc1ccc2OCOc2c1 \| \| --- \| |
| 2,5-dimethoxy-3,4-dimethylamphetamine | 3,4-methylenedioxy-n-cyclopropylmethylamphetamine | methylenedioxymephentermine |
| \| CC(N)Cc1cccc(O)c1 \| \| --- \| | \| CC(Cc1ccc2OCOc2c1)N(C)C \| \| --- \| | \| CC(C)(N)Cc1ccc2OCOc2c1 \| \| --- \| |
| gepefrine | 3,4-methylenedioxy-n,n-dimethylamphetamine | 3,4-methylenedioxyphentermine |
| \| COc1cc(CC(C)N(C)C)c(OC)cc1I \| \| --- \| | \| CC(Cc1ccc2OCOc2c1)NCCO \| \| --- \| | \| CC(Cc1ccccc1)N(C)Cc1ccccc1 \| \| --- \| |
| 2,5-dimethoxy-n,n-dimethyl-4-iodoamphetamine | 3,4-methylenedioxy-n-(2-hydroxyethyl)amphetamine | benzphetamine |
| \| CC(N)Cc1ccc2CCCc2c1 \| \| --- \| | \| CC(C)NC(C)Cc1ccc2OCOc2c1 \| \| --- \| | \| CCNC(C)Cc1cccc(c1)C(F)(F)F \| \| --- \| |
| indanylaminopropane | 3,4-methylenedioxy-n-isopropylamphetamine | dexfenfluramine |
| \| CC(C)NC(C)Cc1ccccc1 \| \| --- \| | \| COCCNC(C)Cc1ccc2OCOc2c1 \| \| --- \| | \| CC(Cc1ccccc1)NCCC(c1ccccc1)c1ccccc1 \| \| --- \| |
| isopropylamphetamine | 3,4-methylenedioxy-n-(2-methoxyethyl)amphetamine | prenylamine |
| \| CNC(C)Cc1ccc2OCOc2c1 \| \| --- \| | \| CC(Cc1ccc2OCOc2c1)NO \| \| --- \| | \| O1c2cc(cc(OC)c2OC1)CC(N)C \| \| --- \| |
| ecstasy | 3,4-methylenedioxy-n-hydroxyamphetamine | 3-methoxy-4,5-ethylenedioxyamphetamine |
| \| CONC(C)Cc1ccc2OCOc2c1 \| \| --- \| | \| CC(Cc1ccc2OCOc2c1)NCC#C \| \| --- \| | \| Oc1ccc(cc1)CC(N)C \| \| --- \| |
| 3,4-methylenedioxy-n-methyoxyamphetamine | 3,4-methylenedioxy-n-propargylamphetamine | norpholedrine |
| \| COc1cc(OC)c(CC(C)N)cc1Br \| \| --- \| | \| COc1cc(OC)c(SC)cc1CC(C)N \| \| --- \| |  |
| 5-bromo-2,4-dimethoxyamphetamine | 2,4-dimethoxy-5-methylthioamphetamine | |

## **Table S2**: Structures classified as cannabinoids (6 structures)

| \| CCCCCCCC(C)(C)c1ccc(C2CCCC(O)C2)c(O)c1 \| \| --- \| |
| --- | --- |
| cannabicyclohexanol |
| \| CCCCCc1cc(O)c(C2C=C(C)CCC2C(=C)C)c(O)c1 \| \| --- \| |
| cannabidiol |
| \| CCCCCc1cc(O)c2c(OC(C)(C)c3ccc(C)cc23)c1 \| \| --- \| |
| cannabinol |
| \| CCCCCc1cc(O)c2C3C=C(C)CCC3C(C)(C)Oc2c1 \| \| --- \| |
| tetrahydrocannabinol |
| \| CCCCCCC(C)(C)c1cc(O)c2C3CC(=O)CCC3C(C)(C)Oc2c1 \| \| --- \| |
| nabilone |
| \| CCCCCCC(C)(C)c1cc(O)c2C3CC(CO)CCC3C(C)(C)Oc2c1 \| \| --- \| |
| (6ar,10ar)-9-(hydroxymethyl)-6,6-dimethyl-3-(2-methyloctan-2-yl)-6a,7,8,9,10,10a-hexahydro-6h-benzo[c]chromen-1-ol |

## **Table S3**: Structures classified as cathinone (20 structures)

| \| CCN(CC)C(C)C(=O)c1ccccc1 \| \| --- \| | CC(N(C)C)C(=O)c1ccccc1 | CCC(N1CCCC1)C(=O)c1ccccc1 |
| --- | --- | --- | --- |
| amfepramone | metamfepramone | alpha-pyrrolidinobutiophenone |
| \| CNC(C)C(=O)c1ccc(C)c(C)c1 \| \| --- \| | CNC(C)C(=O)c1ccccc1 | CCCC(N1CCCC1)C(=O)c1ccccc1 |
| 3,4-dimethylmethcathinone | methcathinone | alpha-pyrrolidinopentiophenone |
| \| CNC(C)C(=O)c1ccc(Br)cc1 \| \| --- \| | CNC(C)C(=O)c1ccc(OC)cc1 | CC(N1CCCC1)C(=O)c1ccccc1 |
| 4-bromomethcathinone | methedrone | alpha-pyrrolidinopropiophenone |
| \| CCNC(C)C(=O)c1ccccc1 \| \| --- \| | CNC(C)C(=O)c1ccc2OCOc2c1 | CC(NCc1ccccc1)C(=O)c1ccc(C)cc1 |
| ethcathinone | methylone | benzedrone |
| \| CNC(C)C(=O)c1ccc(F)cc1 \| \| --- \| | \| CCCC(N1CCCC1)C(=O)c1ccc2ccccc2c1 \| \| --- \| | CCC(NC)C(=O)c1ccccc1 |
| flephedrone | naphyrone | buphedrone |
| \| CNC(C)C(=O)c1ccc(C)cc1 \| \| --- \| | \| CCCC(NC)C(=O)c1ccccc1 \| \| --- \| | \| CCCC(N1CCCC1)C(=O)c1ccc2OCOc2c1 \| \| --- \| |
| mephedrone | pentedrone | methylenedioxypyrovalerone |
| \| CC(NC(C)(C)C)C(=O)c1cccc(Cl)c1 \| \| --- \| | \| CCCC(N1CCCC1)C(=O)c1ccc(C)cc1 \| \| --- \| |  |
| bupropion | pyrovalerone |  |

## **Table S4**: Unclassified psychoactives(22 structures)

| \| COc1cc(CC=C)cc(OC)c1OC \| \| --- \| | C(N1CCNCC1)c1ccccc1 | Fc1ccc(cc1)N1CCNCC1 |
| --- | --- | --- | --- |
| elemicin | benzylpiperazine | para-fluorophenylpiperazine |
| \| COc1cc(CC=C)cc2OCOc12 \| \| --- \| | COc1cc2C3CC(CC4CCCCN34)OC(=O)/C=C\c3ccc(O)c(c3)c2cc1OC | COc1ccc(cc1)N1CCNCC1 |
| myristicin | cryogenine | para-methoxyphenylpiperazine |
| \| COC(=O)C1CC(OC(=O)C)C(=O)C2C1(C)CCC1C(=O)OC(CC21C)c1ccoc1 \| \| --- \| | C(N1CCN(Cc2ccccc2)CC1)c1ccccc1 | C1CCN(CC1)C1(CCCCC1)c1ccccc1 |
| Salvinorin a | dibenzylpiperazine | phencyclidine |
| CN1C2CCC1CC(C2)OC(=O)C(CO)c1ccccc1 | Clc1cccc(c1)N1CCNCC1 | FC(F)(F)c1cccc(c1)N1CCNCC1 |
| atropine | meta-chlorophenylpiperazine | trifluoromethylphenylpiperazine |
| \| CNC1(CCCCC1=O)c1ccccc1Cl \| \| --- \| | \| CNC(C)Cc1cccs1 \| \| --- \| | \| CCCCCn1cc(C(=O)c2cccc3ccccc23)c2ccccc12 \| \| --- \| |
| ketamine | methiopropamine | 1-naphthyl(1-pentyl-1h-indol-3-yl)methanone |
| \| CN1C2CC(CC1C1OC21)OC(=O)C(CO)c1ccccc1 \| \| --- \| | \| CN1CCN(Cc2ccccc2)CC1 \| \| --- \| | \| CCCCn1cc(C(=O)c2cccc3ccccc23)c2ccccc12 \| \| --- \| |
| scopolamine | methylbenzylpiperazine | (1-butyl-1h-indol-3-yl)(1-naphthyl)methanon |
| \| COc1cc(CN2CCNCC2)c(OC)cc1Br \| \| --- \| | \| C(N1CCNCC1)c1ccc2OCOc2c1 \| \| --- \| | \| [NH3+]CCc1cc2c(OP(=O)(O)O)cccc2[nH]1 \| \| --- \| |
| 4-bromo-2,5-dimethoxy-1-benzylpiperazine | methylenedioxybenzylpiperazine | norbaeocystin |
| \| COc1ccc2c(c1)[nH]c1c(C)nccc21 \| \| --- \| |  |  |
| harmine |  |  |

## **Table S5**: Structures classified as phenethylamines (122 structures)

| \| CCN(C)C(C)C(O)c1ccccc1 \| \| --- \| | CC(NCCn1cnc2n(C)c(=O)n(C)c(=O)c12)C(O)c1ccccc1 | CCOc1c(OC)cc(CCN)cc1SCC |
| --- | --- | --- | --- |
| etafedrine | cafedrine | 3-thioasymbescaline |
| \| CNC(C)C(O)c1ccc(O)cc1 \| \| --- \| | COc1cc(CCN)cc(OC)c1OCC1CC1 | CCOc1c(OC)cc(CCN)cc1SC |
| oxilofrine | cyclopropylmescaline | 3-thioescaline |
| \| CCOc1cc(OCC)c(OC)cc1CCN \| \| --- \| | COc1cc(CCN)cc(OC)c1C | COc1ccc(CCN)c(OC)c1SC |
| 2,4-diethoxy-5-methoxyamphetamine | 3,5-dimethoxy-4-methylphenethylamine | 3-thiomescaline |
| \| CCOc1cc(OC)c(OCC)cc1CCN \| \| --- \| | CCC(C)Sc1cc(OC)c(CCNO)cc1OC | CCSc1cc(CCN)cc(OC)c1OC |
| 2,5-diethoxy-4-methoxyamphetamine | 2,5-dimethoxy-4-(s)-butylthio-n-hydroxyphenethylamine | 3-thiometaescaline |
| \| CCOc1cc(OC)c(OC)cc1CCN \| \| --- \| | CCSc1cc(OC)c(CCNO)cc1OC | CCOc1cc(CCN)cc(SCC)c1OC |
| 4,5-dimethoxy-2-ethoxyamphetamine | 2,5-dimethoxy-4-ethylthio-n-hydroxyphenethylamine | 3-thiosymbescaline |
| \| CC(N)C(O)c1ccccc1 \| \| --- \| | CCCSc1cc(OC)c(CCNO)cc1OC | CCOc1cc(CCN)cc(SCC)c1OCC |
| cathine | 2,5-dimethoxy-n-hydroxy-4-(n)-propylthiophenethylamine | 3-thiotrescaline |
| \| CC(N)C(O)c1ccc(O)c(O)c1 \| \| --- \| | COc1ccc(CCN)c(OC)c1OC | CCOc1cc(CCN)cc(OC)c1SCC |
| levonordefrin | isomescaline | 4-thioasymbescaline |
| \| C1CCC23CCNC(Cc4ccccc24)C3C1 \| \| --- \| | COc1cc(CCN)cc(OC)c1OC(C)C | COc1c(CCN)ccc(SC)c1OC |
| morphinan | isoproscaline | 4-thioisomescaline |
| \| COc1cc(CCN)c(OC)cc1Br \| \| --- \| | COc1cc2C(CN)CCc2c(OC)c1OC | CCOc1cc(CCN)cc(OC)c1SC |
| nexus | jimscaline | 4-thiometaescaline |
| \| CCC(Cc1ccc2OCOc2c1)NC \| \| --- \| | COc1cc(CCN)cc2OCOc12 | CCOc1cc(CCN)cc(OCC)c1SC |
| eden | lophophine | 4-thiosymbescaline |
| \| CN1CCC23CCCCC2C1Cc1ccc(O)cc31 \| \| --- \| | NC1Cc2cc3OCOc3cc2C1 | CCOc1cc(CCN)cc(OCC)c1SCC |
| dextrorphan | 6,7-dihydro-5h-indeno(5,6-d)-1,3-dioxol-6-amine | 4-thiotrescaline |
| \| COc1ccccc1CNCCc1cc(OC)c(Br)cc1OC \| \| --- \| | COc1cc(CCN)cc(OC)c1OC | CCOc1cc(CCN)cc(SC)c1OCC |
| 2-(4-bromo-2,5-dimethoxyphenyl)-n-(2-methoxybenzyl)ethanamine | mescaline | 5-thioasymbescaline |
| \| COc1cc(CCNCc2ccccc2F)c(OC)cc1I \| \| --- \| | CCOc1cc(CCN)cc(OC)c1OC | CCOc1cc(CCN)cc(SC)c1OC |
| n-(2-fluorobenzyl)-2-(4-iodo-2,5-dimethoxyphenyl)ethanamine | metaescaline | 5-thiometaescaline |
| \| COc1cc(CCNCc2ccccc2O)c(OC)cc1I \| \| --- \| | CCCOc1cc(CCN)cc(OC)c1OC | COc1cc(CCN)cc(OC)c1OCC=C |
| 2-({[2-(4-iodo-2,5-dimethoxyphenyl)ethyl]amino}methyl)phenol | metaproscaline | 4-allyloxy-3,5-dimethoxyphenethylamine |
| \| COc1ccccc1CNCCc1cc(OC)c(I)cc1OC \| \| --- \| | COc1cc(CCN)cc(OC)c1OCC(=C)C | Cc1cc(Br)c(cc1O)C(O)CN |
| 2-(4-iodo-2,5-dimethoxyphenyl)-n-(2-methoxybenzyl)ethanamine | methallylescaline | 2,5-dimethoxy-beta-hydroxy-4-methylphenethylamine |
| \| COc1cc(Br)c(OC)c2CC(CNCc3ccccc3OC)c12 \| \| --- \| | COc1ccc(OC)c(c1)C(O)C(C)N | COc1cc(OCC2CC2)c(OC)cc1CCN |
| n-[[(7r)-3-bromo-2,5-dimethoxy-7-bicyclo[4.2.0]octa-1,3,5-trienyl]methyl]-1-(2-methoxyphenyl)methanamine | methoxamine | 4-cyclopropylmethoxy-3,5-dimethoxyphenethylamine |
| \| COc1cc(CCN)c(OC)cc1Cl \| \| --- \| | CCC(Cc1ccc2OCOc2c1)NC | COc1ccc(CCN)cc1OC |
| 2,5-dimethoxy-4-chlorophenethylamine | methylbenzodioxolylbutanamine | 3,4-dimethoxyphenethylamine |
| \| COc1ccccc1CNCCc1cc(OC)c(Cl)cc1OC \| \| --- \| | CNCCc1ccc2OCOc2c1 | CCOc1c(OC)cc(CCN)cc1OC |
| 2-(4-chloro-2,5-dimethoxyphenyl)-n-(2-methoxybenzyl)ethanamine | methylenedioxymethylphenethylamine | escaline |
| \| COc1cc(CCN)c(OC)cc1F \| \| --- \| | NCCc1ccc2OCOc2c1 | CCC(N)Cc1ccc2OCOc2c1 |
| 2,5-dimethoxy-4-fluorophenethylamine | methylenedioxyphenethylamine | 2-ethylamino-1-(3,4-methylenedioxyphenyl)butane |
| \| COc1cc(CCN)c(OC)c(C)c1C \| \| --- \| | CC(c1ccccc1)C(C)(C)N | CCCC(Cc1ccc2OCOc2c1)NCC |
| 2,5-dimethoxy-3,4-dimethylphenethylamine | pentorex | 2-ethylamino-1-(3,4-methylenedioxyphenyl)pentane |
| \| COc1ccc(OC)c(CCN)c1 \| \| --- \| | COc1cc(CCN)cc(OC)c1OCCc1ccccc1 | CCCC(Cc1ccc2OCOc2c1)NC |
| 2,5-dimethoxyphenethylamine | phenescaline | 2-methylamino-1-(3,4-methylenedioxyphenyl)pentane |
| \| COc1cc(c(OC)cc1CCN)[N](=O)O \| \| --- \| | \| CCC(N)Cc1ccccc1 \| \| --- \| | \| NCCc1ccccc1 \| \| --- \| |
| 2,5-dimethoxy-4-nitrophenethylamine | phenylisobutylamine | phenethylamine |
| \| COc1cc(OC(C)C)c(OC)cc1CCN \| \| --- \| | \| CCCOc1c(OC)cc(CCN)cc1OC \| \| --- \| | \| COc1cc(SC(C)C)cc(OC)c1CCN \| \| --- \| |
| 2,5-dimethoxy-4-(i)-propoxyphenethylamine | proscaline | 2-[4-(isopropylsulfanyl)-2,6-dimethoxyphenyl]ethanamine |
| \| CCCc1cc(OC)c(CCN)cc1OC \| \| --- \| | \| CCOc1cc(CCN)cc(OCC)c1OC \| \| --- \| | \| CCSc1c(OC)cc(CCN)cc1OC \| \| --- \| |
| 2,5-dimethoxy-4-(n)-propylphenethylamine | symbescaline | benzeneethanamine,_4-(ethylthio)-3,5-dimethoxy- |
| \| COc1cc([Se]C)c(OC)cc1CCN \| \| --- \| | \| COc1cc(Br)c(OC)c2CC(CN)c12 \| \| --- \| | \| COc1cc(CCN)cc(OC)c1SC \| \| --- \| |
| 2,5-dimethoxy-4-methylseleneophenethylamine | 1-[(7r)-3-bromo-2,5-dimethoxybicyclo[4.2.0]octa-1,3,5-trien-7-yl]methanamine | benzeneethanamine,_3,5-dimethoxy-4-(methylthio)- |
| \| COCCSc1cc(OC)c(CCN)cc1OC \| \| --- \| | \| CCCCSc1c(OC)cc(CCN)cc1OC \| \| --- \| | \| COc1cc(OC)c(OC)cc1CCN \| \| --- \| |
| 2,5-dimethoxy-4-(2-methoxyethylthio)phenethylamine | thiobuscaline | 2,4,5-trimethoxyphenethylamine |
| \| COc1cc(SC2CC2)c(OC)cc1CCN \| \| --- \| | \| CCCSc1c(OC)cc(CCN)cc1OC \| \| --- \| | \| NCCc1cc(c(c(c1)OC)OCC#C)OC \| \| --- \| |
| 2,5-dimethoxy-4-cyclopropylthiophenethylamine | thioproscaline | 3,5-dimethoxy-4-(2-propynyloxy)phenethylamine |
| \| CCC(C)Sc1cc(OC)c(CCN)cc1OC \| \| --- \| | \| CCOc1cc(CCN)cc(OCC)c1OCC \| \| --- \| | \| COc1ccc2CC3C4CCCCC4(CCN3C)c2c1 \| \| --- \| |
| 2,5-dimethoxy-4-(s)-butylthiophenethylamine | trisescaline | dextromethorphan |
| \| COc1cc(SC(C)C)c(OC)cc1CCN \| \| --- \| | \| COc1cc(CCN)c(OC)cc1C \| \| --- \| | \| C(C(CC)N)c1cc2c(OCO2)cc1 \| \| --- \| |
| 2,5-dimethoxy-4-(i)-propylthiophenethylamine | 2-(2,5-dimethoxy-4-methylphenyl)ethanamine | (2s)-1-(1,3-benzodioxol-5-yl)-2-butanamine |
| \| CCCCSc1cc(OC)c(CCN)cc1OC \| \| --- \| | \| CCc1cc(OC)c(CCN)cc1OC \| \| --- \| | \| NCCc1c(cc(c(c1)OC)SCCF)OC \| \| --- \| |
| 2,5-dimethoxy-4-(t)-butylthiophenethylamine | 2,5-dimethoxy-4-ethylphenethylamine | 2,5-dimethoxy-4-(2-fluoroethylthio)phenethylamine |
| \| COc1ccccc1CNCCc1cc(OC)c(cc1OC)C(F)(F)F \| \| --- \| | \| COc1cc(CCN)c(OC)c2CCCc12 \| \| --- \| | \| NCCc1c(cc(c(c1)OC)[N+](=O)[O-])OC \| \| --- \| |
| 2-[2,5-dimethoxy-4-(trifluoromethyl)phenyl]-n-(2-methoxybenzyl)ethanamine | 2,5-dimethoxy-3,4-(trimethylene)phenethylamine;_5-(2-aminoethyl)-4,7-dimethoxyindane) | 2,5-dimethoxy-4-nitrophenethylamine |
| \| COc1cc(C#C)c(OC)cc1CCN \| \| --- \| | \| COc1cc(CCN)c(OC)c2CCCCc12 \| \| --- \| | \| C(Cc1c(cc(c(c1)OC)CCCC)OC)N \| \| --- \| |
| 1-(4-ethynyl-2,5-dimethoxyphenyl)-2-aminoethane | 2,5-dimethoxy-3,4-(tetramethylene)phenethylamine;_6-(2-aminoethyl)-5,8-dimethoxy-tetralin | 2-(2,5-dimethoxy-4-butylphenyl)ethan-1-amine |
| \| CCOc1ccc(CCN)cc1OC \| \| --- \| | \| COc1cc(CCN)c(OC)c2C3CCC(C3)c12 \| \| --- \| | \| CSc1cc(CCN)cc(OC)c1OC \| \| --- \| |
| 3-methoxy-4-ethoxyphenethylamine | 3,6-dimethoxy-4-(2-aminoethyl)benzonorbornane | 3-thiomescaline |
| \| CCC(N)Cc1ccc(Cl)cc1 \| \| --- \| | \| COc1cc(CCN)c(OC)c2ccccc12 \| \| --- \| | \| COc1cc(CCN)cc(OC)c1OCC(=C)C \| \| --- \| |
| 4-chlorophenylisobutylamine | 1,4-dimethoxynaphthyl-2-ethylamine | methallylescaline |
| \| COc1cc(C2CC2N)c(OC)cc1C \| \| --- \| | \| COc1cc(CCN)c(OC)cc1I \| \| --- \| | \| COc1cc(CCN)cc(OC)c1OCCc1ccccc1 \| \| --- \| |
| 4-methyl-2,5-methoxyphenylcyclopropylamine | 2-(4-iodo-2,5-dimethoxyphenyl)ethanamine | phenescaline |
| \| CCC(N)Cc1ccc(C)cc1 \| \| --- \| | \| COc1cc(SCCF)c(OC)cc1CCN \| \| --- \| | \| CCC(CO)NC(=O)C1CN(C)C2Cc3cn(C)c4cccc(C2=C1)c34 \| \| --- \| |
| 4-methylphenylisobutylamine | 2,5-dimethoxy-4-(2-fluoroethylthio)phenethylamine | methysergide |
| \| CCOc1cc(CCN)cc(OC)c1OCC \| \| --- \| | \| CCCSc1cc(OC)c(CCN)cc1OC \| \| --- \| | \| CCN(CC)C(=O)C1CN(C)C2Cc3cn(C(=O)C)c4cccc(C2=C1)c34 \| \| --- \| |
| asymbescaline | 2,5-dimethoxy-4-n-propylthiophenethylamine | ergoline-8beta-carboxamide,_1-acetyl-9,10-didehydro-n,n-diethyl-6-methyl- |
| \| CCC(N)Cc1ccc2OCOc2c1 \| \| --- \| | \| COc1cc(SCC2CC2)c(OC)cc1CCN \| \| --- \| | \| CCN(CC)C(=O)C1CN(C)C2Cc3cn(C)c4cccc(C2=C1)c34 \| \| --- \| |
| benzodioxolylbutanamine | 2,5-dimethoxy-4-cyclopropylmethylthiophenethylamine | ergoline-8beta-carboxamide,_9,10-didehydro-n,n-diethyl-1,6-dimethyl- |
| \| COC(CN)c1cc(OC)c(Br)cc1OC \| \| --- \| | \| COc1cc(SC)c(OC)cc1CCN \| \| --- \| | \| COc1cc(cc(OC)c1OCC=C)CCN \| \| --- \| |
| beta-methoxy-2c-b;_4-bromo-2,5-beta-trimethoxyphenethylamine | 2,5-dimethoxy-4-methylthiophenethylamine | allylescaline |
| \| CCCCOc1c(OC)cc(CCN)cc1OC \| \| --- \| | \| COc1ccc(CCN)c(SC)c1OC \| \| --- \| | \| C1OC2=C(O1)C=C(C=C2)CCN \| \| --- \| |
| buscaline | 2-thioisomescaline | homopiperonylamine |
| \| CCCc1cc(OC)c(CCN)cc1OC \| \| --- \| | \| CCCCSc1cc(OC)c(CCN)cc1OC \| \| --- \| |  |
| 2,5-dimethoxy-4-(n)-propylphenethylamine | 2,5-dimethoxy-4-(t)-butylthiophenethylamine | |

## **Table S6**: Structures classified as tryptamines (109 structures)

| \| COc1ccc2[nH]c3C(C)NCCc3c2c1 \| \| --- \| | CCCN(CCC)CCc1c[nH]c2ccc(OC)cc12 | CC1CC(C)N1C(=O)C1CN(C)C2Cc3c[nH]c4cccc(C2=C1)c34 |
| --- | --- | --- | --- |
| aldosterone-stimulating_hormone | 1h-indole-3-ethanamine,_5-methoxy-n,n-dipropyl- | lysergic_acid_2,4-dimethylazetidide |
| \| CC(N)Cc1c[nH]c2ccc(O)cc12 \| \| --- \| | COc1ccc2[nH]cc(CCN(C)C(C)C)c2c1 | CCC(C)NC(=O)C1CN(C)C2Cc3c[nH]c4cccc(C2=C1)c34 |
| alpha-methylserotonin | 5-methoxy-n,n-methylisopropyltryptamine | lysergic_acid_2-butyl_amide |
| \| CN1CC(CC2C1Cc1c[nH]c3cccc2c13)C(=O)NC1(C)OC2(O)C3CCCN3C(=O)C(Cc3ccccc3)N2C1=O \| \| --- \| | \| COc1ccc2[nH]cc(CC3CCCN3C)c2c1 \| \| --- \| | \| CCC(CC)NC(=O)C1CN(C)C2Cc3c[nH]c4cccc(C2=C1)c34 \| \| --- \| |
| dihydroergotamine | 5-methoxy-3-((r)-1-methyl-pyrrolidin-2-ylmethyl)-1h-indole | lysergic_acid_3-pentyl_amide |
| \| CCNC(=O)N(CCCN(C)C)C(=O)C1CC2C(Cc3c[nH]c4cccc2c34)N(CC=C)C1 \| \| --- \| | \| CNCCc1c[nH]c2ccc(OC)cc12 \| \| --- \| | \| CCN(CC)C(=O)C1CN(C)C2Cc3c[nH]c4cccc(C2=C1)c34 \| \| --- \| |
| cabergoline | 1h-indole-3-ethanamine,_5-methoxy-n-methyl- | lysergic_acid_diethylamide |
| \| CN1CC(C=C2C1Cc1c[nH]c3cccc2c13)C(=O)NC1(C)OC2(O)C3CCCN3C(=O)C(Cc3ccccc3)N2C1=O \| \| --- \| | \| COc1ccc2[nH]cc(CCN3CCCC3)c2c1 \| \| --- \| | \| CC(O)NC(=O)C1CN(C)C2Cc3c[nH]c4cccc(C2=C1)c34 \| \| --- \| |
| ergotamine | 5-methoxy-3-[2-(pyrrolidin-1-yl)ethyl]-1h-indole | lysergic_acid_hydroxyethylamide |
| \| CC(C)CC1N2C(=O)C(NC(=O)C3CN(C)C4Cc5c(Br)[nH]c6cccc(C4=C3)c56)(OC2(O)C2CCCN2C1=O)C(C)C \| \| --- \| | \| CSc1ccc2[nH]cc(CCN(C)C)c2c1 \| \| --- \| | \| CCN(C)CCc1c[nH]c2ccccc12 \| \| --- \| |
| bromocriptine | n,n-dimethyl-2-[5-(methylsulfanyl)-1h-indol-3-yl]ethanamine | methylethyltryptamine |
| \| CCC(CO)NC(=O)C1CN(C)C2Cc3c[nH]c4cccc(C2=C1)c34 \| \| --- \| | \| COc1ccc2[nH]cc(CCN(C(C)C)C(C)C)c2c1 \| \| --- \| | \| CC(C)N(C)CCc1c[nH]c2ccccc12 \| \| --- \| |
| methylergometrine | 5-methoxy-diisopropyltryptamine | methylisopropyltryptamine |
| \| CCC(C)C1N2C(=O)C(NC(=O)C3CC4C(Cc5c[nH]c6cccc4c56)N(C)C3)(OC2(O)C2CCCN2C1=O)C(C)C \| \| --- \| | \| CC(C)N(C)CCc1c[nH]c2ccc(C)cc12 \| \| --- \| | \| CCNCCc1c[nH]c2ccccc12 \| \| --- \| |
| epicriptine | 5,n-dimethyl-n-isopropyltryptamine | n-ethyltryptamine |
| \| CC(CO)NC(=O)C1CN(C)C2Cc3c[nH]c4cccc(C2=C1)c34 \| \| --- \| | \| CN(C)CCc1c[nH]c2ccc(C)cc12 \| \| --- \| | \| CNCCc1c[nH]c2ccccc12 \| \| --- \| |
| ergometrine | indole,_3-[2-(dimethylamino)ethyl]-5-methyl- | n-methyltryptamine |
| \| CC(N)Cc1c(C)[nH]c2ccccc12 \| \| --- \| | \| C[N+](C)(C)CCc1c[nH]c2cccc(OP(=O)(O)O)c12 \| \| --- \| | \| CN(C)CCc1c[nH]c2cccc(OC(=O)C)c12 \| \| --- \| |
| 2,alpha-dimethyltryptamine | aeruginascin | o-acetylpsilocin |
| \| CCN(CC)C(=O)C1CN(C)C2Cc3c(Br)[nH]c4cccc(C2=C1)c34 \| \| --- \| | \| CCN(CC)C(=O)C1CN(CC=C)C2Cc3c[nH]c4cccc(C2=C1)c34 \| \| --- \| | \| CCN(CC)C(=O)C1CN(CC#C)C2Cc3c[nH]c4cccc(C2=C1)c34 \| \| --- \| |
| 2-bromo-lsd | (8beta)-n,n-diethyl-6-(prop-2-en-1-yl)-9,10-didehydroergoline-8-carboxamide | (8beta)-n,n-diethyl-6-(2-propyn-1-yl)-9,10-didehydroergoline-8-carboxamide |
| \| CCN(CC)CCc1c(C)[nH]c2ccccc12 \| \| --- \| | \| CCC(N)Cc1c[nH]c2ccccc12 \| \| --- \| | \| CCCN1CC(C=C2C1Cc1c[nH]c3cccc2c13)C(=O)N(CC)CC \| \| --- \| |
| n,n-diethyl-2-(2-methyl-1h-indol-3-yl)ethanamine | alpha-ethyltryptamine | (8beta)-n,n-diethyl-6-propyl-9,10-didehydroergoline-8-carboxamide |
| \| CCN(CC)CCc1c[nH]c2cccc(OC(=O)C)c12 \| \| --- \| | \| CC(N)Cc1c[nH]c2ccccc12 \| \| --- \| | \| CCCN(CCc1c[nH]c2ccccc12)C(C)C \| \| --- \| |
| ethacetin | alpha-methyltryptamine | propylisopropyltryptamine |
| \| CC(C)N(CCc1c[nH]c2cccc(OC(=O)C)c12)C(C)C \| \| --- \| | \| CNC(C)Cc1c[nH]c2ccc(OC)cc12 \| \| --- \| | \| CN(C)CCc1c[nH]c2cccc(O)c12 \| \| --- \| |
| [3-[2-(diisopropylamino)ethyl]-1h-indol-4-yl]_acetate_hydrochloride | 1-(5-methoxy-1h-indol-3-yl)-n-methyl-2-propanamine | psilocin |
| \| CC(C)N(C)CCc1c[nH]c2cccc(OC(=O)C)c12 \| \| --- \| | \| CNCCc1c[nH]c2cccc(OP(=O)(O)O)c12 \| \| --- \| | \| C(Cc1c[nH]c2ccccc12)N1CCCC1 \| \| --- \| |
| 3-{2-[isopropyl(methyl)amino]ethyl}-1h-indol-4-yl_acetate | baeocystin | 3-[2-(1-pyrrolidinyl)ethyl]-1h-indole |
| \| CCCCN(CCCC)CCc1c[nH]c2cccc(O)c12 \| \| --- \| | \| CN(C)CCc1c[nH]c2ccc(O)cc12 \| \| --- \| | \| CN(C)CCc1c(C)[nH]c2ccccc12 \| \| --- \| |
| 3-[2-(dibutylamino)ethyl]-1h-indol-4-ol | bufotenin | n,n-dimethyl-2-(2-methyl-1h-indol-3-yl)ethanamine |
| \| CCN(CC)CCc1c[nH]c2cccc(O)c12 \| \| --- \| | \| CCCCN1CC(C=C2C1Cc1c[nH]c3cccc2c13)C(=O)N(CC)CC \| \| --- \| | \| CC(C)N(CCc1c[nH]c2ccc3OCOc3c12)C(C)C \| \| --- \| |
| 3-[2-(diethylamino)ethyl]-1h-indol-4-ol | (8beta)-6-butyl-n,n-diethyl-9,10-didehydroergoline-8-carboxamide | n-[2-(6h-[1,3]dioxolo[4,5-e]indol-8-yl)ethyl]-n-isopropyl-2-propanamine |
| \| CC(C)N(CCc1c[nH]c2cccc(O)c12)C(C)C \| \| --- \| | \| CCN(CC)C(=O)C1CN(C2CC2)C2Cc3c[nH]c4cccc(C2=C1)c34 \| \| --- \| | \| CN(C)CCc1c[nH]c2ccc3OCOc3c12 \| \| --- \| |
| 3-[2-(diisopropylamino)ethyl]-1h-indol-4-ol | (8beta)-6-cyclopropyl-n,n-diethyl-9,10-didehydroergoline-8-carboxamide | 6h-1,3-dioxolo[4,5-e]indole-8-ethanamine,_n,n-dimethyl- |
| \| CCCN(CCC)CCc1c[nH]c2cccc(O)c12 \| \| --- \| | \| C=CCN(CCc1c[nH]c2ccccc12)CC=C \| \| --- \| | \| CCN(C)CCc1c[nH]c2cccc(O)c12 \| \| --- \| |
| 3-[2-(dipropylamino)ethyl]-1h-indol-4-ol | n-allyl-n-[2-(1h-indol-3-yl)ethyl]-2-propen-1-amine | 1h-indol-4-ol,_3-[2-(ethylmethylamino)ethyl]- |
| \| CC(C)N(C)CCc1c[nH]c2cccc(O)c12 \| \| --- \| | \| CN(C)C(=O)C1CN(C)C2Cc3c[nH]c4cccc(C2=C1)c34 \| \| --- \| | \| CC(C)N(CCc1c[nH]c2cc3OCOc3cc12)C(C)C \| \| --- \| |
| 3-{2-[methyl(propan-2-yl)amino]ethyl}-1h-indol-4-ol | (8beta)-n,n,6-trimethyl-9,10-didehydroergoline-8-carboxamide | 5h-1,3-dioxolo[4,5-f]indole-7-ethanamine,_n,n-bis(1-methylethyl)- |
| \| CN1CCCC1Cc1c[nH]c2cccc(O)c12 \| \| --- \| | \| CN1CC(C=C2C1Cc1c[nH]c3cccc2c13)C(=O)N(CC=C)CC=C \| \| --- \| | \| CN(C)CCc1c[nH]c2cc3OCOc3cc12 \| \| --- \| |
| 3-{[(2r)-1-methyl-2-pyrrolidinyl]methyl}-1h-indol-4-ol | diallyllysergamide | 5h-1,3-dioxolo[4,5-f]indole-7-ethanamine,_n,n-dimethyl- |
| \| CCCN(C)CCc1c[nH]c2cccc(O)c12 \| \| --- \| | \| CCCCN(CCCC)CCc1c[nH]c2ccccc12 \| \| --- \| | \| CC(C)N(C)CCc1c[nH]c2cc3OCOc3cc12 \| \| --- \| |
| 3-{2-[methyl(propyl)amino]ethyl}-1h-indol-4-ol | dibutyltryptamine | n-[2-(5h-[1,3]dioxolo[4,5-f]indol-7-yl)ethyl]-n-methyl-2-propanamine |
| \| Oc1cccc2[nH]cc(CCN3CCCC3)c12 \| \| --- \| | \| CCN(CC)CCc1c[nH]c2ccccc12 \| \| --- \| | \| COc1cc2[nH]cc(CCN(C)C(C)C)c2cc1OC \| \| --- \| |
| 3-[2-(pyrrolidin-1-yl)ethyl]-1h-indol-4-ol | diethyltryptamine | n-[2-(5,6-dimethoxy-1h-indol-3-yl)ethyl]-n-methyl-2-propanamine |
| \| COc1ccc2[nH]cc(CCN(C)C)c2c1O \| \| --- \| | \| CC(C)N(CCc1c[nH]c2ccccc12)C(C)C \| \| --- \| | \| CNC(C)Cc1c[nH]c2ccccc12 \| \| --- \| |
| 4-hydroxy-5-methoxydimethyltryptamine | diisopropyltryptamine | (2r)-1-(1h-indol-3-yl)-n-methyl-2-propanamine |
| \| COc1cccc2[nH]cc(CCN(C)C(C)C)c12 \| \| --- \| | \| CN(C)CCc1c[nH]c2ccccc12 \| \| --- \| | \| COc1ccc2c3CCN=C(C)c3[nH]c2c1 \| \| --- \| |
| 4-methoxy-n-methyl-n-isopropyltryptamine | dimethyltryptamine | harmaline |
| \| CCC(N)Cc1c[nH]c2cccc(C)c12 \| \| --- \| | \| CCCN(CCC)CCc1c[nH]c2ccccc12 \| \| --- \| | \| CCCCN(C)CCc1c[nH]c2ccccc12 \| \| --- \| |
| 4-methyl-alpha-ethyltryptamine | dipropyltryptamine | n-[2-(1h-indol-3-yl)ethyl]-n-methyl-1-butanamine |
| \| CC(N)Cc1c[nH]c2cccc(C)c12 \| \| --- \| | \| CN1CC(C=C2C1Cc1c[nH]c3cccc2c13)C(=O)N \| \| --- \| | \| COc1ccc2c3CCNC(C)c3[nH]c2c1 \| \| --- \| |
| 1-(4-methyl-1h-indol-3-yl)propan-2-amine | ergine | tetrahydroharmine |
| \| CC(N)Cc1c[nH]c2ccc(F)cc12 \| \| --- \| | \| CCC(C)(C)C1N2C(=O)C(NC(=O)C3CC4C(Cc5c[nH]c6cccc4c56)N(C)C3)(OC2(O)C2CCCN2C1=O)C(C)C \| \| --- \| | \| NCCc1c[nH]c2ccccc12 \| \| --- \| |
| 1-(5-fluoro-1h-indol-3-yl)propan-2-amine | ergoloid | tryptamine |
| \| CN(C)CCc1c[nH]c2ccc(F)cc12 \| \| --- \| | \| CCN(CC)C(=O)C1CN(CC)C2Cc3c[nH]c4cccc(C2=C1)c34 \| \| --- \| | \| COc1ccc2[nH]cc(CCNC(=O)C)c2c1 \| \| --- \| |
| 5-fluoro-n,n-dimethyltryptamine | (8beta)-n,n,6-triethyl-9,10-didehydroergoline-8-carboxamide | melatonin |
| \| COc1ccc2[nH]c(C)c(CCN(C)C)c2c1 \| \| --- \| | \| CCN(CC)CCc1c[nH]c2cccc(OP(=O)(O)O)c12 \| \| --- \| | \| N(CCc1c[nH]c2ccccc12)(CCCC)CCCC \| \| --- \| |
| 2-(5-methoxy-2-methyl-1h-indol-3-yl)-n,n-dimethylethanamine | ethocybin | n,n-dibutyltryptamine |
| \| CCC(N)Cc1c[nH]c2ccc(OC)cc12 \| \| --- \| | \| CCN(CCc1c[nH]c2ccccc12)C(C)C \| \| --- \| | \| C1(CN(C2Cc3c[nH]c4cccc(C2=C1)c34)C)C(=O)N(CC)CC \| \| --- \| |
| 1-(5-methoxy-1h-indol-3-yl)-2-butanamine | ethylisopropyltryptamine | n,n-diethyl-6-methyl-9,10-didehydroergoline-8-carboxamide |
| \| COc1ccc2[nH]cc(CC(C)N)c2c1 \| \| --- \| | \| CCNC(=O)C1CN(C)C2Cc3c[nH]c4cccc(C2=C1)c34 \| \| --- \| | \| CC(C)N(CCc1c[nH]c2c1cccc2)CC \| \| --- \| |
| 5-methoxy-1h-indole-3-propane-2-amine | n-ethyl-6-methyl-9,10-didehydroergoline-8-carboxamide | n-ethyl-n-[2-(1h-indol-3-yl)ethyl]-2-propanamine |
| \| COc1ccc2[nH]cc(CCN(CC=C)CC=C)c2c1 \| \| --- \| | \| CN1CC(C=C2C1Cc1c[nH]c3cccc2c13)C(=O)N1CCCC1 \| \| --- \| | \| N(CCc1c[nH]c2ccccc12)(CC)CC \| \| --- \| |
| n-allyl-n-[2-(5-methoxy-1h-indol-3-yl)ethyl]-2-propen-1-amine | [(8beta)-6-methyl-9,10-didehydroergolin-8-yl](pyrrolidin-1-yl)methanone | n,n-diethyltryptamine |
| \| CCN(CC)CCc1c[nH]c2ccc(OC)cc12 \| \| --- \| | \| CN1CC(C=C2C1Cc1c[nH]c3cccc2c13)C(=O)N1CCCCC1 \| \| --- \| | \| CC(C)CC1N2C(=O)C(OC2(O)C2CCCN2C1=O)(/N=C(\O)/C1CC2C(Cc3c[nH]c4cccc2c34)N(C)C1)C(C)C \| \| --- \| |
| 5-methoxy-n,n-diethyltryptamine | (6-methyl-9,10-didehydroergolin-8-yl)(1-piperidinyl)methanone | dihydroergocryptine |
| \| COc1ccc2[nH]cc(CCN(C)C)c2c1 \| \| --- \| | \| CN1CC(C=C2C1Cc1c[nH]c3cccc2c13)C(=O)N1CCOCC1 \| \| --- \| | \| CNC(C)Cc1c[nH]c2ccc(OC)cc12 \| \| --- \| |
| 5-methoxydimethyltryptamine | [(8beta)-6-methyl-9,10-didehydroergolin-8-yl](4-morpholinyl)methanone | 1-(5-methoxy-1h-indol-3-yl)-n-methyl-2-propanamine |
| \| CC(C)N(C)CCc1c[nH]c2cccc(O)c12 \| \| --- \| | \| CCN(CC)C(=O)C1CN(CC#C)C2Cc3c[nH]c4cccc(C2=C1)c34 \| \| --- \| | \| CCCN1CC(C=C2C1Cc1c[nH]c3cccc2c13)C(=O)N(CC)CC \| \| --- \| |
| 3-{2-[methyl(propan-2-yl)amino]ethyl}-1h-indol-4-ol | (8beta)-n,n-diethyl-6-(2-propyn-1-yl)-9,10-didehydroergoline-8-carboxamide | (8beta)-n,n-diethyl-6-propyl-9,10-didehydroergoline-8-carboxamide |
| \| C(Cc1c([nH]c2c1cc(cc2)OC)C)N(C)C \| \| --- \| |  |  |
| 2-(5-methoxy-2-methyl-1h-indol-3-yl)-n,n-dimethylethanamine | | |

## **Table S7:** Indication ranks for mental health indications calculated for all top selections.

| **MESH** | **Indication** | **Drug**  **count** | **Top10** | **Top25** | **Top40** | **Top100** |
| --- | --- | --- | --- | --- | --- | --- |
| **D016574** | Seasonal Affective Disorder | 1 | 100.00 | 100.00 | 100.00 | 67.68 |
| **D020178** | Sleep Disorders, Circadian Rhythm | 1 | 100.00 | 100.00 | 100.00 | 67.68 |
| **D020179** | Jet Lag Syndrome | 1 | 100.00 | 100.00 | 100.00 | 67.68 |
| **D056912** | Binge-Eating Disorder | 2 | 83.33 | 75.00 | 73.08 | 68.18 |
| **D001039** | Aphasia, Broca | 1 | 44.44 | 58.33 | 69.23 | 56.57 |
| **D009290** | Narcolepsy | 7 | 40.74 | 38.89 | 42.31 | 44.11 |
| **D020186** | Sleep Bruxism | 3 | 40.74 | 33.33 | 28.21 | 24.24 |
| **D001308** | Auditory Perceptual Disorders | 1 | 33.33 | 54.17 | 56.41 | 57.58 |
| **D012148** | Restless Legs Syndrome | 10 | 33.33 | 39.58 | 40.00 | 35.96 |
| **D000856** | Anorexia Nervosa | 3 | 29.63 | 25.00 | 23.93 | 25.93 |
| **D002385** | Cataplexy | 12 | 25.93 | 23.61 | 24.57 | 25.17 |
| **D004831** | Epilepsies, Myoclonic | 12 | 25.56 | 25.83 | 23.85 | 21.72 |
| **D013981** | Tic Disorders | 5 | 25.00 | 16.67 | 17.31 | 16.16 |
| **D010842** | Pica | 4 | 22.22 | 22.92 | 22.44 | 22.73 |
| **D020922** | Sleep-Wake Transition Disorders | 1 | 22.22 | 8.33 | 5.13 | 4.04 |
| **D019958** | Attention Deficit and Disruptive Behavior Disorders | 5 | 19.44 | 21.88 | 30.13 | 29.04 |
| **D001289** | Attention Deficit Disorder with Hyperactivity | 22 | 17.28 | 16.67 | 17.66 | 19.14 |
| **D052018** | Bulimia Nervosa | 4 | 16.67 | 17.71 | 21.15 | 25.00 |
| **D019263** | Dysthymic Disorder | 6 | 16.67 | 15.28 | 14.10 | 16.16 |
| **D007172** | Erectile Dysfunction | 26 | 16.34 | 14.46 | 15.69 | 16.64 |
| **D009771** | Obsessive-Compulsive Disorder | 13 | 16.16 | 15.15 | 15.62 | 19.93 |
| **D019964** | Mood Disorders | 14 | 14.44 | 12.92 | 12.56 | 17.27 |
| **D000647** | Amnesia | 44 | 14.38 | 16.05 | 17.72 | 19.55 |
| **D001321** | Autistic Disorder | 32 | 12.26 | 13.65 | 15.47 | 17.55 |
| **D010554** | Personality Disorders | 30 | 11.56 | 13.50 | 13.64 | 14.75 |
| **D019973** | Alcohol-Related Disorders | 3 | 11.11 | 20.83 | 23.08 | 17.68 |
| **D002658** | Developmental Disabilities | 4 | 11.11 | 13.54 | 16.03 | 21.21 |
| **D003072** | Cognition Disorders | 26 | 11.11 | 13.29 | 14.04 | 15.34 |
| **D000379** | Agoraphobia | 5 | 11.11 | 12.50 | 12.31 | 14.14 |
| **D020774** | Pick Disease of the Brain | 1 | 11.11 | 12.50 | 10.26 | 11.11 |
| **D001068** | Eating Disorders | 3 | 11.11 | 6.94 | 10.26 | 15.49 |
| **D003865** | Depressive Disorder, Major | 43 | 10.84 | 13.72 | 14.95 | 16.21 |
| **D007859** | Learning Disorders | 26 | 10.58 | 13.10 | 14.41 | 14.57 |
| **D001008** | Anxiety Disorders | 46 | 10.56 | 12.19 | 14.10 | 15.73 |
| **D019969** | Amphetamine-Related Disorders | 27 | 10.26 | 10.58 | 10.85 | 11.97 |
| **D019955** | Conduct Disorder | 15 | 10.19 | 10.42 | 11.97 | 12.96 |
| **D019970** | Cocaine-Related Disorders | 75 | 10.14 | 13.16 | 13.71 | 14.27 |
| **D016584** | Panic Disorder | 19 | 9.80 | 9.56 | 11.16 | 16.22 |
| **D003693** | Delirium | 8 | 9.72 | 13.02 | 16.35 | 17.80 |
| **D012640** | Seizures | 94 | 8.58 | 9.76 | 10.91 | 12.71 |
| **D017029** | Epilepsy, Complex Partial | 14 | 8.33 | 10.07 | 9.62 | 11.62 |
| **D002659** | Child Development Disorders, Pervasive | 5 | 8.33 | 7.29 | 12.82 | 15.40 |
| **D004827** | Epilepsy | 38 | 7.94 | 9.05 | 9.52 | 10.62 |
| **D001714** | Bipolar Disorder | 42 | 7.94 | 7.74 | 8.21 | 10.42 |
| **D000341** | Affective Disorders, Psychotic | 9 | 7.94 | 7.74 | 8.06 | 8.08 |
| **D019956** | Stereotypic Movement Disorder | 16 | 7.64 | 9.11 | 8.81 | 10.16 |
| **D019966** | Substance-Related Disorders | 11 | 7.41 | 6.48 | 7.98 | 8.98 |
| **D019305** | Epilepsy, Rolandic | 4 | 7.41 | 4.17 | 5.13 | 6.40 |
| **D013226** | Status Epilepticus | 29 | 7.25 | 8.15 | 8.47 | 9.27 |
| **D003866** | Depressive Disorder | 65 | 6.97 | 7.35 | 8.55 | 10.58 |
| **D006556** | Heroin Dependence | 11 | 6.67 | 11.67 | 12.05 | 12.53 |
| **D013375** | Substance Withdrawal Syndrome | 48 | 6.61 | 8.63 | 9.52 | 11.52 |
| **D000430** | Alcohol Withdrawal Delirium | 8 | 6.35 | 8.33 | 10.99 | 9.38 |
| **D005879** | Tourette Syndrome | 15 | 5.93 | 9.17 | 9.57 | 13.13 |
| **D020190** | Myoclonic Epilepsy, Juvenile | 3 | 5.56 | 12.50 | 15.38 | 30.30 |
| **D015140** | Dementia, Vascular | 2 | 5.56 | 10.42 | 15.38 | 28.28 |
| **D013064** | Speech Disorders | 2 | 5.56 | 10.42 | 15.38 | 18.69 |
| **D004414** | Dyspareunia | 2 | 5.56 | 10.42 | 6.41 | 19.19 |
| **D000544** | Alzheimer Disease | 32 | 5.56 | 9.46 | 12.43 | 15.31 |
| **D014029** | Tobacco Use Disorder | 10 | 5.56 | 7.29 | 7.69 | 13.76 |
| **D003704** | Dementia | 13 | 5.56 | 5.83 | 7.18 | 12.53 |
| **D007319** | Sleep Initiation and Maintenance Disorders | 27 | 5.09 | 8.16 | 10.47 | 9.05 |
| **D002653** | Child Behavior Disorders | 14 | 5.05 | 4.55 | 7.23 | 9.46 |
| **D009293** | Opioid-Related Disorders | 10 | 4.94 | 9.26 | 11.97 | 14.14 |
| **D003244** | Consciousness Disorders | 9 | 4.94 | 7.87 | 14.25 | 16.84 |
| **D004830** | Epilepsy, Tonic-Clonic | 20 | 4.86 | 5.73 | 5.61 | 6.57 |
| **D004832** | Epilepsy, Absence | 12 | 4.63 | 6.94 | 7.91 | 10.94 |
| **D011618** | Psychotic Disorders | 47 | 4.50 | 4.62 | 6.58 | 8.54 |
| **D011605** | Psychoses, Substance-Induced | 14 | 4.44 | 8.75 | 11.54 | 14.14 |
| **D012559** | Schizophrenia | 65 | 4.00 | 4.75 | 5.33 | 6.26 |
| **D010698** | Phobic Disorders | 7 | 3.70 | 5.56 | 5.13 | 4.71 |
| **D004833** | Epilepsy, Temporal Lobe | 11 | 3.70 | 4.63 | 4.84 | 7.86 |
| **D000435** | Alcoholic Intoxication | 3 | 3.70 | 4.17 | 2.56 | 3.37 |
| **D012893** | Sleep Disorders | 14 | 3.42 | 3.85 | 6.11 | 8.00 |
| **D003294** | Seizures, Febrile | 8 | 3.17 | 1.79 | 2.20 | 4.47 |
| **D004829** | Epilepsy, Generalized | 7 | 3.17 | 1.79 | 2.20 | 3.03 |
| **D020018** | Sexual Dysfunctions, Psychological | 11 | 3.03 | 4.17 | 3.73 | 7.35 |
| **D020324** | Amnesia, Anterograde | 4 | 2.78 | 10.42 | 16.03 | 19.44 |
| **D013313** | Stress Disorders, Post-Traumatic | 21 | 2.78 | 4.17 | 5.77 | 7.89 |
| **D006970** | Disorders of Excessive Somnolence | 5 | 2.22 | 6.67 | 8.72 | 12.32 |
| **D004828** | Epilepsies, Partial | 19 | 2.08 | 3.39 | 3.21 | 5.30 |
| **D012563** | Schizophrenia, Paranoid | 22 | 1.75 | 1.75 | 2.43 | 3.72 |
| **D000437** | Alcoholism | 17 | 1.48 | 3.89 | 4.27 | 5.59 |
| **D006816** | Huntington Disease | 10 | 1.11 | 4.58 | 6.67 | 12.63 |
| **D005715** | Gambling | 1 | 0.00 | 12.50 | 15.38 | 40.40 |
| **D009497** | Neurotic Disorders | 3 | 0.00 | 8.33 | 14.53 | 15.49 |
| **D012734** | Disorders of Sex Development | 1 | 0.00 | 8.33 | 5.13 | 2.02 |
| **D015161** | Dementia, Multi-Infarct | 1 | 0.00 | 4.17 | 7.69 | 10.10 |
| **D019052** | Depression, Postpartum | 1 | 0.00 | 4.17 | 7.69 | 4.04 |
| **D020195** | Epilepsy, Reflex | 2 | 0.00 | 4.17 | 2.56 | 3.03 |
| **D009021** | Morphine Dependence | 5 | 0.00 | 3.13 | 1.92 | 2.27 |
| **D006998** | Hypochondriasis | 2 | 0.00 | 2.08 | 3.85 | 6.06 |
| **D013036** | Spasms, Infantile | 8 | 0.00 | 1.79 | 1.47 | 1.30 |
| **D007174** | Impulse Control Disorders | 8 | 0.00 | 1.39 | 5.56 | 8.92 |
| **D004775** | Enuresis | 7 | 0.00 | 1.39 | 1.28 | 5.72 |
| **D053206** | Nocturnal Enuresis | 4 | 0.00 | 1.04 | 5.13 | 8.08 |
| **D020270** | Alcohol Withdrawal Seizures | 4 | 0.00 | 1.04 | 2.56 | 6.57 |
| **D019957** | Motor Skills Disorders | 1 | 0.00 | 0.00 | 10.26 | 17.17 |
| **D001883** | Borderline Personality Disorder | 4 | 0.00 | 0.00 | 2.56 | 3.03 |
| **D011604** | Psychoses, Alcoholic | 1 | 0.00 | 0.00 | 2.56 | 2.02 |
| **D014256** | Trichotillomania | 1 | 0.00 | 0.00 | 2.56 | 2.02 |
| **D012560** | Schizophrenia, Catatonic | 5 | 0.00 | 0.00 | 1.92 | 2.53 |
| **D020187** | REM Sleep Behavior Disorder | 2 | 0.00 | 0.00 | 1.28 | 2.53 |
| **D005329** | Fetishism (Psychiatric) | 1 | 0.00 | 0.00 | 0.00 | 3.03 |
| **D010262** | Paraphilias | 3 | 0.00 | 0.00 | 0.00 | 2.53 |
| **D012561** | Schizophrenia, Childhood | 2 | 0.00 | 0.00 | 0.00 | 2.53 |
| **D020961** | Lewy Body Disease | 2 | 0.00 | 0.00 | 0.00 | 2.53 |
| **D012562** | Schizophrenia, Disorganized | 2 | 0.00 | 0.00 | 0.00 | 2.02 |
| **D008607** | Intellectual Disability | 2 | 0.00 | 0.00 | 0.00 | 1.52 |
| **D020191** | Myoclonic Epilepsies, Progressive | 3 | 0.00 | 0.00 | 0.00 | 1.52 |
| **D020817** | Asperger Syndrome | 2 | 0.00 | 0.00 | 0.00 | 1.52 |
| **D019967** | Schizophrenia and Disorders with Psychotic Features | 4 | 0.00 | 0.00 | 0.00 | 1.35 |
| **D003130** | Combat Disorders | 1 | 0.00 | 0.00 | 0.00 | 1.01 |
| **D012569** | Schizotypal Personality Disorder | 2 | 0.00 | 0.00 | 0.00 | 1.01 |
| **D012892** | Sleep Deprivation | 2 | 0.00 | 0.00 | 0.00 | 1.01 |
| **D014899** | Wernicke Encephalopathy | 1 | 0.00 | 0.00 | 0.00 | 1.01 |
| **D057180** | Frontotemporal Dementia | 2 | 0.00 | 0.00 | 0.00 | 1.01 |
| **D009357** | Neonatal Abstinence Syndrome | 2 | 0.00 | 0.00 | 0.00 | 0.51 |

## **Table S8:** Normalized compound rank for all psychoactives calculated for all top sets.

| **Compound** | **Category** | **Top**  **10** | **Top**  **25** | **Top**  **40** | **Top**  **100** |
| --- | --- | --- | --- | --- | --- |
| **2-ethylamino-1-(3,4-methylenedioxyphenyl)pentane** | Phenethylamine | 100. | 100 | 13.3 | 14.7 |
| **3,4-methylenedioxy-n-isopropylamphetamine** | Amphetamine | 100. | 100 | 6.8 | 9.0 |
| **3,4-methylenedioxy-n-propargylamphetamine** | Amphetamine | 100. | 100 | 3.7 | 7.1 |
| **pentedrone** | Cathinone | 100. | 57.1 | 21.9 | 24.4 |
| **cloforex** | Amphetamine | 100. | 38.1 | 29.6 | 14.9 |
| **methylenedioxyhydroxymethamphetamine** | Amphetamine | 100. | 33.3 | 7.9 | 11.9 |
| **methiopropamine** | Other | 100. | 23.4 | 24.2 | 20.7 |
| **4-methylthioamphetamine** | Amphetamine | 100. | 23.0 | 25.1 | 26.8 |
| **3,4-methylenedioxy-n-_allylamphetamine** | Amphetamine | 100. | 9.1 | 9.1 | 13.1 |
| **n-hydroxy-n-methyl-3,4-methylenedioxyamphetamine** | Amphetamine | 100 | 6.7 | 6.7 | 4.1 |
| **buphedrone** | Cathinone | 84.6 | 23.3 | 24.4 | 25.3 |
| **methoxyphenamine** | Amphetamine | 75.0 | 8.9 | 26.8 | 35.2 |
| **scopolamine** | Other | 66.7 | 19.0 | 9.5 | 10.4 |
| **isopropylamphetamine** | Amphetamine | 62.9 | 40.0 | 34.8 | 28.2 |
| **2-fluoromethamphetamine** | Amphetamine | 57.1 | 30.9 | 25.3 | 27.2 |
| **pyrovalerone** | Cathinone | 53.8 | 40.2 | 34.9 | 25.1 |
| **alpha-pyrrolidinopentiophenone** | Cathinone | 50.0 | 39.5 | 28.7 | 21.7 |
| **n-[2-(1h-indol-3-yl)ethyl]-n-methyl-1-butanamine** | Tryptamine | 50.0 | 12.3 | 10.9 | 10.6 |
| **methylethyltryptamine** | Tryptamine | 50.0 | 9.5 | 11.3 | 10.2 |
| **4-methylamphetamine** | Amphetamine | 45.5 | 30.2 | 21.0 | 25.6 |
| **4-fluoroamphetamine** | Amphetamine | 44.7 | 33.0 | 23.5 | 26.6 |
| **bupropion** | Cathinone | 44.2 | 32.6 | 34.1 | 34.7 |
| **6-(2-aminopropyl)-5-methoxy-2-methyl-2,3-dihydrobenzofuran** | Amphetamine | 43.8 | 35.7 | 31.5 | 27.7 |
| **para-chloroamphetamine** | Amphetamine | 41.9 | 28.1 | 20.4 | 22.7 |
| **n-allyl-n-[2-(1h-indol-3-yl)ethyl]-2-propen-1-amine** | Tryptamine | 40.0 | 27.8 | 10.3 | 8.1 |
| **dipropyltryptamine** | Tryptamine | 40.0 | 13.3 | 10.3 | 8.9 |
| **n-allyl-n-[2-(5-methoxy-1h-indol-3-yl)ethyl]-2-propen-1-amine** | Tryptamine | 40.0 | 11.2 | 10.1 | 9.7 |
| **dimethylamphetamine** | Amphetamine | 38.7 | 25.3 | 22.9 | 23.1 |
| **meta-chlorophenylpiperazine** | Other | 36.7 | 35.1 | 36.7 | 42.1 |
| **3,6-dimethoxy-4-(2-aminopropyl)benzonorbornane** | Amphetamine | 35.7 | 35.6 | 31.2 | 21.1 |
| **ketamine** | Other | 34.9 | 29.2 | 30.6 | 27.1 |
| **3-methylamphetamine** | Amphetamine | 34.0 | 28.0 | 19.0 | 24.4 |
| **para-fluorophenylpiperazine** | Other | 33.3 | 37.3 | 36.5 | 29.7 |
| **2-fluoroamphetamine** | Amphetamine | 33.3 | 29.7 | 23.4 | 21.1 |
| **ortetamine** | Amphetamine | 33.3 | 26.0 | 34.2 | 25.4 |
| **lysergic_acid_diethylamide** | Tryptamine | 33.3 | 16.9 | 10.3 | 7.4 |
| **1h-indole-3-ethanamine,_5-methoxy-n,n-dipropyl-** | Tryptamine | 33.3 | 9.8 | 9.9 | 9.5 |
| **metamfepramone** | Cathinone | 32.1 | 29.7 | 32.0 | 25.2 |
| **gepefrine** | Amphetamine | 31.4 | 28.6 | 23.6 | 23.3 |
| **4-fluoromethamphetamine** | Amphetamine | 31.3 | 23.6 | 20.9 | 23.9 |
| **3-fluoroamphetamine** | Amphetamine | 30.2 | 28.0 | 20.7 | 24.4 |
| **benzylpiperazine** | Other | 30.0 | 27.1 | 29.5 | 28.1 |
| **dextromethorphan** | Phenethylamine | 29.8 | 29.3 | 20.8 | 15.2 |
| **phenethylamine** | Phenethylamine | 29.6 | 37.5 | 30.5 | 28.9 |
| **n-ethyl-6-methyl-9,10-didehydroergoline-8-carboxamide** | Tryptamine | 29.6 | 21.4 | 22.0 | 22.6 |
| **3-methoxy-4,5-ethylenedioxyamphetamine** | Amphetamine | 28.6 | 28.6 | 5.3 | 4.1 |
| **n-[2-(6h-[1,3]dioxolo[4,5-e]indol-8-yl)ethyl]-n-isopropyl-2-propanamine** | Tryptamine | 28.6 | 7.4 | 5.7 | 7.4 |
| **ergoline-8beta-carboxamide,_1-acetyl-9,10-didehydro-n,n-diethyl-6-methyl-** | Phenethylamine | 28.6 | 7.0 | 7.6 | 6.7 |
| **4-methylmethamphetamine** | Amphetamine | 28.2 | 23.6 | 20.9 | 23.9 |
| **ethylamphetamine** | Amphetamine | 28.0 | 26.4 | 23.0 | 25.8 |
| **cannabinol** | Cannabinoid | 27.6 | 30.0 | 17.3 | 13.3 |
| **3,4-dimethylmethcathinone** | Cathinone | 26.7 | 23.6 | 24.2 | 25.0 |
| **methcathinone** | Cathinone | 26.5 | 15.9 | 23.4 | 25.6 |
| **para-bromoamphetamine** | Amphetamine | 25.0 | 26.3 | 30.9 | 26.5 |
| **clortermine** | Amphetamine | 25.0 | 25.0 | 39.7 | 27.6 |
| **3-methoxyamphetamine** | Amphetamine | 25.0 | 25.0 | 12.3 | 22.8 |
| **3-methoxy-4-methylamphetamine** | Amphetamine | 25.0 | 25.0 | 4.7 | 20.3 |
| **(8beta)-n,n-diethyl-6-propyl-9,10-didehydroergoline-8-carboxamide** | Tryptamine | 25.0 | 23.8 | 22.0 | 19.3 |
| **5-methoxy-3-((r)-1-methyl-pyrrolidin-2-ylmethyl)-1h-indole** | Tryptamine | 25.0 | 7.7 | 7.4 | 12.1 |
| **amfepramone** | Cathinone | 23.7 | 17.8 | 23.6 | 29.0 |
| **amphetamine** | Amphetamine | 23.7 | 21.7 | 27.4 | 25.9 |
| **4-methyl-2,5-bis-(methylthio)amphetamine** | Amphetamine | 23.5 | 45.5 | 30.5 | 30.1 |
| **alpha-pyrrolidinopropiophenone** | Cathinone | 23.2 | 19.6 | 21.1 | 21.3 |
| **3-[2-(dipropylamino)ethyl]-1h-indol-4-ol** | Tryptamine | 23.1 | 10.6 | 10.1 | 8.3 |
| **(8beta)-n,n,6-trimethyl-9,10-didehydroergoline-8-carboxamide** | Tryptamine | 21.1 | 18.2 | 18.0 | 15.0 |
| **ethcathinone** | Cathinone | 20.7 | 18.8 | 24.5 | 27.0 |
| **(2r)-1-(1h-indol-3-yl)-n-methyl-2-propanamine** | Tryptamine | 20.0 | 45.0 | 35.5 | 26.1 |
| **tryptamine** | Tryptamine | 20.0 | 25.0 | 27.3 | 37.5 |
| **alpha-pyrrolidinobutiophenone** | Cathinone | 20.0 | 24.7 | 24.0 | 25.2 |
| **methylbenzylpiperazine** | Other | 20.0 | 23.9 | 23.5 | 30.4 |
| **tetrahydrocannabinol** | Cannabinoid | 20.0 | 22.2 | 29.0 | 23.9 |
| **alpha-methyltryptamine** | Tryptamine | 20.0 | 20.0 | 47.4 | 32.1 |
| **2,alpha-dimethyltryptamine** | Tryptamine | 20.0 | 20.0 | 42.9 | 37.3 |
| **n-methyltryptamine** | Tryptamine | 20.0 | 20.0 | 32.1 | 30.4 |
| **nabilone** | Cannabinoid | 20.0 | 20.0 | 29.6 | 13.8 |
| **etolorex** | Amphetamine | 20.0 | 20.0 | 4.8 | 16.9 |
| **(8beta)-n,n-diethyl-6-propyl-9,10-didehydroergoline-8-carboxamide** | Tryptamine | 20.0 | 12.7 | 15.4 | 17.8 |
| **dimethyltryptamine** | Tryptamine | 20.0 | 11.2 | 11.3 | 14.0 |
| **1-(5-fluoro-1h-indol-3-yl)propan-2-amine** | Tryptamine | 20.0 | 7.1 | 36.4 | 27.3 |
| **mephedrone** | Cathinone | 18.6 | 18.0 | 28.8 | 25.9 |
| **flephedrone** | Cathinone | 18.6 | 12.7 | 19.1 | 28.6 |
| **amfecloral** | Amphetamine | 18.4 | 33.7 | 30.4 | 31.6 |
| **(8beta)-6-butyl-n,n-diethyl-9,10-didehydroergoline-8-carboxamide** | Tryptamine | 18.2 | 23.3 | 22.9 | 23.3 |
| **ergine** | Tryptamine | 18.2 | 21.5 | 18.3 | 17.4 |
| **n,n-diethyl-6-methyl-9,10-didehydroergoline-8-carboxamide** | Tryptamine | 18.2 | 20.0 | 20.0 | 20.0 |
| **(8beta)-n,n-diethyl-6-(prop-2-en-1-yl)-9,10-didehydroergoline-8-carboxamide** | Tryptamine | 16.7 | 22.2 | 24.2 | 17.7 |
| **lysergic_acid_3-pentyl_amide** | Tryptamine | 16.7 | 19.7 | 19.3 | 21.8 |
| **4-methylphenylisobutylamine** | Phenethylamine | 16.7 | 17.5 | 24.6 | 26.6 |
| **4-chlorophenylisobutylamine** | Phenethylamine | 16.7 | 17.5 | 21.8 | 24.5 |
| **phenylisobutylamine** | Phenethylamine | 16.7 | 15.5 | 23.1 | 23.9 |
| **lysergic_acid_2-butyl_amide** | Tryptamine | 16.7 | 12.2 | 14.8 | 22.3 |
| **5-fluoro-n,n-dimethyltryptamine** | Tryptamine | 16.7 | 10.0 | 8.9 | 14.8 |
| **levomethamphetamine** | Amphetamine | 16.1 | 30.6 | 26.8 | 23.1 |
| **xylopropamine** | Amphetamine | 16.0 | 25.3 | 22.9 | 25.8 |
| **dexfenfluramine** | Amphetamine | 15.8 | 21.1 | 20.9 | 24.7 |
| **tiflorex** | Amphetamine | 15.8 | 14.9 | 20.3 | 23.1 |
| **(8beta)-n,n-diethyl-6-(prop-2-en-1-yl)-9,10-didehydroergoline-8-carboxamide** | Tryptamine | 15.4 | 23.2 | 21.9 | 20.2 |
| **(8beta)-n,n,6-triethyl-9,10-didehydroergoline-8-carboxamide** | Tryptamine | 15.2 | 14.3 | 14.1 | 12.9 |
| **benzphetamine** | Amphetamine | 14.9 | 13.0 | 16.7 | 12.8 |
| **dextrorphan** | Phenethylamine | 14.3 | 21.5 | 16.9 | 16.6 |
| **n,n-dibutyltryptamine** | Tryptamine | 13.3 | 9.7 | 7.9 | 7.4 |
| **2,5-dimethoxy-beta-hydroxy-4-methylphenethylamine** | Phenethylamine | 13.3 | 9.1 | 5.8 | 4.3 |
| **cryogenine** | Other | 12.7 | 12.7 | 13.0 | 11.1 |
| **methysergide** | Phenethylamine | 12.5 | 12.5 | 10.0 | 19.5 |
| **5-methoxydimethyltryptamine** | Tryptamine | 12.5 | 7.8 | 8.4 | 10.0 |
| **5-methoxy-1h-indole-3-propane-2-amine** | Tryptamine | 12.5 | 5.6 | 10.4 | 16.8 |
| **elemicin** | Other | 12.5 | 4.5 | 4.0 | 5.1 |
| **5-methoxy-n,n-methylisopropyltryptamine** | Tryptamine | 12.1 | 11.4 | 11.1 | 8.8 |
| **dibutyltryptamine** | Tryptamine | 11.7 | 9.8 | 10.2 | 7.4 |
| **diethyltryptamine** | Tryptamine | 11.6 | 8.0 | 11.9 | 17.2 |
| **2-(2,5-dimethoxy-4-butylphenyl)ethan-1-amine** | Phenethylamine | 11.5 | 25.6 | 27.4 | 25.9 |
| **levoamphetamine** | Amphetamine | 11.5 | 22.6 | 25.7 | 25.9 |
| **oxilofrine** | Phenethylamine | 11.1 | 10.0 | 9.5 | 14.8 |
| **n,n-dimethyl-2-(2-methyl-1h-indol-3-yl)ethanamine** | Tryptamine | 11.1 | 9.3 | 9.1 | 14.5 |
| **1-(5-methoxy-1h-indol-3-yl)-n-methyl-2-propanamine** | Tryptamine | 11.0 | 9.3 | 8.4 | 7.7 |
| **5-methoxy-n,n-diethyltryptamine** | Tryptamine | 10.7 | 10.4 | 10.6 | 7.5 |
| **para-iodoamphetamine** | Amphetamine | 10.0 | 24.2 | 29.3 | 26.3 |
| **ergoline-8beta-carboxamide,_9,10-didehydro-n,n-diethyl-1,6-dimethyl-** | Phenethylamine | 10.0 | 20.0 | 6.3 | 9.6 |
| **ergometrine** | Tryptamine | 10.0 | 6.5 | 4.9 | 7.4 |
| **1-(5-methoxy-1h-indol-3-yl)-2-butanamine** | Tryptamine | 9.8 | 9.2 | 9.1 | 14.9 |
| **melatonin** | Tryptamine | 9.8 | 8.5 | 8.8 | 16.0 |
| **5,n-dimethyl-n-isopropyltryptamine** | Tryptamine | 9.7 | 10.6 | 9.9 | 13.5 |
| **methylisopropyltryptamine** | Tryptamine | 9.7 | 6.3 | 11.9 | 14.1 |
| **amphetaminil** | Amphetamine | 9.7 | 12.3 | 14.0 | 15.3 |
| **2-(5-methoxy-2-methyl-1h-indol-3-yl)-n,n-dimethylethanamine** | Tryptamine | 9.6 | 8.3 | 10.4 | 12.8 |
| **5-methoxy-diisopropyltryptamine** | Tryptamine | 9.5 | 11.9 | 11.1 | 9.9 |
| **cabergoline** | Tryptamine | 8.7 | 8.0 | 10.8 | 2.7 |
| **norpholedrine** | Amphetamine | 8.3 | 24.3 | 15.8 | 11.9 |
| **methylenedioxypyrovalerone** | Cathinone | 8.3 | 2.8 | 9.3 | 10.4 |
| **methylergometrine** | Tryptamine | 8.0 | 4.8 | 2.6 | 4.4 |
| **harmaline** | Tryptamine | 7.4 | 10.6 | 15.9 | 13.4 |
| **n,n-diethyltryptamine** | Tryptamine | 7.4 | 8.4 | 17.9 | 17.9 |
| **harmine** | Other | 7.4 | 11.3 | 16.4 | 13.4 |
| **6,7-dihydro-5h-indeno(5,6-d)-1,3-dioxol-6-amine** | Phenethylamine | 7.1 | 8.3 | 7.7 | 6.8 |
| **1-naphthyl(1-pentyl-1h-indol-3-yl)methanone** | Other | 7.1 | 12.6 | 14.3 | 15.0 |
| **tetrahydroharmine** | Tryptamine | 6.8 | 13.8 | 15.9 | 16.9 |
| **alpha-methylserotonin** | Tryptamine | 6.3 | 5.0 | 9.1 | 9.3 |
| **mescaline** | Phenethylamine | 5.4 | 4.2 | 3.6 | 4.9 |
| **n-(2-fluorobenzyl)-2-(4-iodo-2,5-dimethoxyphenyl)ethanamine** | Phenethylamine | 4.0 | 10.1 | 10.1 | 12.0 |
| **bromocriptine** | Tryptamine | 3.2 | 3.2 | 2.7 | 4.7 |
| **epicriptine** | Tryptamine | 3.1 | 2.0 | 2.0 | 5.0 |
| **levonordefrin** | Phenethylamine | 3.1 | 4.9 | 5.8 | 4.8 |
| **isomescaline** | Phenethylamine | 3.0 | 5.5 | 5.5 | 5.4 |
| **(1-butyl-1h-indol-3-yl)(1-naphthyl)methanon** | Other | 2.6 | 9.8 | 17.5 | 13.9 |
| **ergotamine** | Tryptamine | 2.0 | 1.7 | 2.1 | 2.0 |
| **dihydroergocryptine** | Tryptamine | 2.0 | 2.0 | 4.2 | 9.6 |
| **2-(5-methoxy-2-methyl-1h-indol-3-yl)-n,n-dimethylethanamine** | Tryptamine | 1.5 | 9.3 | 12.3 | 13.3 |
| **dihydroergotamine** | Tryptamine | 1.3 | 1.5 | 1.3 | 5.5 |
| **1-[(7r)-3-bromo-2,5-dimethoxybicyclo[4.2.0]octa-1,3,5-trien-7-yl]methanamine** | Phenethylamine | 1.1 | 1.1 | 2.0 | 6.3 |
| **3,4-methylenedioxy-n-cyclopropylmethylamphetamine** | Amphetamine | 0.0 | 100.0 | 100.0 | 16.7 |
| **3,4-methylenedioxy-n-butylamphetamine** | Amphetamine | 0.0 | 100.0 | 16.7 | 9.1 |
| **2-ethylamino-1-(3,4-methylenedioxyphenyl)butane** | Phenethylamine | 0.0 | 100.0 | 11.0 | 9.5 |
| **methylenedioxyethylamphetamine** | Amphetamine | 0.0 | 100.0 | 7.7 | 17.5 |
| **2-methylamino-1-(3,4-methylenedioxyphenyl)pentane** | Phenethylamine | 0.0 | 100.0 | 7.1 | 12.9 |
| **para-methoxyethylamphetamine** | Amphetamine | 0.0 | 87.5 | 50.0 | 24.1 |
| **4-methoxy-n-methylamphetamine** | Amphetamine | 0.0 | 50.0 | 41.9 | 26.2 |
| **homopiperonylamine** | Phenethylamine | 0.0 | 50.0 | 2.2 | 7.2 |
| **alpha-ethyltryptamine** | Tryptamine | 0.0 | 47.4 | 41.5 | 25.7 |
| **2,3-methylenedioxyamphetamine** | Amphetamine | 0.0 | 43.1 | 44.2 | 16.1 |
| **jimscaline** | Phenethylamine | 0.0 | 42.9 | 35.7 | 3.9 |
| **3,4-ethylenedioxy-n-methylamphetamine** | Amphetamine | 0.0 | 42.9 | 23.1 | 18.2 |
| **tetralinylaminopropane** | Amphetamine | 0.0 | 41.9 | 46.5 | 27.7 |
| **6-(2-aminopropyl)-5-methoxy-1,3-benzoxathiol** | Amphetamine | 0.0 | 38.1 | 27.3 | 32.1 |
| **1-(3,5-dimethoxy-4-propoxyphenyl)-2-propanamine** | Amphetamine | 0.0 | 37.5 | 9.3 | 6.2 |
| **4-methyl-alpha-ethyltryptamine** | Tryptamine | 0.0 | 36.4 | 43.6 | 24.9 |
| **6-benzofuranethanamine,_2,3-dihydro-5-methoxy-alpha,2-dimethyl-** | Amphetamine | 0.0 | 35.7 | 30.2 | 28.5 |
| **1-(1-benzofuran-6-yl)-2-propanamine** | Amphetamine | 0.0 | 35.0 | 11.8 | 34.7 |
| **2,4-dimethoxy-5-methylthioamphetamine** | Amphetamine | 0.0 | 33.3 | 33.3 | 17.8 |
| **2-bromo-4,5-methylenedioxyamphetamine** | Amphetamine | 0.0 | 33.3 | 13.1 | 12.1 |
| **ethylisopropyltryptamine** | Tryptamine | 0.0 | 33.3 | 12.8 | 13.6 |
| **n-ethyl-n-[2-(1h-indol-3-yl)ethyl]-2-propanamine** | Tryptamine | 0.0 | 33.3 | 10.5 | 14.0 |
| **5-(2-aminopropyl)benzofuran** | Amphetamine | 0.0 | 30.8 | 31.2 | 29.7 |
| **trifluoromethylphenylpiperazine** | Other | 0.0 | 30.0 | 35.0 | 29.6 |
| **cathine** | Phenethylamine | 0.0 | 29.8 | 29.1 | 30.7 |
| **methedrone** | Cathinone | 0.0 | 29.5 | 22.4 | 18.8 |
| **3,6-dimethoxy-4-(2-aminoethyl)benzonorbornane** | Phenethylamine | 0.0 | 29.0 | 24.6 | 18.4 |
| **2,3-dimethoxy-4,5-methylenedioxyamphetamine** | Amphetamine | 0.0 | 28.6 | 28.6 | 28.6 |
| **1,4-dimethoxynaphthyl-2-isopropylamine** | Amphetamine | 0.0 | 28.6 | 22.2 | 14.9 |
| **2,5-dimethoxy-3,4-(trimethylene)phenethylamine;_5-(2-aminoethyl)-4,7-dimethoxyindane)** | Phenethylamine | 0.0 | 28.6 | 22.2 | 6.8 |
| **2,5-dimethoxy-3,4-(trimethylene)amphetamine** | Amphetamine | 0.0 | 28.6 | 22.2 | 5.6 |
| **3-[2-(diisopropylamino)ethyl]-1h-indol-4-ol** | Tryptamine | 0.0 | 26.3 | 10.7 | 7.4 |
| **propylisopropyltryptamine** | Tryptamine | 0.0 | 26.3 | 10.3 | 13.3 |
| **pholedrine** | Amphetamine | 0.0 | 25.6 | 19.6 | 15.2 |
| **ecstasy** | Amphetamine | 0.0 | 25.0 | 16.0 | 14.8 |
| **n-[2-(5,6-dimethoxy-1h-indol-3-yl)ethyl]-n-methyl-2-propanamine** | Tryptamine | 0.0 | 25.0 | 15.0 | 9.4 |
| **ethacetin** | Tryptamine | 0.0 | 25.0 | 10.0 | 18.3 |
| **3-methoxymethamphetamine** | Amphetamine | 0.0 | 25.0 | 4.7 | 25.2 |
| **eden** | Phenethylamine | 0.0 | 25.0 | 3.3 | 12.5 |
| **2,5-dimethoxy-3,4-(tetramethylene)phenethylamine;_6-(2-aminoethyl)-5,8-dimethoxy-tetralin** | Phenethylamine | 0.0 | 22.2 | 5.9 | 11.3 |
| **prenylamine** | Amphetamine | 0.0 | 20.0 | 18.1 | 14.1 |
| **(2s)-1-(3,4-dimethoxyphenyl)-2-propanamine** | Amphetamine | 0.0 | 20.0 | 7.1 | 16.2 |
| **o-acetylpsilocin** | Tryptamine | 0.0 | 18.2 | 22.3 | 21.4 |
| **morphinan** | Phenethylamine | 0.0 | 16.7 | 27.0 | 24.2 |
| **3-{2-[isopropyl(methyl)amino]ethyl}-1h-indol-4-yl_acetate** | Tryptamine | 0.0 | 16.7 | 16.2 | 18.0 |
| **n-ethyltryptamine** | Tryptamine | 0.0 | 16.7 | 14.3 | 22.3 |
| **(8beta)-6-cyclopropyl-n,n-diethyl-9,10-didehydroergoline-8-carboxamide** | Tryptamine | 0.0 | 15.4 | 15.4 | 14.1 |
| **3,4-methylenedioxyphentermine** | Amphetamine | 0.0 | 14.3 | 17.8 | 20.1 |
| **4-thiometaescaline** | Phenethylamine | 0.0 | 14.3 | 14.3 | 16.7 |
| **3-[2-(dibutylamino)ethyl]-1h-indol-4-ol** | Tryptamine | 0.0 | 14.3 | 10.7 | 8.3 |
| **2-(4-iodo-2,5-dimethoxyphenyl)ethanamine** | Phenethylamine | 0.0 | 13.3 | 11.1 | 2.2 |
| **nexus** | Phenethylamine | 0.0 | 13.3 | 7.4 | 6.1 |
| **n,n-dimethyl-2-[5-(methylsulfanyl)-1h-indol-3-yl]ethanamine** | Tryptamine | 0.0 | 12.8 | 11.6 | 15.9 |
| **etafedrine** | Phenethylamine | 0.0 | 12.5 | 16.7 | 22.9 |
| **methylone** | Cathinone | 0.0 | 11.1 | 22.2 | 12.5 |
| **methylenedioxyhydroxyamphetamine** | Amphetamine | 0.0 | 10.7 | 4.3 | 7.4 |
| **atropine** | Other | 0.0 | 10.7 | 10.2 | 12.6 |
| **[(8beta)-6-methyl-9,10-didehydroergolin-8-yl](4-morpholinyl)methanone** | Tryptamine | 0.0 | 10.5 | 10.0 | 7.8 |
| **(2s)-1-(1,3-benzodioxol-5-yl)-2-butanamine** | Phenethylamine | 0.0 | 10.1 | 11.9 | 9.8 |
| **indole,_3-[2-(dimethylamino)ethyl]-5-methyl-** | Tryptamine | 0.0 | 10.0 | 8.9 | 15.1 |
| **3-[2-(1-pyrrolidinyl)ethyl]-1h-indole** | Tryptamine | 0.0 | 9.8 | 8.8 | 20.1 |
| **aldosterone-stimulating_hormone** | Tryptamine | 0.0 | 9.7 | 11.4 | 12.4 |
| **lysergic_acid_hydroxyethylamide** | Tryptamine | 0.0 | 9.4 | 6.1 | 7.6 |
| **cafedrine** | Phenethylamine | 0.0 | 9.3 | 8.8 | 7.7 |
| **5h-1,3-dioxolo[4,5-f]indole-7-ethanamine,_n,n-dimethyl-** | Tryptamine | 0.0 | 9.3 | 8.0 | 12.3 |
| **dibenzylpiperazine** | Other | 0.0 | 9.1 | 6.8 | 4.3 |
| **3,4-methylenedioxy-n-hydroxyamphetamine** | Amphetamine | 0.0 | 8.9 | 4.3 | 7.4 |
| **propylamphetamine** | Amphetamine | 0.0 | 8.5 | 16.4 | 25.9 |
| **1-(5-methoxy-1h-indol-3-yl)-n-methyl-2-propanamine** | Tryptamine | 0.0 | 8.4 | 7.9 | 14.7 |
| **[3-[2-(diisopropylamino)ethyl]-1h-indol-4-yl]_acetate_hydrochloride** | Tryptamine | 0.0 | 8.3 | 9.6 | 11.6 |
| **n,n-diethyl-2-(2-methyl-1h-indol-3-yl)ethanamine** | Tryptamine | 0.0 | 8.3 | 20.0 | 22.6 |
| **diisopropyltryptamine** | Tryptamine | 0.0 | 7.9 | 11.6 | 14.2 |
| **naphyrone** | Cathinone | 0.0 | 7.6 | 10.4 | 7.5 |
| **metaproscaline** | Phenethylamine | 0.0 | 7.4 | 5.8 | 4.7 |
| **5-methoxy-3-[2-(pyrrolidin-1-yl)ethyl]-1h-indole** | Tryptamine | 0.0 | 7.4 | 6.3 | 9.4 |
| **phenescaline** | Phenethylamine | 0.0 | 7.3 | 7.5 | 10.1 |
| **4-methoxy-n-methyl-n-isopropyltryptamine** | Tryptamine | 0.0 | 7.1 | 10.3 | 13.8 |
| **allylescaline** | Phenethylamine | 0.0 | 7.1 | 7.1 | 7.1 |
| **1,4-dimethoxynaphthyl-2-ethylamine** | Phenethylamine | 0.0 | 6.9 | 12.3 | 10.0 |
| **methoxamine** | Phenethylamine | 0.0 | 6.9 | 2.5 | 3.3 |
| **phencyclidine** | Other | 0.0 | 6.3 | 33.7 | 34.9 |
| **2-bromo-lsd** | Tryptamine | 0.0 | 6.3 | 10.4 | 5.0 |
| **n-[[(7r)-3-bromo-2,5-dimethoxy-7-bicyclo[4.2.0]octa-1,3,5-trienyl]methyl]-1-(2-methoxyphenyl)methanamine** | Phenethylamine | 0.0 | 6.0 | 4.0 | 3.0 |
| **para-methoxymethamphetamine** | Amphetamine | 0.0 | 5.3 | 5.5 | 26.2 |
| **2-(4-iodo-2,5-dimethoxyphenyl)-n-(2-methoxybenzyl)ethanamine** | Phenethylamine | 0.0 | 5.3 | 1.5 | 5.0 |
| **[(8beta)-6-methyl-9,10-didehydroergolin-8-yl](pyrrolidin-1-yl)methanone** | Tryptamine | 0.0 | 5.1 | 19.8 | 9.5 |
| **norbaeocystin** | Other | 0.0 | 5.0 | 5.6 | 3.9 |
| **3,4-methylenedioxy-n-(2-hydroxyethyl)amphetamine** | Amphetamine | 0.0 | 5.0 | 2.6 | 3.0 |
| **alpha-methyldopamine** | Amphetamine | 0.0 | 4.9 | 5.1 | 4.9 |
| **para-methoxyphenylpiperazine** | Other | 0.0 | 4.8 | 21.1 | 22.0 |
| **escaline** | Phenethylamine | 0.0 | 4.6 | 4.6 | 3.2 |
| **pentorex** | Phenethylamine | 0.0 | 4.3 | 25.0 | 31.4 |
| **psilocin** | Tryptamine | 0.0 | 4.3 | 3.9 | 4.4 |
| **isoproscaline** | Phenethylamine | 0.0 | 4.2 | 4.5 | 4.0 |
| **phenescaline** | Phenethylamine | 0.0 | 4.2 | 7.3 | 2.8 |
| **asymbescaline** | Phenethylamine | 0.0 | 4.2 | 4.5 | 5.4 |
| **methylbenzodioxolylbutanamine** | Phenethylamine | 0.0 | 4.0 | 3.3 | 14.7 |
| **lysergic_acid_2,4-dimethylazetidide** | Tryptamine | 0.0 | 3.9 | 9.1 | 7.7 |
| **4-ethylamphetamine** | Amphetamine | 0.0 | 3.6 | 25.0 | 30.1 |
| **myristicin** | Other | 0.0 | 3.3 | 4.4 | 3.9 |
| **3,4,5-trimethoxyamphetamine** | Amphetamine | 0.0 | 3.1 | 5.5 | 4.5 |
| **proscaline** | Phenethylamine | 0.0 | 3.1 | 4.6 | 5.6 |
| **3,5-dimethoxy-4-(2-propynyloxy)phenethylamine** | Phenethylamine | 0.0 | 3.1 | 2.0 | 5.6 |
| **5h-1,3-dioxolo[4,5-f]indole-7-ethanamine,_n,n-bis(1-methylethyl)-** | Tryptamine | 0.0 | 3.1 | 5.5 | 9.4 |
| **4-bromo-2,5-dimethoxy-1-benzylpiperazine** | Other | 0.0 | 3.0 | 7.5 | 10.6 |
| **2,3,4-trimethoxyamphetamine** | Amphetamine | 0.0 | 3.0 | 4.3 | 5.5 |
| **symbescaline** | Phenethylamine | 0.0 | 3.0 | 4.1 | 3.9 |
| **fencamine** | Amphetamine | 0.0 | 2.7 | 3.0 | 8.5 |
| **4-methoxyamphetamine** | Amphetamine | 0.0 | 2.7 | 3.7 | 16.9 |
| **ergoloid** | Tryptamine | 0.0 | 2.7 | 2.1 | 3.9 |
| **4-hydroxy-5-methoxydimethyltryptamine** | Tryptamine | 0.0 | 2.1 | 2.3 | 5.1 |
| **4-allyloxy-3,5-dimethoxyphenethylamine** | Phenethylamine | 0.0 | 2.0 | 2.0 | 6.7 |
| **formoterol** | Amphetamine | 0.0 | 1.9 | 1.9 | 1.2 |
| **2-(4-chloro-2,5-dimethoxyphenyl)-n-(2-methoxybenzyl)ethanamine** | Phenethylamine | 0.0 | 1.6 | 3.5 | 4.8 |
| **2-(4-bromo-2,5-dimethoxyphenyl)-n-(2-methoxybenzyl)ethanamine** | Phenethylamine | 0.0 | 1.6 | 3.5 | 4.4 |
| **amfepentorex** | Amphetamine | 0.0 | 1.5 | 13.8 | 23.6 |
| **fenethylline** | Amphetamine | 0.0 | 1.3 | 3.7 | 4.2 |
| **2,5-dimethoxy-4-nitrophenethylamine** | Phenethylamine | 0.0 | 1.1 | 1.1 | 2.3 |
| **2,5-dimethoxy-4-nitrophenethylamine** | Phenethylamine | 0.0 | 1.1 | 1.0 | 2.3 |
| **3,4-methylenedioxy-2-methylthioamphetamine** | Amphetamine | 0.0 | 0.0 | 100.0 | 9.5 |
| **3,4-methylenedioxy-n,n-dimethylamphetamine** | Amphetamine | 0.0 | 0.0 | 50.0 | 7.9 |
| **2-methoxy-4-methyl-5-methylthioamphetamine** | Amphetamine | 0.0 | 0.0 | 36.8 | 35.4 |
| **1-(4,7-dimethoxy-1,3-benzodioxol-5-yl)propan-2-amine** | Amphetamine | 0.0 | 0.0 | 28.6 | 4.3 |
| **2,5-dimethoxy-n,n-dimethyl-4-iodoamphetamine** | Amphetamine | 0.0 | 0.0 | 25.0 | 34.8 |
| **2,5-dimethoxy-4-ethylthio-n-hydroxyphenethylamine** | Phenethylamine | 0.0 | 0.0 | 25.0 | 7.7 |
| **1-(2,3-dihydro-1-benzofuran-5-yl)propan-2-amine** | Amphetamine | 0.0 | 0.0 | 24.6 | 25.2 |
| **2,5-dimethoxy-3,4-(tetramethylene)amphetamine** | Amphetamine | 0.0 | 0.0 | 22.2 | 32.9 |
| **4-ethyl-2-methoxy-5-methylthioamphetamine** | Amphetamine | 0.0 | 0.0 | 21.6 | 15.7 |
| **4,5-dimethoxy-2-methylthioamphetamine** | Amphetamine | 0.0 | 0.0 | 21.4 | 13.2 |
| **indanylaminopropane** | Amphetamine | 0.0 | 0.0 | 20.9 | 30.8 |
| **4-ethyl-5-methoxy-2-methylthioamphetamine** | Amphetamine | 0.0 | 0.0 | 17.6 | 23.6 |
| **5-thioasymbescaline** | Phenethylamine | 0.0 | 0.0 | 16.7 | 13.5 |
| **ethylidenedioxyamphetamine** | Amphetamine | 0.0 | 0.0 | 15.4 | 13.6 |
| **2,4-dimethoxyamphetamine** | Amphetamine | 0.0 | 0.0 | 14.3 | 25.9 |
| **2-methoxy-n-methyl-4,5-methylenedioxyamphetamine** | Amphetamine | 0.0 | 0.0 | 14.3 | 23.7 |
| **para-methoxyamphetamine** | Amphetamine | 0.0 | 0.0 | 14.3 | 19.9 |
| **2,4,5-trimethoxyamphetamine** | Amphetamine | 0.0 | 0.0 | 14.3 | 7.4 |
| **4,5-dimethoxy-2-ethoxyamphetamine** | Phenethylamine | 0.0 | 0.0 | 14.3 | 5.8 |
| **3,4-methylenedioxy-n-benzylamphetamine** | Amphetamine | 0.0 | 0.0 | 14.0 | 13.2 |
| **3,5-dimethoxy-4-methylphenethylamine** | Phenethylamine | 0.0 | 0.0 | 13.3 | 6.3 |
| **2,5-dimethoxy-4-ethylphenethylamine** | Phenethylamine | 0.0 | 0.0 | 13.3 | 4.2 |
| **5-methoxy-4-methyl-2-methylthioamphetamine** | Amphetamine | 0.0 | 0.0 | 12.5 | 25.9 |
| **4-bromo-3,5-dimethoxyamphetamine** | Amphetamine | 0.0 | 0.0 | 11.1 | 12.8 |
| **benzodioxolylbutanamine** | Phenethylamine | 0.0 | 0.0 | 10.0 | 16.0 |
| **3,5-dimethoxy-4-ethoxyamphetamine** | Amphetamine | 0.0 | 0.0 | 9.3 | 6.7 |
| **3-{2-[methyl(propyl)amino]ethyl}-1h-indol-4-ol** | Tryptamine | 0.0 | 0.0 | 7.7 | 3.5 |
| **2,5-dimethoxy-4-phenylthioamphetamine** | Amphetamine | 0.0 | 0.0 | 7.1 | 6.3 |
| **methylenedioxymethylphenethylamine** | Phenethylamine | 0.0 | 0.0 | 5.9 | 8.3 |
| **6h-1,3-dioxolo[4,5-e]indole-8-ethanamine,_n,n-dimethyl-** | Tryptamine | 0.0 | 0.0 | 5.9 | 5.2 |
| **(6-methyl-9,10-didehydroergolin-8-yl)(1-piperidinyl)methanone** | Tryptamine | 0.0 | 0.0 | 5.8 | 7.8 |
| **baeocystin** | Tryptamine | 0.0 | 0.0 | 5.7 | 3.2 |
| **metaescaline** | Phenethylamine | 0.0 | 0.0 | 5.5 | 4.1 |
| **2-methoxy-4-methyl-5-methylsulfinylamphetamine** | Amphetamine | 0.0 | 0.0 | 5.0 | 11.8 |
| **1h-indol-4-ol,_3-[2-(ethylmethylamino)ethyl]-** | Tryptamine | 0.0 | 0.0 | 3.8 | 6.2 |
| **bufotenin** | Tryptamine | 0.0 | 0.0 | 3.4 | 7.1 |
| **4-benzyloxy-3,5-dimethoxyamphetamine** | Amphetamine | 0.0 | 0.0 | 3.0 | 7.6 |
| **buscaline** | Phenethylamine | 0.0 | 0.0 | 2.9 | 6.6 |
| **beta-methoxy-2c-b;_4-bromo-2,5-beta-trimethoxyphenethylamine** | Phenethylamine | 0.0 | 0.0 | 2.6 | 5.8 |
| **cyclopropylmescaline** | Phenethylamine | 0.0 | 0.0 | 2.0 | 6.6 |
| **trisescaline** | Phenethylamine | 0.0 | 0.0 | 2.0 | 6.2 |
| **benfluorex** | Amphetamine | 0.0 | 0.0 | 1.4 | 11.1 |
| **thiobuscaline** | Phenethylamine | 0.0 | 0.0 | 0.0 | 100.0 |
| **4-methyl-2,5-methoxyphenylcyclopropylamine** | Phenethylamine | 0.0 | 0.0 | 0.0 | 48.6 |
| **3-thiomescaline** | Phenethylamine | 0.0 | 0.0 | 0.0 | 33.3 |
| **1-(2,6-dimethoxy-4-methylphenyl)propan-2-amine** | Amphetamine | 0.0 | 0.0 | 0.0 | 29.6 |
| **(8beta)-n,n-diethyl-6-(2-propyn-1-yl)-9,10-didehydroergoline-8-carboxamide** | Tryptamine | 0.0 | 0.0 | 0.0 | 27.8 |
| **para-ethoxyamphetamine** | Amphetamine | 0.0 | 0.0 | 0.0 | 27.7 |
| **(2s)-1-(2,5-dimethoxyphenyl)-2-propanamine** | Amphetamine | 0.0 | 0.0 | 0.0 | 26.8 |
| **2,5-dimethoxy-4-ethylamphetamine** | Amphetamine | 0.0 | 0.0 | 0.0 | 26.4 |
| **2,5-dimethoxyphenethylamine** | Phenethylamine | 0.0 | 0.0 | 0.0 | 26.3 |
| **beatrice_(psychedelic)** | Amphetamine | 0.0 | 0.0 | 0.0 | 24.2 |
| **2,5-dimethoxy-4-chloroamphetamine** | Amphetamine | 0.0 | 0.0 | 0.0 | 23.2 |
| **2,5-dimethoxy-4-(n)-amylamphetamine** | Amphetamine | 0.0 | 0.0 | 0.0 | 21.6 |
| **2,5-dimethoxy-4-fluoroamphetamine** | Amphetamine | 0.0 | 0.0 | 0.0 | 20.5 |
| **2,5-dimethoxy-n-methylamphetamine** | Amphetamine | 0.0 | 0.0 | 0.0 | 20.2 |
| **2,n-dimethyl-4,5-methylenedioxyamphetamine** | Amphetamine | 0.0 | 0.0 | 0.0 | 20.0 |
| **4-bromo-2,5-dimethoxy-n-methylamphetamine** | Amphetamine | 0.0 | 0.0 | 0.0 | 19.8 |
| **2,5-dimethoxy-4-bromoamphetamine** | Amphetamine | 0.0 | 0.0 | 0.0 | 18.5 |
| **2,5-dimethoxy-4-methylamphetamine** | Amphetamine | 0.0 | 0.0 | 0.0 | 17.4 |
| **ecstasy** | Amphetamine | 0.0 | 0.0 | 0.0 | 16.9 |
| **2,5-dimethoxy-4-iodoamphetamine** | Amphetamine | 0.0 | 0.0 | 0.0 | 16.7 |
| **2-[4-(isopropylsulfanyl)-2,6-dimethoxyphenyl]ethanamine** | Phenethylamine | 0.0 | 0.0 | 0.0 | 16.7 |
| **3,4-methylenedioxy-n-(2-methoxyethyl)amphetamine** | Amphetamine | 0.0 | 0.0 | 0.0 | 16.7 |
| **furfenorex** | Amphetamine | 0.0 | 0.0 | 0.0 | 16.0 |
| **4-thiosymbescaline** | Phenethylamine | 0.0 | 0.0 | 0.0 | 15.4 |
| **1-(6-methyl-1,3-benzodioxol-5-yl)-2-propanamine** | Amphetamine | 0.0 | 0.0 | 0.0 | 14.8 |
| **2-methoxy-4,5-methylenedioxyamphetamine** | Amphetamine | 0.0 | 0.0 | 0.0 | 13.3 |
| **5-thiometaescaline** | Phenethylamine | 0.0 | 0.0 | 0.0 | 12.7 |
| **4-thiotrescaline** | Phenethylamine | 0.0 | 0.0 | 0.0 | 12.5 |
| **3,4-methylenedioxy-n-methyoxyamphetamine** | Amphetamine | 0.0 | 0.0 | 0.0 | 12.0 |
| **3-thioasymbescaline** | Phenethylamine | 0.0 | 0.0 | 0.0 | 11.9 |
| **3-methoxy-4-ethoxyphenethylamine** | Phenethylamine | 0.0 | 0.0 | 0.0 | 11.7 |
| **4-thioasymbescaline** | Phenethylamine | 0.0 | 0.0 | 0.0 | 10.5 |
| **3,4-methylenedioxyamphetamine** | Amphetamine | 0.0 | 0.0 | 0.0 | 9.7 |
| **2-[2,5-dimethoxy-4-(trifluoromethyl)phenyl]-n-(2-methoxybenzyl)ethanamine** | Phenethylamine | 0.0 | 0.0 | 0.0 | 9.4 |
| **2,5-dimethoxy-4-methylthiophenethylamine** | Phenethylamine | 0.0 | 0.0 | 0.0 | 9.4 |
| **1-(4-methyl-1,3-benzodioxol-6-yl)-2-aminopropane** | Amphetamine | 0.0 | 0.0 | 0.0 | 9.1 |
| **2-methyl-3,4-methylenedioxyamphetamine** | Amphetamine | 0.0 | 0.0 | 0.0 | 9.0 |
| **3-thiotrescaline** | Phenethylamine | 0.0 | 0.0 | 0.0 | 8.7 |
| **3-[2-(diethylamino)ethyl]-1h-indol-4-ol** | Tryptamine | 0.0 | 0.0 | 0.0 | 8.7 |
| **5-bromo-2,4-dimethoxyamphetamine** | Amphetamine | 0.0 | 0.0 | 0.0 | 7.7 |
| **3-{[(2r)-1-methyl-2-pyrrolidinyl]methyl}-1h-indol-4-ol** | Tryptamine | 0.0 | 0.0 | 0.0 | 7.2 |
| **2,5-dimethoxy-n-hydroxy-4-(n)-propylthiophenethylamine** | Phenethylamine | 0.0 | 0.0 | 0.0 | 7.1 |
| **1-(4-ethynyl-2,5-dimethoxyphenyl)-2-aminoethane** | Phenethylamine | 0.0 | 0.0 | 0.0 | 6.7 |
| **2,5-dimethoxy-4-(i)-propylthioamphetamine** | Amphetamine | 0.0 | 0.0 | 0.0 | 6.7 |
| **3-[2-(pyrrolidin-1-yl)ethyl]-1h-indol-4-ol** | Tryptamine | 0.0 | 0.0 | 0.0 | 6.6 |
| **benzeneethanamine,_4-(ethylthio)-3,5-dimethoxy-** | Phenethylamine | 0.0 | 0.0 | 0.0 | 6.1 |
| **methylenedioxyphenethylamine** | Phenethylamine | 0.0 | 0.0 | 0.0 | 5.8 |
| **2,4,5-trimethoxyphenethylamine** | Phenethylamine | 0.0 | 0.0 | 0.0 | 5.8 |
| **methallylescaline** | Phenethylamine | 0.0 | 0.0 | 0.0 | 5.6 |
| **3-thioescaline** | Phenethylamine | 0.0 | 0.0 | 0.0 | 5.6 |
| **n-[2-(5h-[1,3]dioxolo[4,5-f]indol-7-yl)ethyl]-n-methyl-2-propanamine** | Tryptamine | 0.0 | 0.0 | 0.0 | 5.3 |
| **2,5-dimethoxy-4-nitroamphetamine** | Amphetamine | 0.0 | 0.0 | 0.0 | 5.1 |
| **2,5-dimethoxy-4-(t)-butylthiophenethylamine** | Phenethylamine | 0.0 | 0.0 | 0.0 | 5.0 |
| **benzeneethanamine,_3,5-dimethoxy-4-(methylthio)-** | Phenethylamine | 0.0 | 0.0 | 0.0 | 4.9 |
| **2,5-dimethoxy-4-(n)-propylthioamphetamine** | Amphetamine | 0.0 | 0.0 | 0.0 | 4.8 |
| **3-{2-[methyl(propan-2-yl)amino]ethyl}-1h-indol-4-ol** | Tryptamine | 0.0 | 0.0 | 0.0 | 4.5 |
| **3-thiomescaline** | Phenethylamine | 0.0 | 0.0 | 0.0 | 4.5 |
| **ethocybin** | Tryptamine | 0.0 | 0.0 | 0.0 | 4.3 |
| **4-thioisomescaline** | Phenethylamine | 0.0 | 0.0 | 0.0 | 4.1 |
| **2,5-dimethoxy-4-ethoxyamphetamine** | Amphetamine | 0.0 | 0.0 | 0.0 | 3.8 |
| **lophophine** | Phenethylamine | 0.0 | 0.0 | 0.0 | 3.8 |
| **2,5-dimethoxy-4-(2-methoxyethylthio)phenethylamine** | Phenethylamine | 0.0 | 0.0 | 0.0 | 3.7 |
| **3-thiometaescaline** | Phenethylamine | 0.0 | 0.0 | 0.0 | 3.6 |
| **3-{2-[methyl(propan-2-yl)amino]ethyl}-1h-indol-4-ol** | Tryptamine | 0.0 | 0.0 | 0.0 | 3.5 |
| **4-bromomethcathinone** | Cathinone | 0.0 | 0.0 | 0.0 | 3.5 |
| **3-thiosymbescaline** | Phenethylamine | 0.0 | 0.0 | 0.0 | 3.4 |
| **2-thioisomescaline** | Phenethylamine | 0.0 | 0.0 | 0.0 | 3.3 |
| **2,5-diethoxy-4-methoxyamphetamine** | Phenethylamine | 0.0 | 0.0 | 0.0 | 3.0 |
| **4-methylthio-2-5-dimethoxyamphetamine** | Amphetamine | 0.0 | 0.0 | 0.0 | 2.9 |
| **aeruginascin** | Tryptamine | 0.0 | 0.0 | 0.0 | 2.9 |
| **3,4-dimethoxyphenethylamine** | Phenethylamine | 0.0 | 0.0 | 0.0 | 2.8 |
| **thioproscaline** | Phenethylamine | 0.0 | 0.0 | 0.0 | 2.8 |
| **2,3,6-trimethoxyamphetamine** | Amphetamine | 0.0 | 0.0 | 0.0 | 2.3 |
| **2,5-dimethoxy-3,4-dimethylamphetamine** | Amphetamine | 0.0 | 0.0 | 0.0 | 2.1 |
| **4-methoxy-2,3-methylenedioxyamphetamine** | Amphetamine | 0.0 | 0.0 | 0.0 | 1.0 |

## **Table S9**: Relationships between psychoactive classes and specific indications as calculated by the percentage of predictions for a given indication which belong to the given class.

| **MESH** | **Indication** | **Drug**  **count** | **Top 10** | **Top 25** | **Top 40** | **Top 100** |
| --- | --- | --- | --- | --- | --- | --- |
| Jet Lag Syndrome | Tryptamine | 1 | 100.00 | 100.00 | 100.00 | 63.64 |
| Seasonal Affective Disorder | Tryptamine | 1 | 100.00 | 100.00 | 100.00 | 63.64 |
| Sleep Disorders, Circadian Rhythm | Tryptamine | 1 | 100.00 | 100.00 | 100.00 | 63.64 |
| Binge-Eating Disorder | Amphetamine | 2 | 93.75 | 100.00 | 100.00 | 76.98 |
| Aphasia, Broca | Tryptamine | 1 | 44.44 | 58.33 | 69.23 | 55.56 |
| Narcolepsy | Amphetamine | 7 | 35.56 | 39.81 | 45.64 | 58.33 |
| Anorexia Nervosa | Amphetamine | 3 | 29.63 | 22.22 | 19.66 | 18.52 |
| Auditory Perceptual Disorders | Cathinone | 1 | 22.22 | 16.67 | 17.95 | 9.09 |
| Sleep-Wake Transition Disorders | Other | 1 | 22.22 | 8.33 | 5.13 | 2.02 |
| Epilepsies, Myoclonic | Amphetamine | 12 | 20.00 | 24.43 | 21.00 | 19.25 |
| Pica | Cathinone | 4 | 19.44 | 10.42 | 6.41 | 3.08 |
| Sleep Bruxism | Amphetamine | 3 | 18.52 | 22.06 | 18.35 | 17.31 |
| Sleep Bruxism | Tryptamine | 3 | 18.52 | 8.82 | 5.50 | 3.46 |
| Cataplexy | Amphetamine | 12 | 17.02 | 17.27 | 19.02 | 23.66 |
| Restless Legs Syndrome | Tryptamine | 10 | 16.67 | 22.06 | 24.37 | 25.43 |
| Dysthymic Disorder | Cathinone | 6 | 14.81 | 8.46 | 5.50 | 4.13 |
| Tic Disorders | Tryptamine | 5 | 13.89 | 7.29 | 7.05 | 6.19 |
| Bulimia Nervosa | Cathinone | 4 | 12.12 | 6.98 | 6.67 | 4.97 |
| Attention Deficit and Disruptive Behavior Disorders | Tryptamine | 5 | 11.11 | 14.58 | 17.31 | 15.42 |
| Pick Disease of the Brain | Cathinone | 1 | 11.11 | 8.33 | 7.69 | 4.04 |
| Alcohol-Related Disorders | Cathinone | 3 | 11.11 | 8.33 | 6.58 | 5.78 |
| Auditory Perceptual Disorders | Other | 1 | 11.11 | 8.33 | 5.13 | 5.05 |
| Attention Deficit Disorder with Hyperactivity | Tryptamine | 22 | 10.77 | 11.83 | 14.91 | 20.51 |
| Conduct Disorder | Amphetamine | 15 | 9.89 | 10.73 | 12.13 | 13.21 |
| Mood Disorders | Cathinone | 14 | 9.88 | 6.12 | 5.26 | 3.59 |
| Obsessive-Compulsive Disorder | Amphetamine | 13 | 9.78 | 10.55 | 11.60 | 18.85 |
| Restless Legs Syndrome | Cathinone | 10 | 9.52 | 5.88 | 5.06 | 3.41 |
| Personality Disorders | Amphetamine | 30 | 8.84 | 9.87 | 10.41 | 15.44 |
| Panic Disorder | Amphetamine | 19 | 8.18 | 10.50 | 12.20 | 21.40 |
| Depressive Disorder, Major | Amphetamine | 43 | 7.42 | 11.75 | 15.12 | 23.88 |
| Alcohol Withdrawal Delirium | Tryptamine | 8 | 7.41 | 10.29 | 12.39 | 11.70 |
| Eating Disorders | Cathinone | 3 | 7.41 | 5.56 | 5.98 | 5.54 |
| Restless Legs Syndrome | Amphetamine | 10 | 7.14 | 13.24 | 14.56 | 18.29 |
| Sleep Initiation and Maintenance Disorders | Tryptamine | 27 | 6.80 | 10.03 | 13.03 | 13.75 |
| Narcolepsy | Cathinone | 7 | 6.67 | 4.85 | 6.71 | 14.24 |
| Agoraphobia | Cathinone | 5 | 6.67 | 3.57 | 3.45 | 2.69 |
| Cataplexy | Tryptamine | 12 | 6.38 | 5.00 | 4.91 | 7.68 |
| Child Development Disorders, Pervasive | Amphetamine | 5 | 6.25 | 5.88 | 9.29 | 9.91 |
| Epilepsy, Complex Partial | Amphetamine | 14 | 6.12 | 7.98 | 7.31 | 11.52 |
| Amnesia | Amphetamine | 44 | 5.95 | 7.87 | 9.27 | 17.29 |
| Autistic Disorder | Tryptamine | 32 | 5.91 | 8.42 | 11.31 | 15.81 |
| Depressive Disorder, Major | Cathinone | 43 | 5.86 | 4.29 | 4.22 | 5.45 |
| Erectile Dysfunction | Tryptamine | 26 | 5.80 | 4.28 | 3.89 | 5.83 |
| Delirium | Cathinone | 8 | 5.63 | 3.76 | 4.56 | 2.82 |
| Dementia, Vascular | Amphetamine | 2 | 5.56 | 6.25 | 5.13 | 12.12 |
| Dyspareunia | Cathinone | 2 | 5.56 | 6.25 | 3.85 | 4.04 |
| Attention Deficit and Disruptive Behavior Disorders | Amphetamine | 5 | 5.56 | 5.21 | 8.33 | 8.23 |
| Myoclonic Epilepsy, Juvenile | Cathinone | 3 | 5.56 | 4.17 | 6.76 | 4.44 |
| Speech Disorders | Phenethylamine | 2 | 5.56 | 4.17 | 2.56 | 2.02 |
| Developmental Disabilities | Other | 4 | 5.56 | 3.26 | 4.08 | 2.69 |
| Tic Disorders | Other | 5 | 5.56 | 3.13 | 5.13 | 3.35 |
| Epilepsy | Amphetamine | 38 | 5.42 | 6.57 | 7.44 | 11.41 |
| Cognition Disorders | Tryptamine | 26 | 5.41 | 6.03 | 6.41 | 10.09 |
| Affective Disorders, Psychotic | Amphetamine | 9 | 5.17 | 3.68 | 3.88 | 4.10 |
| Learning Disorders | Tryptamine | 26 | 5.08 | 7.92 | 10.54 | 14.78 |
| Bipolar Disorder | Amphetamine | 42 | 5.08 | 6.10 | 6.03 | 10.63 |
| Depressive Disorder | Tryptamine | 65 | 5.02 | 4.99 | 6.44 | 10.90 |
| Autistic Disorder | Amphetamine | 32 | 5.00 | 6.11 | 6.61 | 16.24 |
| Anxiety Disorders | Amphetamine | 46 | 4.94 | 7.06 | 9.55 | 20.29 |
| Stereotypic Movement Disorder | Tryptamine | 16 | 4.80 | 4.21 | 5.13 | 6.50 |
| Seizures | Amphetamine | 94 | 4.59 | 8.31 | 11.24 | 22.29 |
| Panic Disorder | Cathinone | 19 | 4.55 | 2.94 | 4.17 | 5.01 |
| Narcolepsy | Other | 7 | 4.44 | 2.91 | 4.03 | 5.21 |
| Epilepsy, Tonic-Clonic | Amphetamine | 20 | 4.42 | 6.87 | 6.65 | 7.74 |
| Cocaine-Related Disorders | Cathinone | 75 | 4.39 | 3.67 | 3.50 | 5.19 |
| Erectile Dysfunction | Phenethylamine | 26 | 4.35 | 4.28 | 3.69 | 5.10 |
| Obsessive-Compulsive Disorder | Cathinone | 13 | 4.35 | 2.75 | 3.45 | 4.83 |
| Amphetamine-Related Disorders | Tryptamine | 27 | 4.21 | 5.62 | 5.98 | 10.41 |
| Cocaine-Related Disorders | Amphetamine | 75 | 4.17 | 8.02 | 9.98 | 18.96 |
| Phobic Disorders | Cathinone | 7 | 4.00 | 2.61 | 2.33 | 3.07 |
| Heroin Dependence | Cathinone | 11 | 3.90 | 3.55 | 3.26 | 3.63 |
| Attention Deficit Disorder with Hyperactivity | Other | 22 | 3.85 | 4.30 | 5.38 | 5.59 |
| Anxiety Disorders | Tryptamine | 46 | 3.80 | 5.16 | 8.10 | 11.57 |
| Anxiety Disorders | Cathinone | 46 | 3.80 | 3.44 | 3.99 | 5.33 |
| Epilepsies, Myoclonic | Tryptamine | 12 | 3.75 | 3.98 | 2.85 | 2.99 |
| Amphetamine-Related Disorders | Cathinone | 27 | 3.74 | 2.92 | 2.91 | 3.00 |
| Epilepsy, Rolandic | Phenethylamine | 4 | 3.70 | 2.82 | 1.83 | 1.55 |
| Alcoholic Intoxication | Cathinone | 3 | 3.70 | 2.78 | 1.71 | 1.56 |
| Sleep Bruxism | Cathinone | 3 | 3.70 | 1.47 | 3.67 | 2.69 |
| Epilepsy, Rolandic | Amphetamine | 4 | 3.70 | 1.41 | 0.92 | 0.78 |
| Eating Disorders | Amphetamine | 3 | 3.70 | 1.39 | 1.71 | 7.01 |
| Depressive Disorder | Amphetamine | 65 | 3.68 | 4.99 | 7.13 | 14.33 |
| Erectile Dysfunction | Amphetamine | 26 | 3.62 | 4.89 | 8.20 | 12.70 |
| Tourette Syndrome | Cathinone | 15 | 3.54 | 3.17 | 3.60 | 2.64 |
| Tobacco Use Disorder | Cathinone | 10 | 3.45 | 2.58 | 2.58 | 3.26 |
| Tobacco Use Disorder | Other | 10 | 3.45 | 1.94 | 2.15 | 2.65 |
| Amnesia | Tryptamine | 44 | 3.35 | 5.03 | 6.29 | 11.05 |
| Personality Disorders | Cathinone | 30 | 3.31 | 2.78 | 3.24 | 3.91 |
| Sexual Dysfunctions, Psychological | Cannabinoid | 11 | 3.30 | 1.32 | 0.84 | 0.39 |
| Cognition Disorders | Cathinone | 26 | 3.24 | 2.68 | 2.50 | 2.21 |
| Cataplexy | Other | 12 | 3.19 | 3.18 | 3.68 | 3.84 |
| Child Development Disorders, Pervasive | Other | 5 | 3.13 | 1.18 | 2.14 | 2.10 |
| Schizophrenia | Amphetamine | 65 | 3.10 | 3.60 | 4.27 | 7.11 |
| Attention Deficit Disorder with Hyperactivity | Phenethylamine | 22 | 3.08 | 2.51 | 2.20 | 2.93 |
| Cocaine-Related Disorders | Tryptamine | 75 | 3.07 | 6.90 | 10.24 | 20.75 |
| Seizures | Tryptamine | 94 | 3.06 | 2.93 | 4.33 | 12.61 |
| Bulimia Nervosa | Amphetamine | 4 | 3.03 | 9.30 | 11.85 | 18.21 |
| Bulimia Nervosa | Phenethylamine | 4 | 3.03 | 3.49 | 2.22 | 3.31 |
| Amnesia | Phenethylamine | 44 | 2.97 | 4.86 | 6.86 | 9.56 |
| Amnesia | Cathinone | 44 | 2.97 | 2.68 | 2.86 | 4.20 |
| Opioid-Related Disorders | Cathinone | 10 | 2.94 | 3.28 | 2.72 | 3.17 |
| Opioid-Related Disorders | Other | 10 | 2.94 | 2.73 | 2.38 | 2.06 |
| Dementia | Amphetamine | 13 | 2.90 | 3.83 | 2.69 | 7.81 |
| Erectile Dysfunction | Cathinone | 26 | 2.90 | 3.67 | 3.69 | 3.23 |
| Dementia | Cathinone | 13 | 2.90 | 1.64 | 1.35 | 1.65 |
| Learning Disorders | Cathinone | 26 | 2.82 | 2.48 | 2.17 | 2.19 |
| Pica | Amphetamine | 4 | 2.78 | 10.42 | 12.82 | 11.28 |
| Developmental Disabilities | Cathinone | 4 | 2.78 | 6.52 | 6.12 | 5.69 |
| Tic Disorders | Cathinone | 5 | 2.78 | 4.17 | 3.85 | 3.09 |
| Developmental Disabilities | Amphetamine | 4 | 2.78 | 3.26 | 2.72 | 11.68 |
| Amnesia, Anterograde | Phenethylamine | 4 | 2.78 | 3.19 | 3.47 | 3.64 |
| Tic Disorders | Phenethylamine | 5 | 2.78 | 2.08 | 1.28 | 0.52 |
| Attention Deficit and Disruptive Behavior Disorders | Other | 5 | 2.78 | 1.04 | 1.92 | 2.06 |
| Bipolar Disorder | Tryptamine | 42 | 2.73 | 2.15 | 3.85 | 8.06 |
| Substance Withdrawal Syndrome | Amphetamine | 48 | 2.73 | 4.57 | 5.24 | 11.47 |
| Status Epilepticus | Amphetamine | 29 | 2.69 | 3.99 | 5.32 | 9.79 |
| Tourette Syndrome | Cannabinoid | 15 | 2.65 | 1.06 | 0.90 | 0.74 |
| Consciousness Disorders | Amphetamine | 9 | 2.60 | 3.28 | 6.01 | 7.17 |
| Heroin Dependence | Other | 11 | 2.60 | 2.54 | 2.28 | 1.74 |
| Substance-Related Disorders | Cathinone | 11 | 2.56 | 2.09 | 2.05 | 2.19 |
| Substance-Related Disorders | Other | 11 | 2.56 | 1.57 | 2.05 | 1.72 |
| Epilepsies, Myoclonic | Phenethylamine | 12 | 2.50 | 2.84 | 4.27 | 3.58 |
| Psychoses, Substance-Induced | Cathinone | 14 | 2.47 | 1.96 | 2.61 | 1.49 |
| Mood Disorders | Phenethylamine | 14 | 2.47 | 1.53 | 1.75 | 4.27 |
| Mood Disorders | Other | 14 | 2.47 | 1.53 | 1.05 | 1.71 |
| Restless Legs Syndrome | Phenethylamine | 10 | 2.38 | 3.43 | 3.80 | 6.51 |
| Psychotic Disorders | Amphetamine | 47 | 2.34 | 2.47 | 3.82 | 6.65 |
| Attention Deficit Disorder with Hyperactivity | Cathinone | 22 | 2.31 | 3.94 | 3.67 | 5.46 |
| Autistic Disorder | Other | 32 | 2.27 | 1.68 | 2.64 | 2.56 |
| Narcolepsy | Phenethylamine | 7 | 2.22 | 6.80 | 10.07 | 8.68 |
| Agoraphobia | Amphetamine | 5 | 2.22 | 5.36 | 6.32 | 11.02 |
| Disorders of Excessive Somnolence | Phenethylamine | 5 | 2.22 | 3.54 | 2.33 | 1.98 |
| Agoraphobia | Phenethylamine | 5 | 2.22 | 2.68 | 1.72 | 1.88 |
| Seizures, Febrile | Phenethylamine | 8 | 2.22 | 1.72 | 1.13 | 1.06 |
| Seizures, Febrile | Amphetamine | 8 | 2.22 | 0.86 | 0.56 | 0.80 |
| Obsessive-Compulsive Disorder | Other | 13 | 2.17 | 2.29 | 3.45 | 3.74 |
| Cognition Disorders | Amphetamine | 26 | 2.16 | 3.57 | 5.16 | 9.67 |
| Child Behavior Disorders | Amphetamine | 14 | 2.15 | 2.20 | 3.78 | 5.33 |
| Status Epilepticus | Phenethylamine | 29 | 2.15 | 2.11 | 1.99 | 2.95 |
| Child Behavior Disorders | Phenethylamine | 14 | 2.15 | 1.32 | 1.16 | 2.02 |
| Cataplexy | Phenethylamine | 12 | 2.13 | 3.18 | 4.60 | 4.15 |
| Substance Withdrawal Syndrome | Other | 48 | 2.12 | 2.29 | 2.30 | 2.56 |
| Epilepsy | Phenethylamine | 38 | 2.08 | 4.50 | 5.18 | 7.76 |
| Epilepsy, Absence | Tryptamine | 12 | 2.06 | 2.13 | 1.62 | 3.98 |
| Epilepsy, Complex Partial | Cathinone | 14 | 2.04 | 1.41 | 2.99 | 2.50 |
| Depressive Disorder | Cathinone | 65 | 2.01 | 1.77 | 2.30 | 3.07 |
| Alzheimer Disease | Other | 32 | 1.90 | 1.73 | 1.54 | 1.56 |
| Alzheimer Disease | Cathinone | 32 | 1.90 | 1.35 | 1.41 | 1.34 |
| Dysthymic Disorder | Amphetamine | 6 | 1.85 | 6.92 | 9.50 | 9.63 |
| Alcoholism | Cathinone | 17 | 1.77 | 1.06 | 0.71 | 1.01 |
| Stress Disorders, Post-Traumatic | Cathinone | 21 | 1.74 | 1.22 | 1.12 | 1.17 |
| Affective Disorders, Psychotic | Phenethylamine | 9 | 1.72 | 2.94 | 1.94 | 3.13 |
| Affective Disorders, Psychotic | Cathinone | 9 | 1.72 | 1.47 | 1.46 | 1.20 |
| Schizophrenia, Paranoid | Amphetamine | 22 | 1.71 | 1.19 | 1.68 | 2.23 |
| Learning Disorders | Amphetamine | 26 | 1.69 | 2.48 | 2.84 | 5.93 |
| Epilepsy, Generalized | Phenethylamine | 7 | 1.69 | 1.42 | 0.97 | 0.91 |
| Epilepsy, Generalized | Amphetamine | 7 | 1.69 | 0.71 | 0.49 | 0.45 |
| Personality Disorders | Phenethylamine | 30 | 1.66 | 5.06 | 4.95 | 6.09 |
| Status Epilepticus | Tryptamine | 29 | 1.61 | 1.64 | 1.50 | 2.85 |
| Stereotypic Movement Disorder | Phenethylamine | 16 | 1.60 | 1.62 | 1.71 | 1.92 |
| Stereotypic Movement Disorder | Cathinone | 16 | 1.60 | 1.29 | 1.07 | 1.60 |
| Attention Deficit Disorder with Hyperactivity | Amphetamine | 22 | 1.54 | 3.23 | 4.16 | 10.92 |
| Seizures | Other | 94 | 1.53 | 2.15 | 3.26 | 4.79 |
| Anxiety Disorders | Other | 46 | 1.52 | 2.07 | 2.66 | 4.21 |
| Erectile Dysfunction | Other | 26 | 1.45 | 0.92 | 1.84 | 2.08 |
| Dementia | Other | 13 | 1.45 | 0.55 | 0.34 | 0.75 |
| Delirium | Amphetamine | 8 | 1.41 | 4.30 | 4.21 | 6.80 |
| Delirium | Other | 8 | 1.41 | 2.15 | 2.46 | 1.82 |
| Delirium | Phenethylamine | 8 | 1.41 | 1.08 | 1.75 | 2.65 |
| Amphetamine-Related Disorders | Amphetamine | 27 | 1.40 | 3.60 | 5.82 | 8.12 |
| Psychotic Disorders | Tryptamine | 47 | 1.40 | 2.47 | 4.46 | 12.11 |
| Psychotic Disorders | Phenethylamine | 47 | 1.40 | 2.02 | 1.75 | 3.28 |
| Amphetamine-Related Disorders | Phenethylamine | 27 | 1.40 | 1.80 | 1.94 | 3.71 |
| Schizophrenia | Tryptamine | 65 | 1.38 | 2.70 | 4.00 | 9.50 |
| Autistic Disorder | Cathinone | 32 | 1.36 | 2.53 | 3.52 | 5.04 |
| Seizures | Cathinone | 94 | 1.34 | 2.54 | 3.19 | 5.59 |
| Epilepsy, Temporal Lobe | Phenethylamine | 11 | 1.33 | 3.49 | 2.53 | 3.11 |
| Epilepsy, Temporal Lobe | Cathinone | 11 | 1.33 | 1.16 | 2.11 | 1.86 |
| Epilepsy, Temporal Lobe | Amphetamine | 11 | 1.33 | 1.16 | 0.84 | 4.97 |
| Cocaine-Related Disorders | Other | 75 | 1.32 | 2.90 | 3.67 | 4.44 |
| Consciousness Disorders | Phenethylamine | 9 | 1.30 | 1.64 | 1.77 | 2.80 |
| Consciousness Disorders | Other | 9 | 1.30 | 1.09 | 2.12 | 2.02 |
| Heroin Dependence | Cannabinoid | 11 | 1.30 | 0.51 | 1.30 | 1.02 |
| Substance-Related Disorders | Amphetamine | 11 | 1.28 | 0.52 | 1.03 | 2.82 |
| Substance-Related Disorders | Cannabinoid | 11 | 1.28 | 0.52 | 1.03 | 0.47 |
| Epilepsies, Myoclonic | Other | 12 | 1.25 | 2.84 | 2.49 | 2.39 |
| Epilepsy | Cathinone | 38 | 1.25 | 1.31 | 1.86 | 2.48 |
| Epilepsies, Myoclonic | Cathinone | 12 | 1.25 | 1.14 | 2.49 | 3.88 |
| Epilepsy | Cannabinoid | 38 | 1.25 | 0.56 | 0.53 | 0.47 |
| Mood Disorders | Amphetamine | 14 | 1.23 | 5.61 | 7.72 | 12.82 |
| Psychoses, Substance-Induced | Other | 14 | 1.23 | 1.47 | 2.61 | 1.64 |
| Psychoses, Substance-Induced | Phenethylamine | 14 | 1.23 | 0.98 | 1.31 | 2.08 |
| Substance Withdrawal Syndrome | Cathinone | 48 | 1.21 | 1.43 | 2.30 | 3.47 |
| Seizures | Phenethylamine | 94 | 1.15 | 2.15 | 3.49 | 7.61 |
| Huntington Disease | Phenethylamine | 10 | 1.14 | 1.34 | 1.12 | 1.59 |
| Learning Disorders | Other | 26 | 1.13 | 2.48 | 2.51 | 2.37 |
| Amnesia | Other | 44 | 1.12 | 1.51 | 1.49 | 2.31 |
| Conduct Disorder | Cathinone | 15 | 1.10 | 1.46 | 2.30 | 3.59 |
| Conduct Disorder | Other | 15 | 1.10 | 0.98 | 1.64 | 2.77 |
| Obsessive-Compulsive Disorder | Phenethylamine | 13 | 1.09 | 2.75 | 2.19 | 3.27 |
| Sleep Disorders | Tryptamine | 14 | 1.09 | 1.49 | 3.62 | 7.42 |
| Sleep Disorders | Other | 14 | 1.09 | 1.49 | 3.29 | 3.87 |
| Sleep Disorders | Phenethylamine | 14 | 1.09 | 1.49 | 1.64 | 1.94 |
| Sleep Disorders | Amphetamine | 14 | 1.09 | 1.00 | 0.99 | 2.26 |
| Status Epilepticus | Other | 29 | 1.08 | 1.41 | 1.99 | 1.81 |
| Child Behavior Disorders | Other | 14 | 1.08 | 0.44 | 1.74 | 1.87 |
| Cataplexy | Cathinone | 12 | 1.06 | 2.27 | 3.07 | 6.45 |
| Epilepsy, Absence | Amphetamine | 12 | 1.03 | 2.98 | 4.05 | 6.80 |
| Epilepsy, Absence | Cathinone | 12 | 1.03 | 0.85 | 1.35 | 1.16 |
| Epilepsy, Absence | Other | 12 | 1.03 | 0.43 | 1.08 | 1.03 |
| Epilepsy, Complex Partial | Phenethylamine | 14 | 1.02 | 3.76 | 2.99 | 4.51 |
| Alzheimer Disease | Tryptamine | 32 | 0.95 | 3.85 | 6.94 | 13.14 |
| Alzheimer Disease | Amphetamine | 32 | 0.95 | 2.50 | 3.60 | 7.94 |
| Psychotic Disorders | Other | 47 | 0.93 | 1.35 | 3.66 | 4.37 |
| Psychotic Disorders | Cathinone | 47 | 0.93 | 0.90 | 1.43 | 2.09 |
| Panic Disorder | Phenethylamine | 19 | 0.91 | 2.52 | 2.08 | 4.86 |
| Substance Withdrawal Syndrome | Phenethylamine | 48 | 0.91 | 1.86 | 2.41 | 4.59 |
| Tourette Syndrome | Other | 15 | 0.88 | 1.41 | 1.57 | 1.48 |
| Epilepsy, Tonic-Clonic | Other | 20 | 0.88 | 1.29 | 1.21 | 2.17 |
| Epilepsy, Tonic-Clonic | Cathinone | 20 | 0.88 | 0.43 | 1.51 | 1.55 |
| Stress Disorders, Post-Traumatic | Phenethylamine | 21 | 0.87 | 2.44 | 1.68 | 3.22 |
| Stress Disorders, Post-Traumatic | Amphetamine | 21 | 0.87 | 1.22 | 1.12 | 2.34 |
| Schizophrenia, Paranoid | Phenethylamine | 22 | 0.85 | 1.58 | 1.12 | 1.93 |
| Stereotypic Movement Disorder | Amphetamine | 16 | 0.80 | 2.91 | 2.78 | 5.97 |
| Depressive Disorder, Major | Tryptamine | 43 | 0.78 | 3.17 | 6.40 | 12.58 |
| Epilepsies, Partial | Phenethylamine | 19 | 0.78 | 3.03 | 2.07 | 2.52 |
| Depressive Disorder, Major | Other | 43 | 0.78 | 2.80 | 3.27 | 4.01 |
| Epilepsies, Partial | Cathinone | 19 | 0.78 | 1.14 | 1.55 | 1.59 |
| Bipolar Disorder | Other | 42 | 0.78 | 1.08 | 1.15 | 1.96 |
| Epilepsies, Partial | Amphetamine | 19 | 0.78 | 0.76 | 0.52 | 3.45 |
| Bipolar Disorder | Cathinone | 42 | 0.78 | 0.54 | 1.54 | 2.64 |
| Schizophrenia | Other | 65 | 0.69 | 1.62 | 2.67 | 2.80 |
| Schizophrenia | Cathinone | 65 | 0.69 | 0.72 | 0.93 | 2.64 |
| Sleep Initiation and Maintenance Disorders | Other | 27 | 0.68 | 2.51 | 3.99 | 3.20 |
| Cocaine-Related Disorders | Phenethylamine | 75 | 0.66 | 2.67 | 3.75 | 6.28 |
| Learning Disorders | Phenethylamine | 26 | 0.56 | 0.99 | 1.67 | 2.28 |
| Personality Disorders | Other | 30 | 0.55 | 1.27 | 1.37 | 2.36 |
| Cognition Disorders | Other | 26 | 0.54 | 2.46 | 2.66 | 1.87 |
| Status Epilepticus | Cathinone | 29 | 0.54 | 1.41 | 1.83 | 2.57 |
| Alzheimer Disease | Phenethylamine | 32 | 0.47 | 1.92 | 2.70 | 5.05 |
| Amphetamine-Related Disorders | Other | 27 | 0.47 | 0.90 | 1.13 | 1.77 |
| Epilepsy | Other | 38 | 0.42 | 0.94 | 1.59 | 2.48 |
| Depressive Disorder, Major | Phenethylamine | 43 | 0.39 | 2.99 | 3.13 | 6.57 |
| Bipolar Disorder | Phenethylamine | 42 | 0.39 | 1.80 | 1.79 | 3.84 |
| Depressive Disorder, Major | Cannabinoid | 43 | 0.39 | 0.19 | 0.41 | 0.24 |
| Anxiety Disorders | Phenethylamine | 46 | 0.38 | 2.41 | 2.30 | 5.41 |
| Schizophrenia | Phenethylamine | 65 | 0.34 | 1.62 | 2.00 | 2.64 |
| Substance Withdrawal Syndrome | Tryptamine | 48 | 0.30 | 2.14 | 3.77 | 8.98 |
| Substance Withdrawal Syndrome | Cannabinoid | 48 | 0.30 | 0.14 | 0.31 | 0.33 |
| Cocaine-Related Disorders | Cannabinoid | 75 | 0.22 | 0.11 | 0.34 | 0.58 |
| Auditory Perceptual Disorders | Amphetamine | 1 | 0.00 | 29.17 | 28.21 | 37.37 |
| Binge-Eating Disorder | Phenethylamine | 2 | 0.00 | 12.50 | 22.73 | 13.49 |
| Gambling | Amphetamine | 1 | 0.00 | 12.50 | 7.69 | 26.26 |
| Disorders of Sex Development | Other | 1 | 0.00 | 8.33 | 5.13 | 2.02 |
| Speech Disorders | Tryptamine | 2 | 0.00 | 6.25 | 11.54 | 15.15 |
| Alcohol-Related Disorders | Other | 3 | 0.00 | 6.25 | 6.58 | 4.05 |
| Amnesia, Anterograde | Tryptamine | 4 | 0.00 | 5.32 | 9.03 | 15.15 |
| Heroin Dependence | Tryptamine | 11 | 0.00 | 4.57 | 5.86 | 6.68 |
| Alcohol-Related Disorders | Tryptamine | 3 | 0.00 | 4.17 | 9.21 | 8.09 |
| Depression, Postpartum | Other | 1 | 0.00 | 4.17 | 7.69 | 3.03 |
| Myoclonic Epilepsy, Juvenile | Amphetamine | 3 | 0.00 | 4.17 | 5.41 | 16.11 |
| Dementia, Vascular | Phenethylamine | 2 | 0.00 | 4.17 | 5.13 | 4.55 |
| Neurotic Disorders | Tryptamine | 3 | 0.00 | 4.17 | 5.13 | 2.87 |
| Myoclonic Epilepsy, Juvenile | Phenethylamine | 3 | 0.00 | 4.17 | 4.05 | 7.22 |
| Dyspareunia | Amphetamine | 2 | 0.00 | 4.17 | 2.56 | 5.05 |
| Dementia, Multi-Infarct | Tryptamine | 1 | 0.00 | 4.17 | 2.56 | 4.04 |
| Epilepsy, Reflex | Cathinone | 2 | 0.00 | 4.17 | 2.56 | 3.03 |
| Pick Disease of the Brain | Amphetamine | 1 | 0.00 | 4.17 | 2.56 | 3.03 |
| Psychoses, Substance-Induced | Amphetamine | 14 | 0.00 | 3.43 | 3.59 | 6.10 |
| Morphine Dependence | Phenethylamine | 5 | 0.00 | 3.37 | 2.48 | 1.65 |
| Tourette Syndrome | Amphetamine | 15 | 0.00 | 3.17 | 2.92 | 6.86 |
| Neurotic Disorders | Cathinone | 3 | 0.00 | 2.78 | 2.56 | 2.51 |
| Consciousness Disorders | Tryptamine | 9 | 0.00 | 2.73 | 6.36 | 8.72 |
| Opioid-Related Disorders | Tryptamine | 10 | 0.00 | 2.73 | 5.78 | 7.61 |
| Disorders of Excessive Somnolence | Tryptamine | 5 | 0.00 | 2.65 | 5.81 | 10.64 |
| Substance-Related Disorders | Tryptamine | 11 | 0.00 | 2.62 | 2.74 | 4.70 |
| Phobic Disorders | Other | 7 | 0.00 | 2.61 | 2.91 | 2.45 |
| Spasms, Infantile | Phenethylamine | 8 | 0.00 | 2.59 | 1.76 | 0.80 |
| Psychoses, Substance-Induced | Tryptamine | 14 | 0.00 | 2.45 | 4.25 | 9.08 |
| Delirium | Tryptamine | 8 | 0.00 | 2.15 | 4.91 | 9.12 |
| Amnesia, Anterograde | Other | 4 | 0.00 | 2.13 | 3.47 | 1.52 |
| Epilepsy, Absence | Phenethylamine | 12 | 0.00 | 2.13 | 1.62 | 3.47 |
| Alcoholism | Phenethylamine | 17 | 0.00 | 2.12 | 1.42 | 2.53 |
| Hypochondriasis | Tryptamine | 2 | 0.00 | 2.08 | 2.56 | 4.04 |
| Alcohol-Related Disorders | Amphetamine | 3 | 0.00 | 2.08 | 1.32 | 2.31 |
| Heroin Dependence | Phenethylamine | 11 | 0.00 | 2.03 | 1.30 | 1.31 |
| Restless Legs Syndrome | Other | 10 | 0.00 | 1.96 | 1.58 | 1.55 |
| Tobacco Use Disorder | Tryptamine | 10 | 0.00 | 1.94 | 2.58 | 8.35 |
| Agoraphobia | Other | 5 | 0.00 | 1.79 | 1.72 | 1.61 |
| Huntington Disease | Amphetamine | 10 | 0.00 | 1.79 | 1.40 | 5.16 |
| Depressive Disorder | Phenethylamine | 65 | 0.00 | 1.77 | 1.84 | 5.52 |
| Stress Disorders, Post-Traumatic | Other | 21 | 0.00 | 1.63 | 3.35 | 3.36 |
| Personality Disorders | Tryptamine | 30 | 0.00 | 1.52 | 2.73 | 5.36 |
| Affective Disorders, Psychotic | Other | 9 | 0.00 | 1.47 | 2.91 | 2.41 |
| Sleep Bruxism | Phenethylamine | 3 | 0.00 | 1.47 | 1.83 | 3.46 |
| Sleep Bruxism | Other | 3 | 0.00 | 1.47 | 0.92 | 0.77 |
| Conduct Disorder | Phenethylamine | 15 | 0.00 | 1.46 | 2.30 | 2.28 |
| Tourette Syndrome | Tryptamine | 15 | 0.00 | 1.41 | 2.02 | 6.12 |
| Tourette Syndrome | Phenethylamine | 15 | 0.00 | 1.41 | 1.57 | 2.75 |
| Neurotic Disorders | Amphetamine | 3 | 0.00 | 1.39 | 5.13 | 7.53 |
| Anorexia Nervosa | Phenethylamine | 3 | 0.00 | 1.39 | 1.71 | 3.03 |
| Anorexia Nervosa | Other | 3 | 0.00 | 1.39 | 0.85 | 1.01 |
| Alcoholic Intoxication | Phenethylamine | 3 | 0.00 | 1.39 | 0.85 | 0.78 |
| Huntington Disease | Tryptamine | 10 | 0.00 | 1.34 | 3.35 | 7.94 |
| Sexual Dysfunctions, Psychological | Phenethylamine | 11 | 0.00 | 1.32 | 1.12 | 1.44 |
| Sexual Dysfunctions, Psychological | Amphetamine | 11 | 0.00 | 1.32 | 0.84 | 3.66 |
| Stereotypic Movement Disorder | Other | 16 | 0.00 | 1.29 | 1.07 | 1.07 |
| Tobacco Use Disorder | Amphetamine | 10 | 0.00 | 1.29 | 1.72 | 6.11 |
| Tobacco Use Disorder | Phenethylamine | 10 | 0.00 | 1.29 | 0.86 | 1.02 |
| Autistic Disorder | Phenethylamine | 32 | 0.00 | 1.26 | 1.62 | 3.25 |
| Sleep Initiation and Maintenance Disorders | Cathinone | 27 | 0.00 | 1.25 | 1.26 | 1.17 |
| Nocturnal Enuresis | Amphetamine | 4 | 0.00 | 1.23 | 2.99 | 3.26 |
| Child Development Disorders, Pervasive | Cathinone | 5 | 0.00 | 1.18 | 2.86 | 3.00 |
| Dementia | Tryptamine | 13 | 0.00 | 1.09 | 3.03 | 6.31 |
| Opioid-Related Disorders | Amphetamine | 10 | 0.00 | 1.09 | 2.38 | 5.23 |
| Opioid-Related Disorders | Phenethylamine | 10 | 0.00 | 1.09 | 0.68 | 1.27 |
| Developmental Disabilities | Phenethylamine | 4 | 0.00 | 1.09 | 2.04 | 1.50 |
| Alcoholism | Tryptamine | 17 | 0.00 | 1.06 | 1.89 | 3.54 |
| Attention Deficit and Disruptive Behavior Disorders | Cathinone | 5 | 0.00 | 1.04 | 2.56 | 2.57 |
| Alcohol Withdrawal Seizures | Amphetamine | 4 | 0.00 | 1.04 | 1.32 | 1.65 |
| Pica | Tryptamine | 4 | 0.00 | 1.04 | 1.28 | 4.36 |
| Pica | Other | 4 | 0.00 | 1.04 | 0.64 | 0.77 |
| Mood Disorders | Tryptamine | 14 | 0.00 | 1.02 | 1.40 | 6.84 |
| Heroin Dependence | Amphetamine | 11 | 0.00 | 1.02 | 1.30 | 3.63 |
| Depressive Disorder | Other | 65 | 0.00 | 0.97 | 1.84 | 3.42 |
| Enuresis | Amphetamine | 7 | 0.00 | 0.93 | 1.16 | 2.63 |
| Enuresis | Phenethylamine | 7 | 0.00 | 0.93 | 0.58 | 0.48 |
| Disorders of Excessive Somnolence | Other | 5 | 0.00 | 0.88 | 1.74 | 0.99 |
| Sexual Dysfunctions, Psychological | Tryptamine | 11 | 0.00 | 0.88 | 1.68 | 4.06 |
| Child Behavior Disorders | Tryptamine | 14 | 0.00 | 0.88 | 1.16 | 3.75 |
| Phobic Disorders | Amphetamine | 7 | 0.00 | 0.87 | 0.58 | 0.92 |
| Phobic Disorders | Phenethylamine | 7 | 0.00 | 0.87 | 0.58 | 0.31 |
| Epilepsy, Tonic-Clonic | Phenethylamine | 20 | 0.00 | 0.86 | 0.91 | 2.48 |
| Dysthymic Disorder | Phenethylamine | 6 | 0.00 | 0.77 | 1.00 | 2.75 |
| Dysthymic Disorder | Other | 6 | 0.00 | 0.77 | 0.50 | 1.83 |
| Impulse Control Disorders | Tryptamine | 8 | 0.00 | 0.76 | 4.35 | 7.93 |
| Impulse Control Disorders | Other | 8 | 0.00 | 0.76 | 0.97 | 0.63 |
| Alcoholism | Cannabinoid | 17 | 0.00 | 0.71 | 0.71 | 0.76 |
| Sleep Initiation and Maintenance Disorders | Amphetamine | 27 | 0.00 | 0.63 | 1.68 | 2.99 |
| Dementia | Phenethylamine | 13 | 0.00 | 0.55 | 1.68 | 1.80 |
| Consciousness Disorders | Cathinone | 9 | 0.00 | 0.55 | 1.41 | 2.49 |
| Sleep Disorders | Cathinone | 14 | 0.00 | 0.50 | 0.66 | 1.13 |
| Epilepsy, Complex Partial | Other | 14 | 0.00 | 0.47 | 1.33 | 1.84 |
| Huntington Disease | Other | 10 | 0.00 | 0.45 | 1.40 | 1.19 |
| Child Behavior Disorders | Cathinone | 14 | 0.00 | 0.44 | 1.16 | 1.87 |
| Panic Disorder | Other | 19 | 0.00 | 0.42 | 2.08 | 3.95 |
| Schizophrenia, Paranoid | Other | 22 | 0.00 | 0.40 | 1.68 | 2.52 |
| Epilepsy | Tryptamine | 38 | 0.00 | 0.38 | 0.66 | 3.96 |
| Sleep Initiation and Maintenance Disorders | Phenethylamine | 27 | 0.00 | 0.31 | 0.63 | 1.81 |
| Cognition Disorders | Phenethylamine | 26 | 0.00 | 0.22 | 1.25 | 3.05 |
| Motor Skills Disorders | Phenethylamine | 1 | 0.00 | 0.00 | 5.13 | 13.13 |
| Dementia, Vascular | Tryptamine | 2 | 0.00 | 0.00 | 5.13 | 10.61 |
| Gambling | Tryptamine | 1 | 0.00 | 0.00 | 5.13 | 7.07 |
| Auditory Perceptual Disorders | Phenethylamine | 1 | 0.00 | 0.00 | 5.13 | 6.06 |
| Motor Skills Disorders | Amphetamine | 1 | 0.00 | 0.00 | 5.13 | 3.03 |
| Binge-Eating Disorder | Other | 2 | 0.00 | 0.00 | 4.55 | 2.38 |
| Borderline Personality Disorder | Other | 4 | 0.00 | 0.00 | 3.53 | 2.13 |
| Nocturnal Enuresis | Tryptamine | 4 | 0.00 | 0.00 | 2.99 | 3.56 |
| Bulimia Nervosa | Tryptamine | 4 | 0.00 | 0.00 | 2.96 | 4.64 |
| Stress Disorders, Post-Traumatic | Tryptamine | 21 | 0.00 | 0.00 | 2.79 | 8.19 |
| Gambling | Phenethylamine | 1 | 0.00 | 0.00 | 2.56 | 4.04 |
| Dementia, Multi-Infarct | Other | 1 | 0.00 | 0.00 | 2.56 | 3.03 |
| Dementia, Multi-Infarct | Amphetamine | 1 | 0.00 | 0.00 | 2.56 | 2.02 |
| Trichotillomania | Tryptamine | 1 | 0.00 | 0.00 | 2.56 | 2.02 |
| Psychoses, Alcoholic | Cathinone | 1 | 0.00 | 0.00 | 2.56 | 1.01 |
| Binge-Eating Disorder | Cathinone | 2 | 0.00 | 0.00 | 2.27 | 11.90 |
| Schizophrenia, Catatonic | Other | 5 | 0.00 | 0.00 | 2.13 | 1.38 |
| Developmental Disabilities | Tryptamine | 4 | 0.00 | 0.00 | 2.04 | 3.59 |
| Epilepsy, Rolandic | Other | 4 | 0.00 | 0.00 | 1.83 | 2.33 |
| Anorexia Nervosa | Cathinone | 3 | 0.00 | 0.00 | 1.71 | 3.03 |
| Eating Disorders | Phenethylamine | 3 | 0.00 | 0.00 | 1.71 | 1.48 |
| Panic Disorder | Tryptamine | 19 | 0.00 | 0.00 | 1.49 | 6.22 |
| Amnesia, Anterograde | Amphetamine | 4 | 0.00 | 0.00 | 1.39 | 2.73 |
| Alcohol Withdrawal Delirium | Other | 8 | 0.00 | 0.00 | 1.38 | 1.49 |
| REM Sleep Behavior Disorder | Other | 2 | 0.00 | 0.00 | 1.30 | 2.86 |
| Hypochondriasis | Cannabinoid | 2 | 0.00 | 0.00 | 1.28 | 1.01 |
| Speech Disorders | Other | 2 | 0.00 | 0.00 | 1.28 | 0.51 |
| Epilepsy, Temporal Lobe | Other | 11 | 0.00 | 0.00 | 1.27 | 2.07 |
| Alcoholism | Other | 17 | 0.00 | 0.00 | 1.18 | 1.39 |
| Seizures, Febrile | Other | 8 | 0.00 | 0.00 | 1.13 | 2.93 |
| Epilepsy, Generalized | Other | 7 | 0.00 | 0.00 | 0.97 | 1.59 |
| Epilepsy, Rolandic | Tryptamine | 4 | 0.00 | 0.00 | 0.92 | 2.71 |
| Neurotic Disorders | Phenethylamine | 3 | 0.00 | 0.00 | 0.85 | 2.51 |
| Eating Disorders | Other | 3 | 0.00 | 0.00 | 0.85 | 1.11 |
| Neurotic Disorders | Other | 3 | 0.00 | 0.00 | 0.85 | 1.08 |
| Epilepsies, Partial | Other | 19 | 0.00 | 0.00 | 0.78 | 1.46 |
| Bulimia Nervosa | Other | 4 | 0.00 | 0.00 | 0.74 | 1.66 |
| Substance-Related Disorders | Phenethylamine | 11 | 0.00 | 0.00 | 0.68 | 0.63 |
| Alcohol Withdrawal Seizures | Tryptamine | 4 | 0.00 | 0.00 | 0.66 | 2.75 |
| Alcohol Withdrawal Seizures | Cathinone | 4 | 0.00 | 0.00 | 0.66 | 0.83 |
| Pica | Phenethylamine | 4 | 0.00 | 0.00 | 0.64 | 3.08 |
| Pica | Cannabinoid | 4 | 0.00 | 0.00 | 0.64 | 0.51 |
| Spasms, Infantile | Other | 8 | 0.00 | 0.00 | 0.59 | 1.59 |
| Phobic Disorders | Tryptamine | 7 | 0.00 | 0.00 | 0.58 | 1.84 |
| Agoraphobia | Tryptamine | 5 | 0.00 | 0.00 | 0.57 | 1.61 |
| Seizures, Febrile | Tryptamine | 8 | 0.00 | 0.00 | 0.56 | 3.19 |
| Affective Disorders, Psychotic | Tryptamine | 9 | 0.00 | 0.00 | 0.49 | 2.65 |
| Epilepsy, Generalized | Tryptamine | 7 | 0.00 | 0.00 | 0.49 | 1.59 |
| Impulse Control Disorders | Cannabinoid | 8 | 0.00 | 0.00 | 0.48 | 0.63 |
| Impulse Control Disorders | Amphetamine | 8 | 0.00 | 0.00 | 0.48 | 0.42 |
| Tobacco Use Disorder | Cannabinoid | 10 | 0.00 | 0.00 | 0.43 | 0.81 |
| Epilepsy, Temporal Lobe | Tryptamine | 11 | 0.00 | 0.00 | 0.42 | 2.48 |
| Opioid-Related Disorders | Cannabinoid | 10 | 0.00 | 0.00 | 0.34 | 0.63 |
| Dementia | Cannabinoid | 13 | 0.00 | 0.00 | 0.34 | 0.30 |
| Epilepsy, Complex Partial | Tryptamine | 14 | 0.00 | 0.00 | 0.33 | 2.67 |
| Psychoses, Substance-Induced | Cannabinoid | 14 | 0.00 | 0.00 | 0.33 | 0.45 |
| Obsessive-Compulsive Disorder | Tryptamine | 13 | 0.00 | 0.00 | 0.31 | 3.12 |
| Epilepsy, Tonic-Clonic | Tryptamine | 20 | 0.00 | 0.00 | 0.30 | 2.17 |
| Schizophrenia, Paranoid | Tryptamine | 22 | 0.00 | 0.00 | 0.28 | 2.97 |
| Schizophrenia, Paranoid | Cathinone | 22 | 0.00 | 0.00 | 0.28 | 0.74 |
| Epilepsy, Absence | Cannabinoid | 12 | 0.00 | 0.00 | 0.27 | 0.26 |
| Epilepsies, Partial | Tryptamine | 19 | 0.00 | 0.00 | 0.26 | 2.12 |
| Amnesia | Cannabinoid | 44 | 0.00 | 0.00 | 0.11 | 0.20 |
| Dyspareunia | Phenethylamine | 2 | 0.00 | 0.00 | 0.00 | 6.57 |
| Myoclonic Epilepsy, Juvenile | Tryptamine | 3 | 0.00 | 0.00 | 0.00 | 5.00 |
| Narcolepsy | Tryptamine | 7 | 0.00 | 0.00 | 0.00 | 4.51 |
| Enuresis | Tryptamine | 7 | 0.00 | 0.00 | 0.00 | 3.83 |
| Dysthymic Disorder | Tryptamine | 6 | 0.00 | 0.00 | 0.00 | 3.67 |
| Tic Disorders | Amphetamine | 5 | 0.00 | 0.00 | 0.00 | 3.35 |
| Conduct Disorder | Tryptamine | 15 | 0.00 | 0.00 | 0.00 | 3.26 |
| Gambling | Cathinone | 1 | 0.00 | 0.00 | 0.00 | 3.03 |
| Pick Disease of the Brain | Tryptamine | 1 | 0.00 | 0.00 | 0.00 | 3.03 |
| Borderline Personality Disorder | Tryptamine | 4 | 0.00 | 0.00 | 0.00 | 2.66 |
| Binge-Eating Disorder | Tryptamine | 2 | 0.00 | 0.00 | 0.00 | 2.38 |
| Schizophrenia, Childhood | Tryptamine | 2 | 0.00 | 0.00 | 0.00 | 2.06 |
| Paraphilias | Tryptamine | 3 | 0.00 | 0.00 | 0.00 | 2.04 |
| Lewy Body Disease | Tryptamine | 2 | 0.00 | 0.00 | 0.00 | 2.03 |
| Dyspareunia | Other | 2 | 0.00 | 0.00 | 0.00 | 2.02 |
| Fetishism (Psychiatric) | Phenethylamine | 1 | 0.00 | 0.00 | 0.00 | 2.02 |
| Jet Lag Syndrome | Phenethylamine | 1 | 0.00 | 0.00 | 0.00 | 2.02 |
| Seasonal Affective Disorder | Phenethylamine | 1 | 0.00 | 0.00 | 0.00 | 2.02 |
| Sleep Disorders, Circadian Rhythm | Phenethylamine | 1 | 0.00 | 0.00 | 0.00 | 2.02 |
| Sleep-Wake Transition Disorders | Tryptamine | 1 | 0.00 | 0.00 | 0.00 | 2.02 |
| Eating Disorders | Tryptamine | 3 | 0.00 | 0.00 | 0.00 | 1.85 |
| Child Development Disorders, Pervasive | Tryptamine | 5 | 0.00 | 0.00 | 0.00 | 1.80 |
| Schizophrenia, Catatonic | Tryptamine | 5 | 0.00 | 0.00 | 0.00 | 1.72 |
| Morphine Dependence | Tryptamine | 5 | 0.00 | 0.00 | 0.00 | 1.65 |
| Dyspareunia | Tryptamine | 2 | 0.00 | 0.00 | 0.00 | 1.52 |
| Child Development Disorders, Pervasive | Phenethylamine | 5 | 0.00 | 0.00 | 0.00 | 1.50 |
| Impulse Control Disorders | Phenethylamine | 8 | 0.00 | 0.00 | 0.00 | 1.46 |
| Attention Deficit and Disruptive Behavior Disorders | Phenethylamine | 5 | 0.00 | 0.00 | 0.00 | 1.29 |
| Alcoholism | Amphetamine | 17 | 0.00 | 0.00 | 0.00 | 1.27 |
| Nocturnal Enuresis | Other | 4 | 0.00 | 0.00 | 0.00 | 1.19 |
| Alcoholic Intoxication | Amphetamine | 3 | 0.00 | 0.00 | 0.00 | 1.17 |
| Schizophrenia, Disorganized | Phenethylamine | 2 | 0.00 | 0.00 | 0.00 | 1.16 |
| Alcohol Withdrawal Seizures | Phenethylamine | 4 | 0.00 | 0.00 | 0.00 | 1.10 |
| Myoclonic Epilepsies, Progressive | Other | 3 | 0.00 | 0.00 | 0.00 | 1.03 |
| Aphasia, Broca | Other | 1 | 0.00 | 0.00 | 0.00 | 1.01 |
| Combat Disorders | Phenethylamine | 1 | 0.00 | 0.00 | 0.00 | 1.01 |
| Dementia, Multi-Infarct | Phenethylamine | 1 | 0.00 | 0.00 | 0.00 | 1.01 |
| Dementia, Vascular | Cathinone | 2 | 0.00 | 0.00 | 0.00 | 1.01 |
| Depression, Postpartum | Tryptamine | 1 | 0.00 | 0.00 | 0.00 | 1.01 |
| Fetishism (Psychiatric) | Tryptamine | 1 | 0.00 | 0.00 | 0.00 | 1.01 |
| Jet Lag Syndrome | Amphetamine | 1 | 0.00 | 0.00 | 0.00 | 1.01 |
| Jet Lag Syndrome | Other | 1 | 0.00 | 0.00 | 0.00 | 1.01 |
| Motor Skills Disorders | Tryptamine | 1 | 0.00 | 0.00 | 0.00 | 1.01 |
| Pick Disease of the Brain | Other | 1 | 0.00 | 0.00 | 0.00 | 1.01 |
| Psychoses, Alcoholic | Phenethylamine | 1 | 0.00 | 0.00 | 0.00 | 1.01 |
| Schizotypal Personality Disorder | Other | 2 | 0.00 | 0.00 | 0.00 | 1.01 |
| Seasonal Affective Disorder | Amphetamine | 1 | 0.00 | 0.00 | 0.00 | 1.01 |
| Seasonal Affective Disorder | Other | 1 | 0.00 | 0.00 | 0.00 | 1.01 |
| Sleep Deprivation | Other | 2 | 0.00 | 0.00 | 0.00 | 1.01 |
| Sleep Disorders, Circadian Rhythm | Amphetamine | 1 | 0.00 | 0.00 | 0.00 | 1.01 |
| Sleep Disorders, Circadian Rhythm | Other | 1 | 0.00 | 0.00 | 0.00 | 1.01 |
| Speech Disorders | Amphetamine | 2 | 0.00 | 0.00 | 0.00 | 1.01 |
| Wernicke Encephalopathy | Phenethylamine | 1 | 0.00 | 0.00 | 0.00 | 1.01 |
| Enuresis | Other | 7 | 0.00 | 0.00 | 0.00 | 0.96 |
| Schizophrenia and Disorders with Psychotic Features | Phenethylamine | 4 | 0.00 | 0.00 | 0.00 | 0.94 |
| Nocturnal Enuresis | Phenethylamine | 4 | 0.00 | 0.00 | 0.00 | 0.89 |
| Alcohol Withdrawal Seizures | Other | 4 | 0.00 | 0.00 | 0.00 | 0.83 |
| Disorders of Excessive Somnolence | Cathinone | 5 | 0.00 | 0.00 | 0.00 | 0.74 |
| Asperger Syndrome | Amphetamine | 2 | 0.00 | 0.00 | 0.00 | 0.67 |
| Asperger Syndrome | Phenethylamine | 2 | 0.00 | 0.00 | 0.00 | 0.67 |
| Asperger Syndrome | Tryptamine | 2 | 0.00 | 0.00 | 0.00 | 0.67 |
| Intellectual Disability | Amphetamine | 2 | 0.00 | 0.00 | 0.00 | 0.67 |
| Intellectual Disability | Phenethylamine | 2 | 0.00 | 0.00 | 0.00 | 0.67 |
| Intellectual Disability | Tryptamine | 2 | 0.00 | 0.00 | 0.00 | 0.67 |
| Huntington Disease | Cathinone | 10 | 0.00 | 0.00 | 0.00 | 0.66 |
| Nocturnal Enuresis | Cathinone | 4 | 0.00 | 0.00 | 0.00 | 0.59 |
| Schizophrenia, Disorganized | Amphetamine | 2 | 0.00 | 0.00 | 0.00 | 0.58 |
| Schizophrenia, Disorganized | Cathinone | 2 | 0.00 | 0.00 | 0.00 | 0.58 |
| Myoclonic Epilepsy, Juvenile | Other | 3 | 0.00 | 0.00 | 0.00 | 0.56 |
| Sexual Dysfunctions, Psychological | Cathinone | 11 | 0.00 | 0.00 | 0.00 | 0.52 |
| Schizophrenia, Childhood | Other | 2 | 0.00 | 0.00 | 0.00 | 0.52 |
| Myoclonic Epilepsies, Progressive | Cathinone | 3 | 0.00 | 0.00 | 0.00 | 0.51 |
| Paraphilias | Other | 3 | 0.00 | 0.00 | 0.00 | 0.51 |
| Lewy Body Disease | Other | 2 | 0.00 | 0.00 | 0.00 | 0.51 |
| Frontotemporal Dementia | Phenethylamine | 2 | 0.00 | 0.00 | 0.00 | 0.51 |
| Frontotemporal Dementia | Tryptamine | 2 | 0.00 | 0.00 | 0.00 | 0.51 |
| Hypochondriasis | Amphetamine | 2 | 0.00 | 0.00 | 0.00 | 0.51 |
| Hypochondriasis | Phenethylamine | 2 | 0.00 | 0.00 | 0.00 | 0.51 |
| Neonatal Abstinence Syndrome | Tryptamine | 2 | 0.00 | 0.00 | 0.00 | 0.51 |
| Disorders of Excessive Somnolence | Amphetamine | 5 | 0.00 | 0.00 | 0.00 | 0.50 |
| Schizophrenia and Disorders with Psychotic Features | Amphetamine | 4 | 0.00 | 0.00 | 0.00 | 0.47 |
| Schizophrenia and Disorders with Psychotic Features | Tryptamine | 4 | 0.00 | 0.00 | 0.00 | 0.47 |
| Alcohol Withdrawal Delirium | Phenethylamine | 8 | 0.00 | 0.00 | 0.00 | 0.43 |
| Morphine Dependence | Cannabinoid | 5 | 0.00 | 0.00 | 0.00 | 0.41 |
| Sexual Dysfunctions, Psychological | Other | 11 | 0.00 | 0.00 | 0.00 | 0.39 |
| Alcoholic Intoxication | Other | 3 | 0.00 | 0.00 | 0.00 | 0.39 |
| Schizophrenia, Catatonic | Phenethylamine | 5 | 0.00 | 0.00 | 0.00 | 0.34 |
| Anorexia Nervosa | Tryptamine | 3 | 0.00 | 0.00 | 0.00 | 0.34 |
| Amnesia, Anterograde | Cannabinoid | 4 | 0.00 | 0.00 | 0.00 | 0.30 |
| Seizures, Febrile | Cathinone | 8 | 0.00 | 0.00 | 0.00 | 0.27 |
| Disorders of Excessive Somnolence | Cannabinoid | 5 | 0.00 | 0.00 | 0.00 | 0.25 |
| Enuresis | Cathinone | 7 | 0.00 | 0.00 | 0.00 | 0.24 |
| Epilepsy, Generalized | Cathinone | 7 | 0.00 | 0.00 | 0.00 | 0.23 |
| Alzheimer Disease | Cannabinoid | 32 | 0.00 | 0.00 | 0.00 | 0.22 |
| Alcohol Withdrawal Delirium | Cathinone | 8 | 0.00 | 0.00 | 0.00 | 0.21 |
| Erectile Dysfunction | Cannabinoid | 26 | 0.00 | 0.00 | 0.00 | 0.21 |
| Amphetamine-Related Disorders | Cannabinoid | 27 | 0.00 | 0.00 | 0.00 | 0.18 |
| Autistic Disorder | Cannabinoid | 32 | 0.00 | 0.00 | 0.00 | 0.17 |
| Cognition Disorders | Cannabinoid | 26 | 0.00 | 0.00 | 0.00 | 0.17 |
| Delirium | Cannabinoid | 8 | 0.00 | 0.00 | 0.00 | 0.17 |
| Consciousness Disorders | Cannabinoid | 9 | 0.00 | 0.00 | 0.00 | 0.16 |
| Cataplexy | Cannabinoid | 12 | 0.00 | 0.00 | 0.00 | 0.15 |
| Stereotypic Movement Disorder | Cannabinoid | 16 | 0.00 | 0.00 | 0.00 | 0.11 |
| Status Epilepticus | Cannabinoid | 29 | 0.00 | 0.00 | 0.00 | 0.10 |
| Learning Disorders | Cannabinoid | 26 | 0.00 | 0.00 | 0.00 | 0.09 |
| Schizophrenia | Cannabinoid | 65 | 0.00 | 0.00 | 0.00 | 0.08 |
| Bipolar Disorder | Cannabinoid | 42 | 0.00 | 0.00 | 0.00 | 0.08 |
| Depressive Disorder | Cannabinoid | 65 | 0.00 | 0.00 | 0.00 | 0.07 |

## **Table S10**: One-tailed KS-Test p-values for the statistical tests introduced in Figures 2 and 3. The alternative hypothesis for all tests that the distribution tested has a greater cumulative distribution function than the randomized distributions.

|  | *Top 10* | *Top 25* | *Top 40* | *Top100* |
| --- | --- | --- | --- | --- |
| *Normalized indication rank against randomized indications* | 1.622e-14 | 7.392e-11 | 4.030e-10 | 4.449e-08 |
| *Normalized indication rank against randomized compounds* | 1.805e-35 | 1.805e-35 | 1.694e-29 | 8.553e-21 |
| *Normalized compound rank against randomized indications* | 1.960e-06 | 6.955e-10 | 2.445e-15 | 8.796e-13 |
| *Normalized compound rank against randomized compounds* | 1.504e-07 | 7.772e-17 | 1.476e-23 | 4.665e-26 |

## **Table S11**: One-tailed paired T-Test p-values for the statistical tests introduced in Figures 3. The alternative hypothesis for all tests that the distribution tested has a greater cumulative distribution function than the randomized distributions.

|  | *Top 10* | *Top 25* | *Top 40* | *Top100* |
| --- | --- | --- | --- | --- |
| *Normalized compound rank against randomized indications* | 7.846e-12 | 3.121e-18 | 2.250e-21 | 8.811e-37 |
| *Normalized compound rank against randomized compounds* | 1.397e-08 | 1.345e-10 | 1.904e-08 | 8.247e-16 |

## **Table S12**: Indication-Indication association counts for the Top10 predictions.

| Indication 1 | Indication 2 | Association |
| --- | --- | --- |
| Depressive Disorder, Major | Binge-Eating Disorder | 2 |
| Personality Disorders | Binge-Eating Disorder | 2 |
| Depressive Disorder, Major | Bipolar Disorder | 2 |
| Epilepsies, Myoclonic | Bipolar Disorder | 2 |
| Amnesia | Cataplexy | 3 |
| Depressive Disorder, Major | Cocaine-Related Disorders | 5 |
| Restless Legs Syndrome | Cocaine-Related Disorders | 3 |
| Seizures | Cocaine-Related Disorders | 2 |
| Anxiety Disorders | Depressive Disorder, Major | 2 |
| Epilepsies, Myoclonic | Depressive Disorder, Major | 2 |
| Amnesia | Epilepsy | 2 |
| Amnesia | Narcolepsy | 2 |
| Cataplexy | Narcolepsy | 2 |
| Epilepsies, Myoclonic | Narcolepsy | 2 |
| Bipolar Disorder | Personality Disorders | 4 |
| Depressive Disorder, Major | Personality Disorders | 3 |
| Epilepsies, Myoclonic | Personality Disorders | 2 |
| Depressive Disorder, Major | Seizures | 2 |
| Epilepsies, Myoclonic | Seizures | 2 |
| Cocaine-Related Disorders | Substance Withdrawal Syndrome | 3 |

**Table S13**: Indication-Indication association counts for the Top25 predictions.

| Indication 1 | Indication 2 | Association |
| --- | --- | --- |
| Amnesia | Anxiety Disorders | 2 |
| Depressive Disorder, Major | Anxiety Disorders | 2 |
| Cocaine-Related Disorders | Attention Deficit Disorder with Hyperactivity | 2 |
| Depressive Disorder, Major | Cocaine-Related Disorders | 10 |
| Epilepsies, Myoclonic | Cocaine-Related Disorders | 7 |
| Narcolepsy | Cocaine-Related Disorders | 2 |
| Seizures | Cocaine-Related Disorders | 9 |
| Depressive Disorder, Major | Epilepsies, Myoclonic | 7 |
| Depressive Disorder, Major | Narcolepsy | 3 |
| Epilepsies, Myoclonic | Narcolepsy | 3 |
| Depressive Disorder, Major | Seizures | 8 |
| Epilepsies, Myoclonic | Seizures | 10 |
| Narcolepsy | Seizures | 2 |
| Seizures | Sleep Initiation and Maintenance Disorders | 2 |
| Substance Withdrawal Syndrome | Sleep Initiation and Maintenance Disorders | 2 |
| Cocaine-Related Disorders | Substance Withdrawal Syndrome | 3 |
| Seizures | Substance Withdrawal Syndrome | 4 |

**Table S14**: Indication-Indication association counts for the Top40 predictions.

| Indication 1 | Indication 2 | Association |
| --- | --- | --- |
| Cocaine-Related Disorders | Alzheimer Disease | 2 |
| Seizures | Anxiety Disorders | 2 |
| Cocaine-Related Disorders | Attention Deficit Disorder with Hyperactivity | 2 |
| Anxiety Disorders | Cocaine-Related Disorders | 2 |
| Depressive Disorder, Major | Cocaine-Related Disorders | 10 |
| Seizures | Cocaine-Related Disorders | 13 |
| Anxiety Disorders | Depressive Disorder, Major | 4 |
| Seizures | Depressive Disorder, Major | 9 |
| Cocaine-Related Disorders | Learning Disorders | 3 |
| Seizures | Learning Disorders | 3 |
| Substance Withdrawal Syndrome | Learning Disorders | 3 |
| Depressive Disorder, Major | Narcolepsy | 2 |
| Cocaine-Related Disorders | Psychotic Disorders | 3 |
| Depressive Disorder, Major | Psychotic Disorders | 2 |
| Seizures | Psychotic Disorders | 2 |
| Anxiety Disorders | Schizophrenia | 2 |
| Cocaine-Related Disorders | Schizophrenia | 2 |
| Depressive Disorder, Major | Schizophrenia | 2 |
| Psychotic Disorders | Schizophrenia | 2 |
| Seizures | Schizophrenia | 2 |
| Sleep Initiation and Maintenance Disorders | Schizophrenia | 2 |
| Cocaine-Related Disorders | Sleep Initiation and Maintenance Disorders | 2 |
| Depressive Disorder, Major | Sleep Initiation and Maintenance Disorders | 2 |
| Psychotic Disorders | Sleep Initiation and Maintenance Disorders | 2 |
| Seizures | Sleep Initiation and Maintenance Disorders | 2 |
| Cocaine-Related Disorders | Substance Withdrawal Syndrome | 5 |
| Depressive Disorder, Major | Substance Withdrawal Syndrome | 2 |
| Psychotic Disorders | Substance Withdrawal Syndrome | 2 |
| Schizophrenia | Substance Withdrawal Syndrome | 2 |
| Seizures | Substance Withdrawal Syndrome | 5 |
| Sleep Initiation and Maintenance Disorders | Substance Withdrawal Syndrome | 2 |

**Table S15**: Indication-Indication association counts for the Top100 predictions.

| Indication 1 | Indication 2 | Association |
| --- | --- | --- |
| Amnesia | Alzheimer Disease | 2 |
| Cocaine-Related Disorders | Alzheimer Disease | 3 |
| Seizures | Alzheimer Disease | 2 |
| Cocaine-Related Disorders | Amnesia | 4 |
| Amnesia | Anxiety Disorders | 2 |
| Cocaine-Related Disorders | Anxiety Disorders | 11 |
| Psychotic Disorders | Anxiety Disorders | 2 |
| Cocaine-Related Disorders | Attention Deficit Disorder with Hyperactivity | 5 |
| Seizures | Attention Deficit Disorder with Hyperactivity | 3 |
| Amnesia | Autistic Disorder | 3 |
| Anxiety Disorders | Autistic Disorder | 4 |
| Cocaine-Related Disorders | Autistic Disorder | 8 |
| Anxiety Disorders | Depressive Disorder | 2 |
| Cocaine-Related Disorders | Depressive Disorder | 4 |
| Depressive Disorder, Major | Depressive Disorder | 2 |
| Psychotic Disorders | Depressive Disorder | 2 |
| Schizophrenia | Depressive Disorder | 2 |
| Seizures | Depressive Disorder | 4 |
| Substance Withdrawal Syndrome | Depressive Disorder | 2 |
| Anxiety Disorders | Depressive Disorder, Major | 7 |
| Autistic Disorder | Depressive Disorder, Major | 2 |
| Cocaine-Related Disorders | Depressive Disorder, Major | 12 |
| Psychotic Disorders | Depressive Disorder, Major | 2 |
| Seizures | Depressive Disorder, Major | 6 |
| Cocaine-Related Disorders | Psychotic Disorders | 7 |
| Anxiety Disorders | Schizophrenia | 3 |
| Attention Deficit Disorder with Hyperactivity | Schizophrenia | 2 |
| Cocaine-Related Disorders | Schizophrenia | 5 |
| Psychotic Disorders | Schizophrenia | 3 |
| Seizures | Schizophrenia | 4 |
| Amnesia | Seizures | 7 |
| Anxiety Disorders | Seizures | 9 |
| Autistic Disorder | Seizures | 6 |
| Cocaine-Related Disorders | Seizures | 23 |
| Psychotic Disorders | Seizures | 5 |
| Anxiety Disorders | Sleep Disorders | 2 |
| Cocaine-Related Disorders | Sleep Disorders | 2 |
| Depressive Disorder | Sleep Disorders | 2 |
| Psychotic Disorders | Sleep Disorders | 2 |
| Schizophrenia | Sleep Disorders | 2 |
| Seizures | Sleep Disorders | 2 |
| Sleep Initiation and Maintenance Disorders | Sleep Disorders | 2 |
| Anxiety Disorders | Sleep Initiation and Maintenance Disorders | 2 |
| Cocaine-Related Disorders | Sleep Initiation and Maintenance Disorders | 2 |
| Depressive Disorder | Sleep Initiation and Maintenance Disorders | 2 |
| Psychotic Disorders | Sleep Initiation and Maintenance Disorders | 2 |
| Schizophrenia | Sleep Initiation and Maintenance Disorders | 2 |
| Seizures | Sleep Initiation and Maintenance Disorders | 2 |
| Cocaine-Related Disorders | Substance Withdrawal Syndrome | 5 |
| Depressive Disorder, Major | Substance Withdrawal Syndrome | 2 |
| Psychotic Disorders | Substance Withdrawal Syndrome | 2 |
| Schizophrenia | Substance Withdrawal Syndrome | 2 |
| Seizures | Substance Withdrawal Syndrome | 5 |

# **References**

1. Haslam, C. *et al.* The Evolution of MALDI-TOF Mass Spectrometry toward Ultra-High-Throughput Screening: 1536-Well Format and Beyond. *J. Biomol. Screen.* **21**, 176–186 (2016).

2. Pérez-Moreno, G. *et al.* Discovery of New Compounds Active against Plasmodium falciparum by High Throughput Screening of Microbial Natural Products. *PLoS One* **11**, e0145812 (2016).

3. Sinclair, I. *et al.* Novel Acoustic Loading of a Mass Spectrometer: Toward Next-Generation High-Throughput MS Screening. *J. Lab. Autom.* **21**, 19–26 (2016).

4. Sun, X., Vilar, S. & Tatonetti, N. P. High-Throughput Methods for Combinatorial Drug Discovery. *Sci. Transl. Med.* **5**, (2013).

5. Jenwitheesuk, E., Horst, J. A., Rivas, K. L., Van Voorhis, W. C. & Samudrala, R. Novel paradigms for drug discovery: computational multitarget screening. *Trends Pharmacol. Sci.* **29**, 62–71 (2008).

6. Pink, R., Hudson, A., Mouriès, M.-A. & Bendig, M. Opportunities and Challenges in Antiparasitic Drug Discovery. *Nat. Rev. Drug Discov.* **4**, 727–740 (2005).

7. Lombardino, J. G. & Lowe, J. A. The role of the medicinal chemist in drug discovery--then and now. *Nat Rev Drug Discov* **3**, 853–862 (2004).

8. Kar, S. & Roy, K. How far can virtual screening take us in drug discovery? *Expert Opin. Drug Discov.* **8**, 245–261 (2013).

9. Bottegoni, G., Favia, A. D., Recanatini, M. & Cavalli, A. The role of fragment-based and computational methods in polypharmacology. *Drug Discov. Today* **17**, 23–34 (2012).

10. Perola, E., Walters, W. P. & Charifson, P. S. A detailed comparison of current docking and scoring methods on systems of pharmaceutical relevance. *Proteins Struct. Funct. Bioinforma.* **56**, 235–249 (2004).

11. Ou-Yang, S. *et al.* Computational drug discovery. *Acta Pharmacol. Sin.* **33**, 1131–1140 (2012).

12. Deng, Z., Chuaqui, C. & Singh, J. Structural Interaction Fingerprint (SIFt):  A Novel Method for Analyzing Three-Dimensional Protein−Ligand Binding Interactions. *J. Med. Chem.* **47**, 337–344 (2004).

13. Pouliot, Y., Chiang, A. P. & Butte, A. J. Predicting Adverse Drug Reactions Using Publicly Available PubChem BioAssay Data. *Clin. Pharmacol. Ther.* **90**, 90–99 (2011).

14. Bernard, B. & Samudrala, R. A generalized knowledge-based discriminatory function for biomolecular interactions. *Proteins Struct. Funct. Bioinforma.* (2009). doi:10.1002/prot.22323

15. Yuriev, E. & Ramsland, P. A. Latest developments in molecular docking: 2010-2011 in review. *J. Mol. Recognit.* **26**, 215–239 (2013).

16. Yuriev, E., Holien, J. & Ramsland, P. A. Improvements, trends, and new ideas in molecular docking: 2012-2013 in review. *Journal of Molecular Recognition* (2015). doi:10.1002/jmr.2471

17. Grigoryan, A. V., Wang, H. & Cardozo, T. J. Can the Energy Gap in the Protein-Ligand Binding Energy Landscape Be Used as a Descriptor in Virtual Ligand Screening? *PLoS One* **7**, e46532 (2012).

18. Trott, O. & Olson, A. J. AutoDock Vina: improving the speed and accuracy of docking with a new scoring function, efficient optimization, and multithreading. *J. Comput. Chem.* **31**, 455–61 (2010).

19. Verdonk, M. L., Cole, J. C., Hartshorn, M. J., Murray, C. W. & Taylor, R. D. Improved protein-ligand docking using GOLD. *Proteins Struct. Funct. Bioinforma.* **52**, 609–623 (2003).

20. Jenwitheesuk, E. & Samudrala, R. Identification of Potential Multitarget Antimalarial Drugs. *JAMA* **294**, 1487 (2005).

21. Tubert-Brohman, I., Sherman, W., Repasky, M. & Beuming, T. Improved Docking of Polypeptides with Glide. *J. Chem. Inf. Model.* **53**, 1689–1699 (2013).

22. Carlson, H. A. *et al.* CSAR 2014: A Benchmark Exercise Using Unpublished Data from Pharma. *J. Chem. Inf. Model.* **56**, 1063–1077 (2016).

23. Damm-Ganamet, K. L., Smith, R. D., Dunbar, J. B., Stuckey, J. A. & Carlson, H. A. CSAR benchmark exercise 2011-2012: Evaluation of results from docking and relative ranking of blinded congeneric series. *J. Chem. Inf. Model.* **53**, 1853–1870 (2013).

24. Jenwitheesuk, E. *et al.* Improved prediction of HIV-1 protease-inhibitor binding energies by molecular dynamics simulations. *BMC Struct. Biol. 2003 31* **40**, 299–309 (2003).

25. Wang, Z. *et al.* Comprehensive evaluation of ten docking programs on a diverse set of protein-ligand complexes: the prediction accuracy of sampling power and scoring power. *Phys. Chem. Chem. Phys.* **18**, 12964–12975 (2016).

26. Carlson, H. A. & McCammon, J. A. Accommodating protein flexibility in computational drug design. *Mol. Pharmacol.* **57**, 213–8 (2000).

27. Cross, J. B. *et al.* Comparison of Several Molecular Docking Programs: Pose Prediction and Virtual Screening Accuracy. *J. Chem. Inf. Model.* **49**, 1455–1474 (2009).

28. Biesiada, J., Porollo, A., Velayutham, P., Kouril, M. & Meller, J. Survey of public domain software for docking simulations and virtual screening. *Hum. Genomics* **5**, 497–505 (2011).

29. Brewerton, S. C. The use of protein-ligand interaction fingerprints in docking. *Curr. Opin. Drug Discov. Devel.* **11**, 356–64 (2008).

30. Huang, S.-Y. & Zou, X. Ensemble docking of multiple protein structures: Considering protein structural variations in molecular docking. *Proteins Struct. Funct. Bioinforma.* **66**, 399–421 (2006).

31. Huang, S.-Y. & Zou, X. Efficient molecular docking of NMR structures: application to HIV-1 protease. *Protein Sci.* **16**, 43–51 (2007).

32. Friesner, R. A. *et al.* Glide:  A New Approach for Rapid, Accurate Docking and Scoring. 1. Method and Assessment of Docking Accuracy. *J. Med. Chem.* **47**, 1739–1749 (2004).

33. Wong, C. F. Flexible receptor docking for drug discovery. *Expert Opin. Drug Discov.* **10**, 1189–1200 (2015).

34. Kuenemann, M. A. *et al.* In silico design of low molecular weight protein-protein interaction inhibitors: Overall concept and recent advances. *Progress in Biophysics and Molecular Biology* **119**, 20–32 (2015).

35. Hao, G.-F. *et al.* Computational discovery of picomolar Q(o) site inhibitors of cytochrome bc1 complex. *J. Am. Chem. Soc.* **134**, 11168–76 (2012).

36. Yang, T. *et al.* Virtual screening using molecular simulations. *Proteins Struct. Funct. Bioinforma.* **79**, 1940–1951 (2011).

37. Minie, M. *et al.* CANDO and the infinite drug discovery frontier. *Drug Discovery Today* (2014). doi:10.1016/j.drudis.2014.06.018

38. Sethi, G., Chopra, G. & Samudrala, R. Multiscale Modelling of Relationships between Protein Classes and Drug Behavior Across all Diseases Using the CANDO Platform. *Mini Rev. Med. Chem.* **15**, 705–717 (2015).

39. Chopra, G. & Samudrala, R. Exploring Polypharmacology in Drug Discovery and Repurposing Using the CANDO Platform. *Curr. Pharm. Des.* **22**, 3109–23 (2016).

40. O’Boyle, N. M. *et al.* Open Babel: An open chemical toolbox. *J. Cheminform.* **3**, 33 (2011).
